# Adaptive Coordination Engineering for Efficient and Robust H_2_ Release

**DOI:** 10.1002/advs.76028

**Published:** 2026-06-11

**Authors:** Xiaolin Jiang, Xiaoyang Yan, Shenglong Jiang, Hongdan Zhu, Heng Wang, Xiaopeng Li, Xiaochun Zhou, Mingdong Zhou, Qian Peng

**Affiliations:** ^1^ Digital and Intelligent Empowerment Biomedical Innovation Center School of Pharmacy Shanghai University of Medicine and Health Sciences Shanghai P. R. China; ^2^ State Key Laboratory of Elemento‐organic Chemistry Tianjin Key Laboratory of Biosensing and Molecular Recognition College of Chemistry Frontiers Science Center For New Organic Matter Nankai University Tianjin P. R. China; ^3^ College of Chemical Engineering Shenyang University of Chemical Technology Shenyang P. R. China; ^4^ State Key Laboratory of Organometallic Chemistry Shanghai Institute of Organic Chemistry Chinese Academy of Sciences (CAS) Shanghai P. R. China; ^5^ Division of Advanced Nanomaterials Suzhou Institute of Nano‐tech and Nano‐bionics Chinese Academy of Sciences (CAS) Suzhou P. R. China

**Keywords:** bimetallic catalysis, formic acid dehydrogenation, metal formate, β‐H elimination

## Abstract

Efficient regulation of β‐H activation and its subsequent elimination remains a notorious challenge owing to stringent inherent geometric and electronic constraints. To overcome the high kinetic barriers, it necessitates a precise catalyst to effectively orchestrate the metal‐substrate interaction. Herein, we report a molecular Pd_1_‐Ag_2_ model using a tridentate phosphine ligand (**L2**) and formate bridges, which integrates the effect of double silver synergy and nitrate anion regulation, and serves as the core origin to facilitate efficient β‐H elimination. Such bimetallic cooperation optimizes the reaction pathway and realizes rapid hydrogen production with complete CO‐free characteristics, endowing prominent industrial advantages over monometallic counterparts. Under ambient air at 60°C, this catalyst delivers excellent activity and satisfactory recyclability. DFT calculations confirm that the rate‐determining β‐H elimination step of monometallic Pd systems is effectively optimized, and in situ spectroscopic measurements further verify the stable proximal Pd‐Ag synergistic coordination throughout the reaction.

## Graphical abstract

β‐H elimination is a pivotal but challenging step in homogeneous formic acid dehydrogenation reactions due to strict hydride‐metal matching. By using a rationally designed Pd_1_Ag_2_ bimetallic system with adaptive Pd_1_‐Ag_2_/**L2** coordination and formate bridges, it enables boosted formic acid dehydrogenation under ambient air (7.7 times higher than a Pd catalyst) by lowering the β‐H elimination barrier from 29.8 to 12.9 kcal/mol via Ag‐Pd synergy.

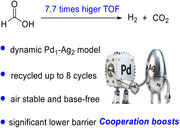

## 1 Introduction

Hydrogen, as a clean and green energy source, has attracted growing attention in recent years. However, its storage and transportation challenges hinder both large‐scale and portable applications. Among various hydrogen carriers, formic acid (FA) stands out for its convenience, recyclability, and sustainability, with a hydrogen capacity of 4.4 wt.% (53 g H_2_/L) [1]. The equilibrium between CO_2_/H_2_ and formic acid also makes it an ideal candidate for constructing a high‐efficiency H_2_ battery [2]. Dehydrogenation of HCOOH using efficient, recyclable catalysts is thus a key route for hydrogen production, making the development of practical and reliable H_2_‐generation systems from formic acid highly significant.

In FA dehydrogenation, both heterogeneous and homogeneous catalysts have been explored [3, 4, 5, 6], but hindered by β‐H elimination step as a critical kinetic barrier due to inherent spatial (O,O‐chelating) and electronic mismatches between the hydride (H) and metal active sites [7]. This kinetic barrier severely restricts overall catalytic efficiency, even in systems with well‐defined active sites. Despite efforts to address this, existing homogeneous organometallic complexes [8, 9, 10, 11, 12] (e.g., phosphine‐ or nitrogen‐ligated systems) have not fully overcome this bottleneck. The activation of formate species and promotion of β‐H elimination strongly depend on the geometric and electronic properties of active sites, and their molecular‐level mechanisms, including how metal–metal interactions in catalysts modulate reactivity, remain elusive. This lack of clarity hinders rational optimization of practical systems. Meanwhile, most existing catalytic systems rely on large amounts of basic amine additives to activate the reaction or stabilize key intermediates [13, 14].

To address the demand for base‐free systems, diverse strategies have emerged, including the construction of specialized ligands, utilization of Lewis acid auxiliaries, and employment of ionic liquids as reaction media, all aimed at promoting FA dehydrogenation [15, 16, 17, 18, 19]. Lewis acids enhance reactivity by coordinating with formate intermediates, weakening the C─H bond and facilitating hydride transfer [17], while ionic liquids modulate the reaction microenvironment to stabilize charged intermediates [18, 19]. These elegant developments related to β‐H elimination indeed led to high reactivities. However, limitations derived from sensitivity to air and by‐product CO still need to be overcome to reach the requirement of scalable application in industry. CO, as the by‐product of HCOOH dehydration, is a key competing reaction in the HCOOH dehydrogenation, especially considering its undesired poisoning effect for transition metals. Thus, developing a base‐free, air‐stable, and water‐tolerant catalytic system capable of efficient FA dehydrogenation under mild conditions by overcoming the β‐H elimination barrier remains a pressing challenge.

Palladium, which readily forms catalytically active Pd‐H intermediates [20, 21], is a promising candidate for dehydrogenation but remains underutilized in homogeneous FA dehydrogenation. Reported Pd‐based systems require a base (e.g., HCOONa) to activate reactions or stabilize active species, yet still show inadequate activity (TOF ≈ 28 h^−1^; TON ≈ 80) [22], air‐unstable and Pd^0^ aggregation that limit practical application. Notably, bimetallic complexes such as dimer catalysts are relatively stable, and metal–metal synergy is of great significance in improving the activity of homogeneous catalytic formic acid dehydrogenation [23, 24]. Phosphine‐ligated Pd complexes developed by Fleischer et al. have shown viability in transfer hydrogenation using FA [25], which hints that ligand and metal synergies could promote activity, but their role in β‐H elimination remains uncharacterized. Therefore, it is still needed to develop general strategies to overcome the undesired chelating effect of the formate anion during the β‐H elimination step. These findings inspire us to explore whether adaptive coordination of tridentate ligands to both Pd and Ag cations can overcome the β‐H elimination kinetic barrier in homogeneous FA dehydrogenation. Herein, we propose the use of an adaptive coordination strategy featuring tridentate ligands to lower the intrinsic kinetic barrier of β‐H elimination.

**SCHEME 1:**
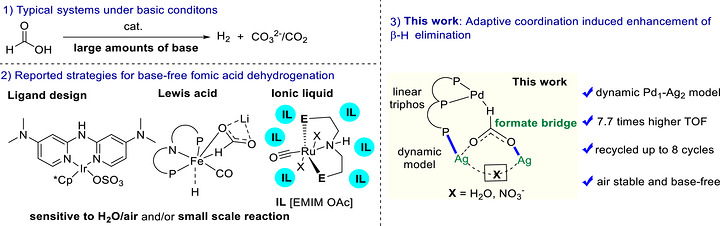
Homogeneous systems for dehydrogenation of formic acid.

In this study, we report a homogeneous Pd‐Ag bimetallic catalyst that addresses these challenges for formic acid dehydrogenation under base‐free, ambient air conditions. By combining linear triphos ligand (**L2**)‐stabilized Pd/Ag centers with adaptive coordination, this system achieves efficient FA dehydrogenation under air (Scheme 1). Specifically, the Ag atom not only stabilizes Pd against aggregation and air inactivation but also significantly lowers the energy barrier for β‐H elimination via synergy. This work delivers a high‐performance catalyst (TOF = 542.4 h^−1^, 7.7‐times higher than that of monometallic systems) and provides molecular‐level insights into bimetallic synergy in dehydrogenation, guiding the design of next‐generation catalysts.

## 2 Results and Discussion

Initially, we explored the catalytic activity of simple palladium salts for formic acid dehydrogenation (Table S1). Among the tested Pd salts, Pd(OAc)_2_ showed the most prominent activity at 60°C under a nitrogen atmosphere. But its turnover frequency (TOF) remained low (11.7 h^−1^; Table 1, entry 1), and the catalyst rapidly deactivated, likely due to irreversible Pd(0) aggregation into inactive Pd black. Given that ligand structure critically impacts catalyst activity and stability [16], we then screened ligands to enhance performance in homogeneous Pd‐catalyzed FA dehydrogenation. Among tested ligands, phosphine‐containing ones outperformed nitrogen‐containing counterparts (Table S2). Triphenylphosphine **L1**, a classic monodentate phosphine, effectively stabilized the Pd center against aggregation, resulting in a noticeable activity increase (TOF = 58.9 h^−1^; Table 1, entry 2), confirming its role in coordinating and protecting Pd from deactivation. When switching to the tridentate linear triphos **L2**, a slight but meaningful further increase in activity was observed (TOF = 88.4 h^−1^; Table 1, entry 3), suggesting that it operates via a similar coordination stabilization mechanism while offering additional geometric advantages for substrate binding. In contrast, the tridentate tripodal triphos **L3** showed lower activity (TOF = 32.6 h^−1^; Table 1, entry 4), despite reports that its Co complex exhibits high efficiency in this reaction [12]. Bidentate phosphines provided moderate activity but poor longevity (Table S3). The ligand effect likely stems from linear triphos’ ability to form tunable metal‐hydride complexes as observed in reductive amination [26], hinting at its potential to stabilize key intermediates in FA dehydrogenation.

**TABLE 1:** Pd‐catalyzed dehydrogenation of formic acid with different ligands ^a^.

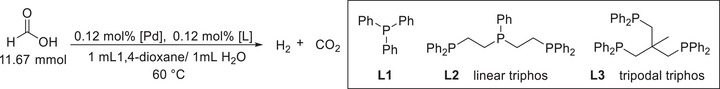						
Entry	[Pd]	[L]	Additive ^b^	Atmosphere	HCOONa ^c^	TOF
1	Pd(OAc)_2_	—	—	N_2_	HCOONa	11.7
2	Pd(OAc)_2_	L1	—	N_2_	HCOONa	58.9
3	Pd(OAc)_2_	L2	—	N_2_	HCOONa	88.4
4	Pd(OAc)_2_	L3	—	N_2_	HCOONa	32.6
5	Pd(OAc)_2_	L2	—	N_2_	—	77.5
6	Pd(OAc)_2_	L2	—	air	—	62.3
7 ^d^	Pd(OAc)_2_	L2	AgNO_3_	air	—	239.5
8	Pd(OAc)_2_	L2	AgNO_3_	air	—	542.4
9	Pd(OAc)_2_	L2	H_2_O_2_ (200 µL)	air	—	485.6
10	Pd(OAc)_2_	L2	LiBF_4_	air	—	132.8
11	Pd(OAc)_2_	L2	Zn(NO_3_)_2_	air	—	90.3
12	Pd(OAc)_2_	L2	Cu(NO_3_)_2_	air	—	74.6
13	Pd(OAc)_2_	L2	Al(NO_3_)_3_	air	—	103.3
14 ^e^	Pd(OAc)_2_	L2	AgNO_3_	air	—	706.9

^a^ Reaction conditions: 11.67 mmol FA, 2 mL solvent (1,4‐dioxane:H_2_O = 1:1); TOF(max) is obtained for the 20 min period of the reaction.

^b^ 0.24 mol%.

^c^ 0.1 mmol.

^d^ 0.12 mol% AgNO_3_.

^e^ 80°C.

Palladium was not well‐applied in homogeneous formic acid dehydrogenation reactions, even though it holds a critical role in C─H activation, coupling, and oxidation reactions [27, 28]. It is still struggling to overcome the β‐H elimination barrier and achieve efficient Pd‐catalyzed formic acid dehydrogenation. Notably, in this system, the use of HCOONa is not necessary, and the TOF result only showed a slight decrease (Table 1, entry 5 vs. 3), indicating that base is not the primary bottleneck here. Nevertheless, the reaction mixture quickly turned dark brown within 30 min due to the formation of Pd black as the inactive heterogeneous by‐product. Clearly, the lack of a metal synergist to stabilize Pd and optimize hydride transfer was the key limitation.

This bottleneck was fully addressed by the addition of Ag salts‐a critical, irreplaceable component for boosting activity and stability. When we tested Li^+^, Zn^2+^, Cu^2+^, or Al^3+^ salts as alternatives, all yielded far lower efficiency (TOF ≤ 132.8 h^−1^; Table 1, entries 10–13), with Zn^2+^ and Cu^2+^ even accelerating Pd aggregation (Tables S7–S9). In stark contrast, adding AgNO_3_ triggered a dramatic activity surge, paired with the linear triphos ligand **L2** and a H_2_O‐dioxane mixed solvent. The system achieved a TOF of 542.4 h^−1^ (Table 1, entry 8; with 39% conversion in 20 min) when using 2 equiv. of Ag salt compared to [Pd], which is 45‐times higher than the Pd‐only system without ligand (11.7 h^−1^) (Table 1, entry 1). The enhanced stability was also observed; unlike the Pd‐only system, the Pd_1_‐Ag_2_/**L2** catalyst showed no sign of deactivation, and the reaction mixture remained clear pale yellow (no Pd black formation) even after 48 h.

Solvent choice significantly impacts reaction thermodynamics and reactivity [29, 30]. Pure dioxane and water both served as suitable reaction media but yielded relatively low activity (Table S10). In contrast, a mixed solvent exhibited the highest activity. Meanwhile, the mixed solvent not only improves the dissolution of the organic ligands, but also optimizes the solubility of the salt additive, remedying the poor catalytic performance observed in single‐solvent media (Table S10). Critically, the system is air‐stable, and good yields were obtained under ambient air. It even tolerates H_2_O_2_ (TOF = 485.6 h^−1^; Table 1, entry 9). The activity increased to 706.9 h^−1^ at 80°C (Table 1, entry 14). In our system, no CO was detected by GC (Figure S1), which highlights its practical potential. Furthermore, 1 mL of CO (10 000 ppm) was additionally introduced into the reaction system, and the reaction activity could still be maintained for more than 1 h. This enhancement may arise from Pd‐Ag redox coupling. Ag^+^ (E^°^ = +0.80 V vs. SHE) is reduced to Ag^0^ by FA, which in turn reduces Pd^2+^ (E^°^ = +0.95 V vs. SHE) to regenerate Ag^+^ [31, 32]. Coordinated Ag^+^ avoids direct reaction with FA, stabilizing the Pd‐Ag system. Cyclic voltammetry (2.0 to −2.0 V, Figure S17) confirmed enhanced redox capacity in the Pd_1_‐Ag_2_/**L2** system vs. Pd/**L2** alone, which might be attributed to Ag‐Pd synergetic interaction to dramatically lower kinetic barriers.

**FIGURE 1:**
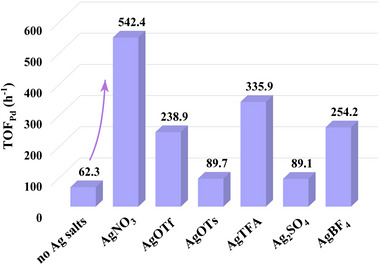
Pd‐catalyzed dehydrogenation of formic acid with different additives. Reaction conditions: 11.67 mmol FA, 2 mL solvent (V1,4‐dioxane:VH_2_O = 1:1), 0.12 mol% Pd(OAc)_2_, 0.12 mol% linear‐triphos (**L2**), and 0.24 mol% additive, 60°C; TOF is obtained for the 20 min period of the reaction.

Then, we focused on the effect of Ag salts. Specifically, we expanded our screening to other Ag salts (AgBF_4_, Ag_2_SO_4_, AgOTf) to assess whether the promotional effect is universal to Ag^+^. As shown in Figure 1, all tested Ag salts induced a measurable activity boost compared to the Pd‐only system. This confirms that Ag^+^ itself is the key synergist, as all Ag salts shared the ability to mitigate Pd aggregation and enhance β‐H elimination. However, none matched the performance of AgNO_3_. This counterion‐dependent activity further emphasizes that AgNO_3_ is not just a “source of Ag^+^” but an integral part of the catalytic design, with nitrate synergizing with Ag^+^ to maximize performance.

**FIGURE 2:**
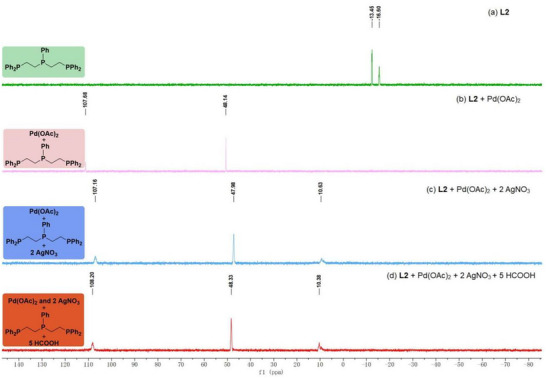
^31^P NMR spectra in CDCl_3_. (a) Linear triphos (**L2**); (b) After complexation of **L2** with Pd(OAc)_2_; (c) After complexation of **L2** with Pd(OAc)_2_ and AgNO_3_; (d) Reaction mixture (**L2** with Pd(OAc)_2_ and AgNO_3_ + HCOOH).

To unravel Ag's role in enhancing the activity of formic acid dehydrogenation, we combined in situ NMR, UV–vis, FT‐IR, and Raman spectroscopy analysis. First, in situ ^31^P NMR experiments were performed to explore the mechanism of the Pd_1_‐Ag_2_/**L2** system. ^31^P NMR measurements were used to reveal the ligand coordination behaviors. The chemical shifts are recorded for free **L2** (−13.45, −16.60 ppm), Pd‐bound **L2** (107.68, 48.14 ppm), and Ag‐bound **L2** (15.03, 10.70 ppm; Figure 2a–c), respectively. Mixing Pd(OAc)_2_, AgNO_3_, and **L2** yielded peaks from both Pd‐ and Ag‐bound ligand (107.16, 47.98, 10.63 ppm), with FA addition slightly shifting peaks but preserving the main coordination signals (Figure 2d). Such well‐maintained coordination environments reveal the formation of a stable bimetallic Pd‐Ag/ligand coordination structure under catalytic conditions, which rationalizes the robust structural stability and favorable recyclability observed during formic acid dehydrogenation. Meanwhile, ^1^H NMR results provided key mechanistic insights. In the absence of Ag^+^, a negligible Pd‐H signal suggested low efficiency of Pd‐H formation. Upon addition of **L2**, a small Pd‐H signal appeared at −8.21 ppm in the presence of formic acid. Notably, the addition of 1 or 2 equiv. of AgNO_3_ increased Pd‐H intensity (integral ratios 1:1.5 and 1:2.5) and induced a new Pd‐H species at −8.86 ppm, which probably can be assigned to a Pd‐Ag‐H intermediate (Figures S5–S7). This confirms Ag^+^ promotes Pd‐H formation and critically lowers the barrier for β‐H elimination, which is responsible for the markedly improved catalytic activity toward formic acid dehydrogenation.

**FIGURE 3:**
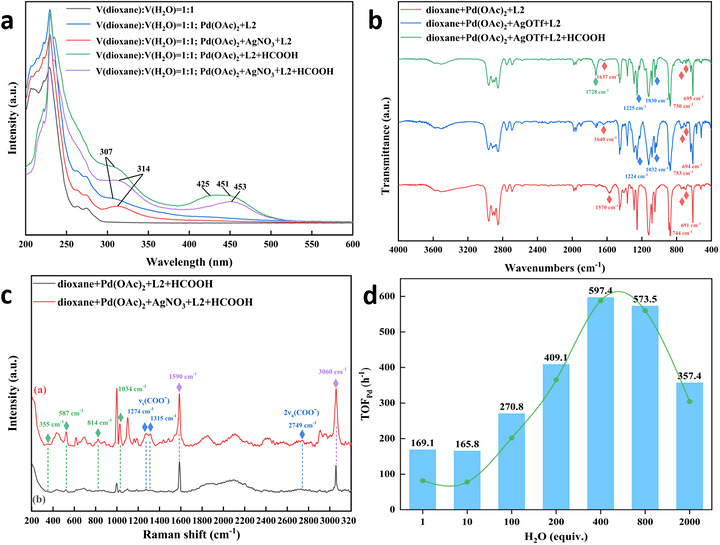
(a) UV–vis spectrum. UV–vis spectrum of blank solvent (V1,4‐dioxane:VH_2_O = 1:1); Pd(OAc)_2_ and **L2** in solvent; AgNO_3_, Pd(OAc)_2_ and **L2** in solvent; Pd(OAc)_2_, **L2** and HCOOH in solvent; AgNO_3_, Pd(OAc)_2_, **L2** and HCOOH in solvent. (b) In situ FT‐IR experiment using AgOTf. FT‐IR spectrum of Pd(OAc)_2_ and **L2** in 1,4‐dioxane; Pd(OAc)_2_, AgOTf and **L2** in 1,4‐dioxane; Pd(OAc)_2_, AgOTf, **L2** and HCOOH in 1,4‐dioxane. (c) Raman spectrum collected of (i) Pd(OAc)_2_ and **L2** in solvent (V1,4‐dioxane:VH_2_O = 1:1) with HCOOH; (ii) Pd(OAc)_2_, AgNO_3_, and **L2** in solvent (V1,4‐dioxane:VH_2_O = 1:1) with HCOOH. (d) Effect of water on the dehydrogenation rate. Reaction conditions: FA (1 mL), Pd(OAc)_2_ (0.075 mmol), AgNO_3_ (0.150 mmol), **L2** (0.075 mmol), dioxane (5 mL), H_2_O (x equiv. of [Pd]), 70°C.

Then, the UV–vis spectrum of a 1:1 dioxane/water blank solvent showed absorption only between 200 and 250 nm, attributed to inherent solvent absorption. UV–vis showed a ligand‐to‐metal charge transfer peak at 307 nm for Pd/**L2**, red‐shifting to 314 nm with AgNO_3_ might be evidence of Ag‐Pd electronic interaction. The 307→314 nm shift likely stems from AgNO_3_ introduction, which might increase weakly coordinated nitrate ions and cause Ag^+^ to compete with Pd^2+^, altering steric and electronic properties, thereby optimizing the coordination environment for reaction intermediates. Adding FA to Pd/**L2** generated a 453 nm peak (metal‐formate coordination), while AgNO_3_ addition induced a 425 nm peak, confirming new Ag‐containing intermediates (Figure 3a). New peaks at 453 and 425 nm suggest that the incorporation of FA leads to the formation of new species, presumably nitrate‐ and Ag^+^‐containing complexes that contribute significantly to the outstanding catalytic activity for FA dehydrogenation.

Next, in situ Fourier Transform Infrared (FT‐IR) spectroscopy was employed. In dioxane, adding linear triphos **L2** and Pd(OAc)_2_ generated new absorption peaks at 1570, 744, and 691 cm^−1^ (red line), which can be assigned to the formation of Pd/**L2** complexes (Figure 3b). These signals are distinct from the absorption bands of Pd(OAc)_2_ alone (1605 cm^−1^) and **L2** alone (745, 700 cm^−1^; Figure S16). Adding AgOTf yielded peaks at 1640, 753, and 694 cm^−1^, corresponding to the formation of the Pd–Ag/**L2** bimetallic species. The slight peak shifts relative to Pd/**L2** demonstrate the structural changes induced by Ag^+^, which modifies the electronic and coordination environment of Pd active centers. Additional peaks at 1224 and 1032 cm^−1^ were assigned to excess AgOTf. Finally, adding formic acid produced a formate ion characteristic peak at ∼1728 cm^−1^, compared to 1730 cm^−1^ for pure HCOOH.

Carbon dioxide exhibits significant Raman activity due to polarizability changes (while the molecular dipole moment remains unchanged) during symmetric stretching vibrations. In situ Raman spectroscopy characterized CO_2_ peaks in Ag‐containing and Ag‐free systems (Figure 3c). The Ag‐free system showed almost no discernible CO_2_ peaks, whereas the AgNO_3_‐added system exhibited strong peaks near 1272 and 1383 cm^−1^ (ν1), corresponding to CO_2_ symmetric stretching [33, 34]. Meanwhile, in the Raman spectrum, we also observed the characteristic peaks of H_2_ at 355, 587, 814, 1034 cm^−1^ but almost no peaks in the Ag‐free system. This confirms that AgNO_3_ significantly promotes formic acid dehydrogenation, consistent with the high TOF values observed compared to Pd‐only systems. No CO characteristic peak at 2138 cm^−1^ was detected.

Water has been proposed to act as a proton shuttle in FA dehydrogenation, particularly in Ir‐tetracoordinated systems [35]. To investigate its influence on catalytic rate, we conducted experiments varying water content while keeping other conditions constant. As shown in Figure 3d, the dehydrogenation rate exhibited a non‐monotonic trend: adding a small amount of water (e.g., [Pd]/1 equiv.) increased the TOF slightly to 169.1 h^−1^ compared to the anhydrous system. Further increasing water to [Pd]/400 equiv. boosted the TOF to 597.4 h^−1^. However, exceeding [Pd]/800 equiv. led to a decline, with the TOF dropping to 357.4 h^−1^ at [Pd]/2000 equiv. This suggests that an appropriate amount of water enhances activity by facilitating silver salt dissolution, formic acid dissociation, and reactant coordination, while excess water may disrupt reaction equilibrium or active site structure.

### 2.1 Computational Studies

**FIGURE 4:**
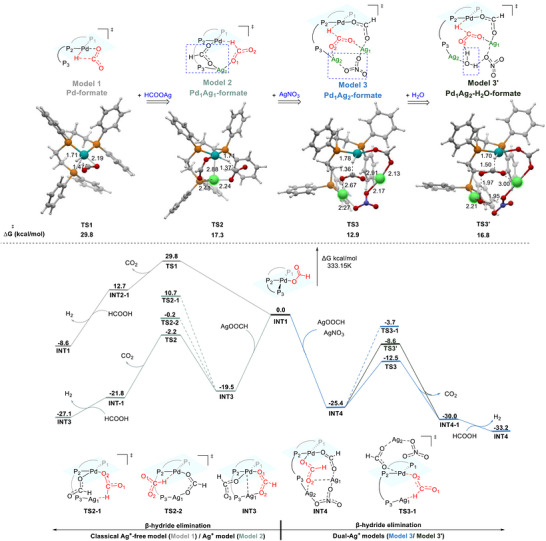
Energy profiles for β‐H elimination in different computational models. Each model corresponds to its respective transition state (TS), with key parameters labeled in the 3D structure at the upper part of the figure.

The structure of active species in the Pd_1_‐Ag_2_/**L2** system was further investigated via theoretical studies. To understand the impact of silver nitrate on formic acid dehydrogenation efficiency, DFT mechanistic calculations were performed at the SMD(1,4‐dioxane)‐B3LYP/6‐311++G(d,p)//B3LYP/SDD(Pd,Ag)/6‐31G(d) level [36, 37, 38], incorporating SMD solvation corrections [39, 40, 41, 42]. As shown in Figure 4, we constructed a series of transition state models in a stepwise manner following a model‐incremental strategy. We built a classical Ag^+^‐free model (**Model 1**/**TS1**) and progressively assessed Ag^+^ synergistic effects in **Model 2**/**TS2** and **Model 3**/**TS3**. Based on the dual‐Ag^+^ model, H_2_O effects were also investigated (**Model 3′**/**TS3′**). A novel eight‐membered cyclic elimination model is proposed in the transition states, involving Ag synergism through Pd‐H agostic interactions, which is distinct from the classical four‐membered β‐H elimination. Additionally, formate and nitrate counterions play important roles in stabilizing bridged Pd‐Ag_2_ and Ag_2_‐Ag_1_ interactions.

For the tridentate phosphine ligand **L2**, two phosphorus atoms coordinate with Pd, and the third with Ag^+^. This arrangement facilitates dehydrogenation primarily through β‐H elimination driven by Pd‐H agostic interactions (**TS2**, **TS3**), whereas Ag‐H elimination is comparatively unfavorable (**TS2‐1**, **TS3‐1**). The energy surface is shown in Figure 4. Ligand exchange among palladium acetate, tridentate phosphine, and formate leads to exothermic formation of the formic acid palladium species **INT1** (Figure S23), initiating subsequent reactions at zero‐point energy. For the classical model (**Model 1**, Figure S24), a four‐membered cyclic transition state (**TS1**) was observed. Here, departing CO_2_ is not stabilized by Ag^+^, resulting in a high‐energy barrier (29.8 kcal/mol) that renders the reaction unfavorable. In **Model 2**, including one Ag^+^ and a counteracting formate anion (more structures, see Figure S25), the tridentate phosphine bridges Pd^2+^ and Ag^+^, establishing Pd‐Ag synergistic interactions that stabilize **TS2**. This new eight‐membered β‐H elimination transition state is governed by Pd‐H and Ag─O═C interactions, reducing the barrier to 17.3 kcal/mol. Conversely, β‐H elimination via Pd‐H agostic interactions without Ag─O═C involvement (**TS2‐2**) increases the barrier by 2 kcal/mol, underscoring silver ion's stabilizing role.

Furthermore, the kinetic barrier for the Pd─O═C carbonyl and Ag‐H model via **TS2‐1** escalates to 30.2 kcal/mol, emphasizing the important β‐H elimination of Pd‐H agostic interactions rather than the Ag‐H one. Comparing with **Model 2**, **Model 3** was built for the role of the second Ag^+^ ion and nitrate counterion. This Ag^+^ stabilizes the carbonyl oxygen of nascent CO_2_. The nitrate anion bridges the two Ag^+^, cooperatively stabilizing the two carbonyl oxygen atoms of departing CO_2_ (Ag_1_‐O_1_: 2.91 Å; Ag_2_‐O_2_: 2.67 Å). Transition state **TS3** in this model retains the eight‐membered cyclic structure with Pd‐H agostic interactions, similar to **Model 2**, while **Model 3** incorporates an additional eight‐membered bridged structure including nitrate, pre‐dissociating CO_2_, and two Ag^+^, enhancing transition state stability and reducing the barrier to 12.9 kcal/mol. In contrast, Ag‐H agostic interactions in **TS3‐1** represent an energetically unfavorable pathway (Figure S26). Considering dual‐Ag^+^ cooperative stabilization, we further explored H_2_O effects in **Model 3'**. A stable eight‐membered bridged structure was identified, featuring two hydrogen bonds of water (1.97 Å, 1.95 Å) with nitrate and carbonyl oxygen instead of the Ag^+^ coordination. The overall activation barrier (ΔG‡ = 16.8 kcal/mol), while slightly higher than **Model 3**, suggests that water may also inhibit the reactivity through breaking Ag^+^‐carbonyl stabilization, in agreement with the experimental observation in Figure 3d.

### 2.2 ESI‐MS Analysis

**FIGURE 5:**
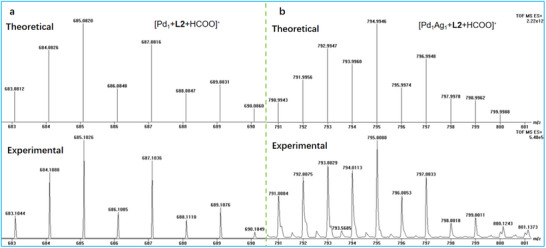
ESI‐MS spectra of intermediates observed during FA dehydrogenation in dioxane/water after 1 h. Experimental and theoretical peaks for (a) **Pd**+**L2+HCOO** (**Model 1**); and (b) Pd_1_Ag_1_+**L2**+HCOO ([**Model 2**‐HCOO]^+^).

In addition to computational studies, electrospray ionization mass spectrometry (ESI‐MS) was also conducted to identify key intermediates during the reaction. We confirmed the existence of several crucial intermediates by analyzing mass‐to‐charge ratios (m/z) of the detected ions in the mass spectra and comparing them with the theoretical values. For instance, an intermediate corresponding to **Model 1** exhibited a theoretical m/z = 685.0820, consistent with the experimental value of m/z = 685.1026 (Δ < 20.6 mDa and 30.1 ppm) (Figure 5a). Furthermore, specie Pd+**L2**+(HCOO)(CH_3_COO)_2_ with a chemical formula as [C_39_H_40_O_6_P_3_Pd]^+^ was detected with m/z = 804.1302, consistent with that of a theoretical m/z = 804.1166 (Δ < 13.6 mDa and 16.9 ppm) (Figure S19); and Pd+**L2**+(HCOO)_3_ ([C_37_H_36_O_6_P_3_Pd]^+^) has an experimental m/z = 776.0924, agreeing with its theoretical m/z = 776.0852 (Δ < 7.2 mDa and 9.2 ppm) (Figure S20). These results confirm the identity of **Model 1** intermediates and **Model 1**‐related ions based on their m/z values and characteristic isotopic patterns.

Additionally, another ESI‐MS signal at m/z = 795.0080 corresponding to [**Model 2**‐HCOO]^+^ (Pd+Ag+**L2**+HCOO; [C_35_H_34_AgO_2_P_3_Pd]^+^; theoretical m/z = 794.9946; Δ < 16.8 mDa and 21.1 ppm) was observed (Figure 5b). This detection aligns with the predicted instability of one formate group in the DFT‐optimized **Model 2**. Further DFT‐based structural analysis of **Model 2** revealed that the bond cleavage energy barrier for the Pd‐O bond in its relatively unstable conformation correlated well with the ESI‐MS observations. The synergistic integration of experimental and theoretical analyses not only confirmed the existence of these intermediates but also elucidated the structure‐energy relationships governing their evolution and reaction barriers, providing dual validation for the mechanistic interpretation.

Based on these experimental and theoretical findings, we propose the following catalytic mechanism for the Pd_1_‐Ag_2_/**L2** system in formic acid dehydrogenation: (1) Formic acid coordinates to the Pd center of the Pd_1_‐Ag_2_/**L2** active site; (2) Ag atoms interact with the formate group via Ag─O═C coordination, weakening the C─H bond; (3) C─H bond cleavage generates a Pd‐H species and an Ag‐coordinated formate anion; (4) β‐H elimination between the Pd‐H species and the adjacent carbon‐bound hydrogen releases H_2_; (5) the regenerated Pd_1_‐Ag_2_/**L2** site re‐enters the catalytic cycle.

**FIGURE 6:**
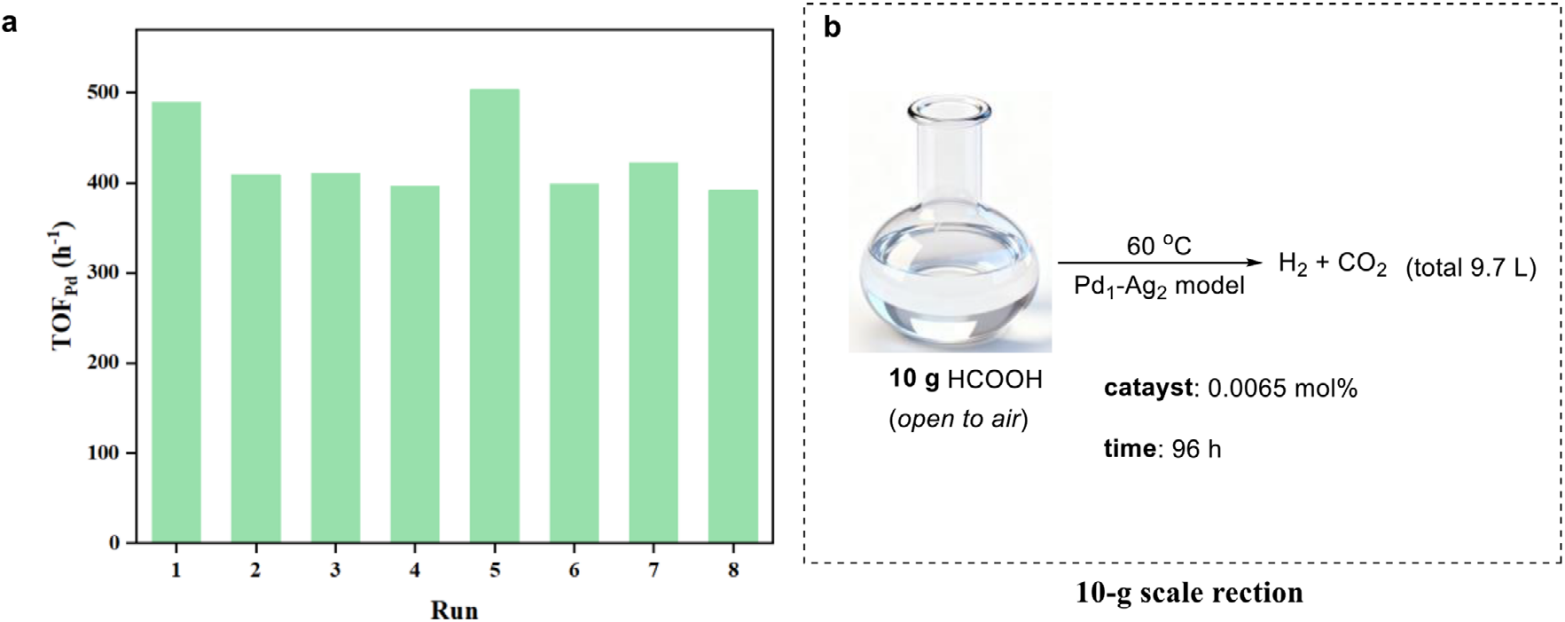
Applicability test: (a) Recycling experiments under air. Reaction conditions: 11.67 mmol FA, 2 mL solvent (1,4‐dioxane:H_2_O = 1:1), 0.12 mol% Pd(OAc)_2_, 0.12 mol% linear‐triphos (**L2**), and 0.24 mol% additive, 60°C; TOF is obtained for the 30 min period of the reaction. (b) 10 g scale reaction.

At last, the applicability of this Pd_1_‐Ag_2_/**L2** system was tested under practical conditions to validate its potential for scalable hydrogen production. Notably, the catalyst maintained high performance in ambient air without any protective atmosphere, achieving 8 consecutive recycling runs with negligible loss of activity (Figure 6a). Even after 8 cycles, the TOF remained at 388.5 h^−1^, and the total turnover number (TON) reached 10,500, far exceeding the durability of most reported homogeneous Pd‐based systems, which typically deactivate within the first cycle under air. This air stability eliminates the need for inert gas protection, a critical advantage for industrial‐scale operations.

To further assess scalability, we performed a large‐scale dehydrogenation experiment using 10.0 g of formic acid as the substrate, with catalyst loading kept consistent with small‐scale tests (20 µmol Pd(OAc)_2_, 20 µmol **L2**, 40 µmol AgNO_3_) in a dioxane/water mixed solvent (Figure 6b). The reaction proceeded smoothly at 60°C under ambient air, with complete conversion of formic acid to H_2_ and CO_2_ within 96 h, as confirmed by GC analysis. No CO formation was detected throughout the process, and the gas evolution rate remained stable (average 50.5 mL H_2_ per hour), demonstrating the system's ability to handle 10 gram‐scale substrates without sacrificing efficiency.

For practical applications, the Pd_1_‐Ag_2_ catalyst was compared with some commercial metal catalysts for formic acid dehydrogenation (Table S11). Conventional homogeneous catalysts generally suffer from toxic CO generation, rapid deactivation, and unsatisfactory air stability. In comparison, this bimetallic Pd‐Ag catalyst effectively reduces the usage of noble metals and improves economic efficiency. These results highlight the key practical merits of the Pd_1_‐Ag_2_ bimetallic system: favorable durability, complete CO suppression, remarkable air tolerance, and reliable recyclability, which shows distinct competitiveness against conventional commercial materials. Benefiting from the above features, it is highly applicable to portable hydrogen production and distributed fuel cell devices, which are urgently needed for realistic hydrogen energy utilization.

## 3 Conclusion

In summary, we have developed a molecular Pd_1_Ag_2_ model that enables efficient, base‐free FA dehydrogenation by overcoming the β‐H elimination kinetic barrier. The addition of silver nitrate to the Pd/**L2** complex significantly boosts the efficiency, with the turnover frequency (TOF) increasing from 62.3 to 542.4 h^−1^ in air. Furthermore, this new system can be recycled up to eight times without apparent loss of activity (TON up to 10,500). DFT calculations reveal that dual Ag^+^ ions and nitrate anions stabilize an eight‐membered transition state, reducing the β‐H elimination barrier from 29.8 to 12.9 kcal/mol. This work highlights bimetallic synergy via Ag electronic modulation under ligand geometric protection as a strategy to address both kinetics and stability, guiding the design of next‐generation dehydrogenation catalysts.

## Author Contributions

**Xiaoyang Yan**: writing – original draft, data curation, formal analysis, investigation, methodology. **Hongdan Zhu**: funding acquisition, investigation. **Qian Peng**: conceptualization, methodology, funding acquisition, writing – review and editing, project administration. **Xiaolin Jiang**: conceptualization, writing – original draft, funding acquisition, methodology, resources, writing – review and editing. **Mingdong Zhou**: conceptualization, methodology, writing – review and editing, supervision. **Shenglong Jiang**: investigation, data curation, formal analysis. **Xiaochun Zhou**: conceptualization. **Heng Wang**: funding acquisition, methodology. **Xiaopeng Li**: conceptualization, methodology, resources.

## Conflicts of Interest

The authors declare no conflict of interest.

## Supporting information




**Supporting File**: advs76028‐sup‐0001‐SuppMat.docx.

## Data Availability

The data that support the findings of this study are available from the corresponding author upon reasonable request.
