# Supplementary material for: Adaptive Coordination Engineering for Efficient and Robust H2 Release
Source: Adv Sci (Weinh). 2026 Jun 11:e76028. Online ahead of print. doi: 10.1002/advs.76028 (PMC13336577; doi:10.1002/advs.76028)
Supplement: Supplementary file 1 — Supporting File: advs76028‐sup‐0001‐SuppMat.docx. [file ADVS-9999-e76028-s001.docx]

***Supporting Information***

**Adaptive Coordination Engineering for Efficient and Robust H_2_ Release**

*Xiaolin Jiang,^1,#,^* Xiaoyang Yan^2,#^, Shenglong Jiang^3,#^, Hongdan Zhu^2^, Heng Wang^4^, Xiaopeng Li^4^, Xiaochun Zhou^5^, Mingdong Zhou^3,^* and Qian Peng^2,^**

X. Jiang, X. Yan and S. Jiang contributed equally to this work.

X. Jiang

^1^Digital and Intelligent Empowerment Biomedical Innovation Center, School of Pharmacy, Shanghai University of Medicine and Health Sciences, Shanghai 201318, P.R. China

E-mail: spujxl@163.com

X. Yan, H. Zhu, Q. Peng

^2^State Key Laboratory of Elemento-organic Chemistry, Tianjin Key Laboratory of Biosensing and Molecular Recognition College of Chemistry, Frontiers Science Center for New Organic Matter, Nankai University, Tianjin 300072, P.R. China

E-mail: qpeng@nankai.edu.cn

S. Jiang, M. Zhou

^3^College of Chemical Engineering, Shenyang University of Chemical Technology, Shenyang 110142, P.R. China

E-mail: mingdongzhou@syuct.edu.cn

1. Wang, X. Li

^4^State Key Laboratory of Organometallic Chemistry, Shanghai Institute of Organic Chemistry, Chinese Academy of Sciences (CAS), Shanghai 200032, P.R. China

X. Zhou

^5^Division of Advanced Nanomaterials, Suzhou Institute of Nano-tech and Nano-bionics, Chinese Academy of Sciences (CAS), Suzhou 215125, China

**1. General information**

Unless otherwise stated, all the materials were purchased from commercial suppliers (Adamas, TCI) and were used as received. ^1^H NMR spectra were obtained at room temperature on a Bruker 400 NMR spectrometer operating at 400 MHz (^1^H). ^1^H NMR chemical shifts (*δ*) are reported in ppm and were referenced internally to residual solvent resonances (DMSO‑*d*_6_: *δ*_H_ = 2.49 ppm, CDCl_3_: *δ*_H_ = 7.26 ppm, DCM-*d_2_*: *δ*_H_ = 5.32 ppm). ^31^P NMR chemical shifts are reported in parts per million downfield from H_3_PO_4_ and referenced to an external 85% solution of phosphoric acid. Data are reported as follows: chemical shift, multiplicity (s = singlet, d = doublet, t = triplet, m = multiplet, br. = broad singlet), coupling constants (Hz) and integration. GC data were obtained GC-G5 (Beijing Persee General Instrument CO. Ltd). Infrared spectra were recorded in the range 4000-400 cm^-1^ on a Bruker Alpha FT-IR spectrometer. UV-visible (UV-Vis) spectra of the complexes were recorded on a Thermo Scientific Evolution 60 spectrophotometer in a 1 cm-path length quartz cells. Raman spectra were recorded on a Renishaw; INV/A0524-17. Electrospray ionization-high-resolution mass spectra (ESI-HRMS) were collected on a Waters Synapt G2-Si mass spectrometer (Waters Corp., Milford, MA, USA). Cyclic voltammograms were obtained using a CHI 602D (CH Instruments Co., USA) electrochemical analyzer. The electrochemical workstation was equipped with a three electrode system, namely, glassy carbon, platinum wire and Ag/AgCl as the working, auxiliary and reference electrodes, respectively. All measurements were carried out at room temperature unless otherwise stated.

**2.** **Reaction procedures**

**2.1. General procedure for the reactions**

In a typical run, metal salt precursor, ligand and solvent were added into the reactor directly and the reactor was preheated for 10 min at test temperature. Afterward, HCOOH with certain volume was transferred into this reactor. The concentration of the HCOOH, base, metal salt precursor, ligand and solvent were adjusted to achieve the best hydrogen production efficiency.

**2.2. General test procedure for the reactions**

The measurement of hydrogen and carbon dioxide and the set-up of the experimental procedures are according to previous work.^[1]^ After the reaction has finished, the gas phase of all the experiments have been analysed by introducing a syringe through the GC valve. And the amount of H_2_ and CO_2_ were detected with semi liquid filled U-shaped tube and GC-G5 (Beijing Persee General Instrument CO. Ltd). The gas component of the reaction can be detected with gas chromatography by TCD detector.

CO can be detected with gas chromatography by FID detector with methane conversion reactor. The GC trace showing that the CO level is below the GC detection limit of 10 ppm (time-range, 0 to 7 min). Blue curves represent the standard CO gas samples, green curves represent the reaction samples. Sample is the generated gas mixture during the dehydrogenation of FA at 70 ^o^C after two hours of reaction. (Reaction conditions: 11.67 mmol HCOOH, Pd(OAc)_2_ (0.12 mol%), AgNO_3_ (0.24 mol%), linear triphos **L2** (0.12 mol%), dioxane (1 mL), H_2_O (1 mL), 60 ^o^C). And as shown in **Figure S1**, there is no CO in the gas products.


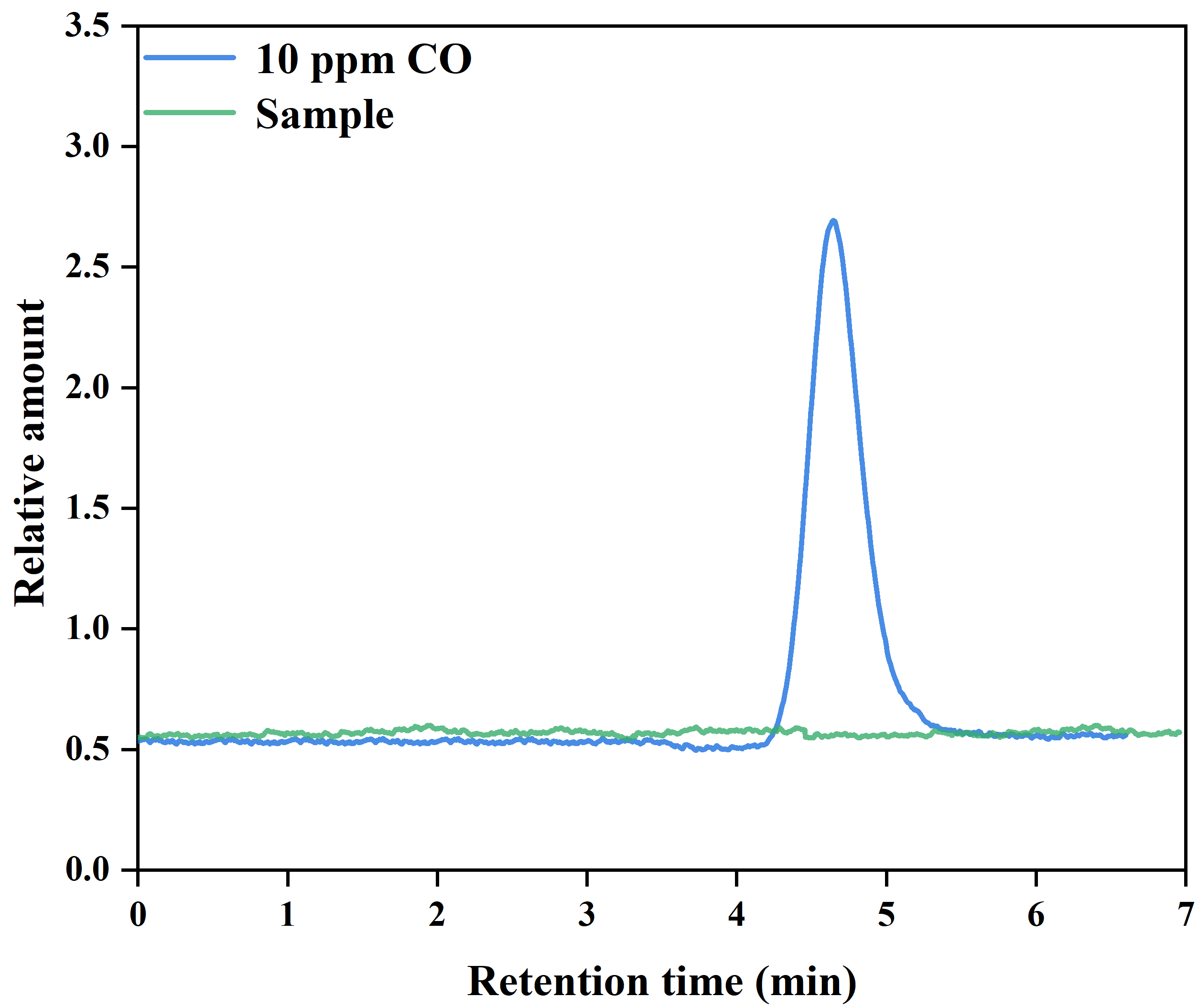


**Figure S1.** CO measurement by gas chromatography.

**3. Formic acid dehydrogenation set-up**

**
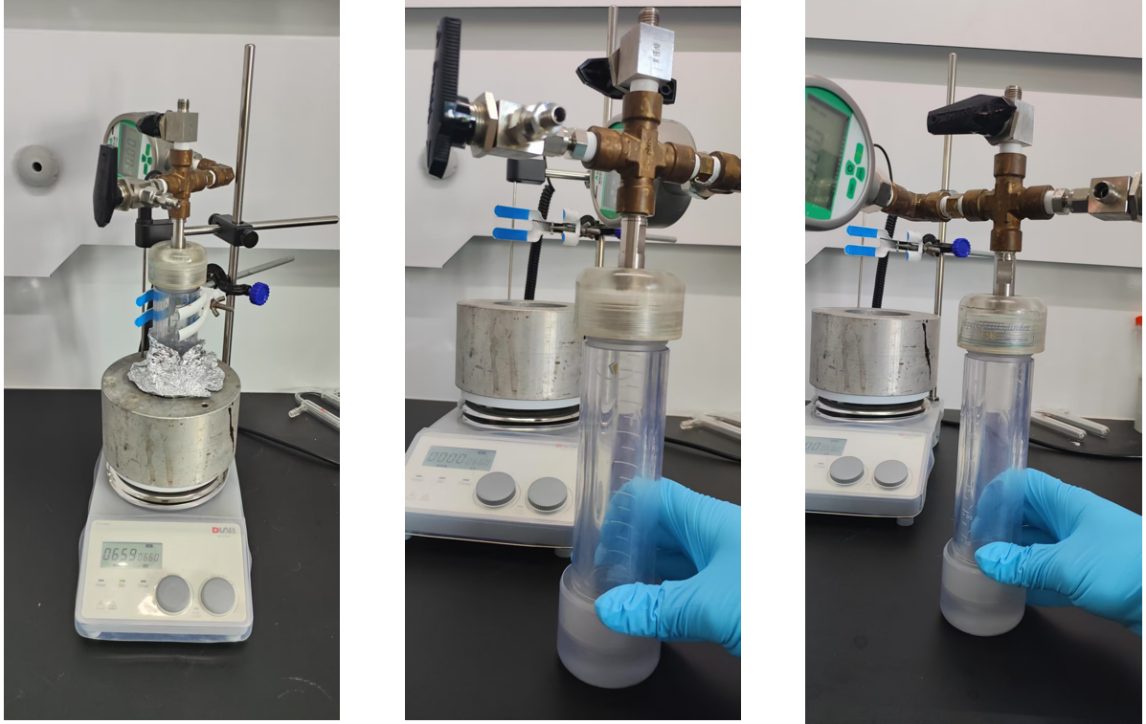
**

**
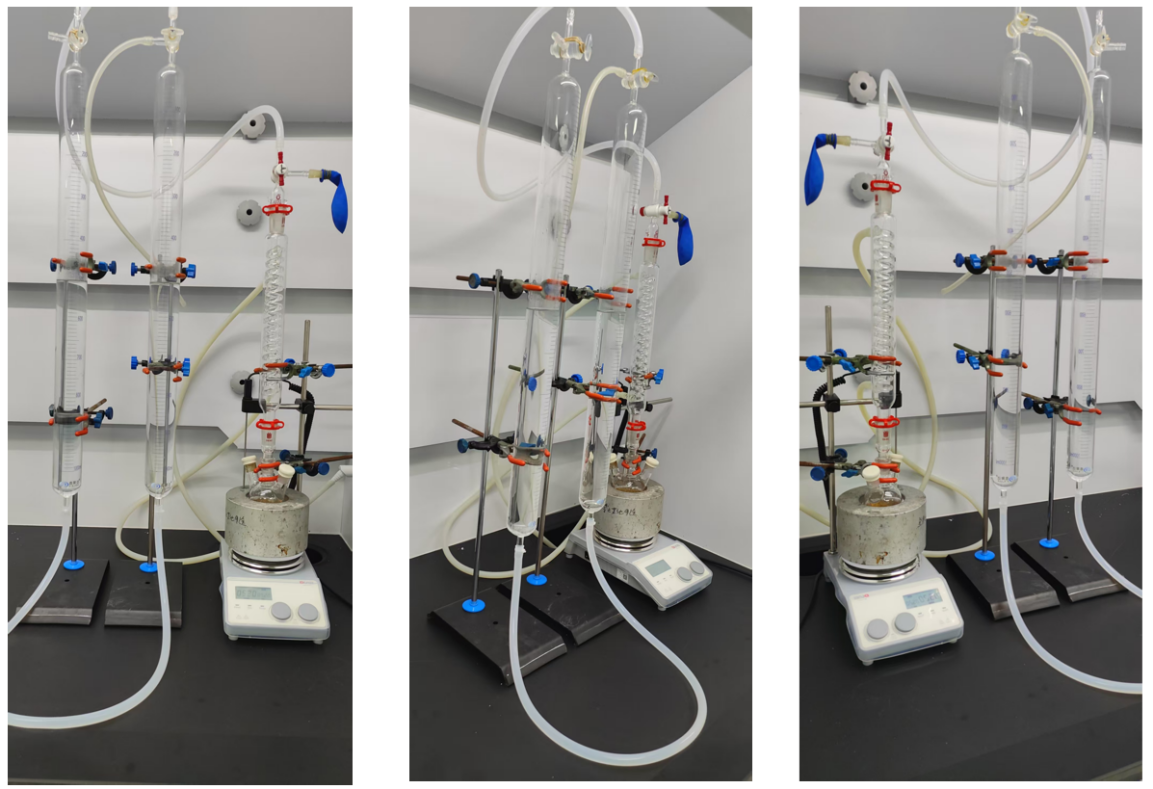
**

**Figure S2**. Image of the reaction setup.

**4. Calculation of TON and TOF**

The calculation of molar volumes can be calculated from the Van der Waals equation as follows:

$$\text{V}_{\text{m}\text{(}\text{H}\text{2)}\text{, 25 ℃}}\text{=}\frac{\text{RT}}{\text{P}}\text{+}\text{b}\text{−}\frac{\text{a}}{\text{RT}}\text{=24.}\text{49}\frac{\text{L}}{\text{mol}}$$

R: 8.3145 m^3^·Pa·mol^-1^·K^-1^;

T: 273.15 + room temperature (^o^C) = 298.15 K;

p: 101325 Pa;

a: 2.49×10^-10^ Pa·m^3^·mol^-2^;

b: 26.7×10^-6^ m^3^·mol^-1^.

The calculation of molar volumes can be calculated from the Van der Waals equation as follows:

$$\text{V}_{\text{m}\text{(}\text{CO}\text{2)}\text{, 25 ℃}}\text{=}\frac{\text{RT}}{\text{P}}\text{+}\text{b}\text{−}\frac{\text{a}}{\text{RT}}\text{=24.}\text{41}\frac{\text{L}}{\text{mol}}$$

R: 8.3145 m^3^·Pa·mol^-1^·K^-1^;

T: 273.15 + room temperature (^o^C) = 298.15 K;

p: 101325 Pa;

a: 3.65×10^-10^ Pa·m^3^·mol^-2^;

b: 42.7×10^-6^ m^3^·mol^-1^.

Gas evolution was corrected with the blank volume considering 1:1 ratio of H_2_:CO_2_. The blank volume corresponds to the gas evolution of the same reaction without catalyst. The turnover frequency (TOF) value was calculated using the following equation:

$$\text{TOF}\text{=}\frac{\text{V}_{\text{increase}}\text{/(}\text{V}_{\text{m}\text{,}\text{ }\text{H}\text{2}\text{ 25 ℃}}\text{+}\text{V}_{\text{m}\text{,}\text{ }\text{CO}\text{2}\text{ 25 ℃}}\text{)}}{{\text{t}\text{×}\text{n}}_{\text{catalyst}}}$$

$$\text{V}_{\text{increase}}\text{=}\text{V}_{\text{recorded}}\text{−}\text{V}_{\text{blank}}$$

$$\text{V}_{\text{recorded}}\text{=}\text{V}_{\text{t}}\text{⋅}\left[ \text{H}_{\text{2}} \right]_{\text{t}}\text{＋}\text{V}_{\text{t}}\text{⋅}\left[ \text{CO}_{\text{2}} \right]_{\text{t}}$$

The turnover number (TON) value was calculated using the following equation:

$$\text{TON}\text{=}\text{TOF}\text{×}\text{t}$$

V_increase_ was the increase of gas volume, V_recorded_ and V_blank_ are the gas volume at measurement time and initial time, [H_2_]_t_ is the concentration of hydrogen at measurement time and initial time, [CO_2_]_t_ is the concentration of carbon dioxide at measurement time and initial time, n_catalyst_ was the total mole number of [Pd] in reaction, t was the period for measurement.

V_recorded_: volume measured in the catalytic reaction.

V_blank_: volume measured in the catalytic reaction without catalyst.

Vm_(H2)_, 25 ^o^C and Vm_(CO2)_, 25 ^o^C: molar volumes of H_2_ and CO_2_ respectively calculated with the Van Der Waals equation.

**5. Conditions optimization for the reaction of dehydrogenation of formic acid**

**Table S1.** Investigation of different kinds of [Pd]-catalyzed dehydrogenation of formic acid at 60 ^o^C.

| Entry | [Pd] | Ligand | TOF (h^-1^) |
| --- | --- | --- | --- |
| 1 | PdCl_2_ | - | 4.2 |
| 2 | Pd(OAc)_2_ | - | 11.7 |
| 3 | Pd(acac)_2_ | - | 6.5 |

Reaction conditions: 11.67 mmol FA, 6.8 mg HCOONa, 0.12 mmol% [Pd], 1 mL 1,4-dioxane and 1 mL H_2_O, 60 ^o^C; TOF (h^-1^) is obtained for the most active 20 min period of the reaction.

**Table S2.** Investigation of different kinds of ligands catalyzed dehydrogenation of formic acid at 60 ^o^C.

| Entry | Ligand | TOF (h^-1^) | Entry | Ligand | TOF (h^-1^) |
| --- | --- | --- | --- | --- | --- |
| 1 | 5-NO_2_-1,10-phenanthroline | 14.1 | 6 | DPPF | 27.8 |
| 2 | 5-NH_2_-1,10-phenanthroline | 12.7 | 7 | 1,1'-bis(diisopropylphosphino)ferrocene | 1.7 |
| 3 | 2,2'-bipyridine | 17.4 | 8 | tris(2-carboxyethyl)phosphine Hydrochloride | 0.4 |
| 4 | α,α,α-tripyridine | 18.2 | 9 | S-Phos | 46.1 |
| 5 | TPPTS | 0.9 | 10 | linear-triphos (L2) | 88.4 |

Reaction conditions: 11.67 mmol FA, 6.8 mg HCOONa, 0.12 mmol% [Pd] and ligand, 1 mL 1,4-dioxane and 1 mL H_2_O, 60 ^o^C; TOF (h^-1^) is obtained for the most active 20 min period of the reaction.

**Table S3.** Investigation of different times and phosphine ligands of Pd(OAc)_2_-catalyzed dehydrogenation of formic acid at 60 ^o^C.

| Entry | [Pd] | Time (min) | TOF (h^-1^) | Entry | [Pd] | Time (min) | TOF (h^-1^) |
| --- | --- | --- | --- | --- | --- | --- | --- |
| 1 | PPh_3_ (**L1**) | 20 | 58.9 | 10 | S-phos | 20 | 46.1 |
| 2 | PPh_3_ (**L1**) | 40 | 37.8 | 11 | S-phos | 40 | 48.3 |
| 3 | PPh_3_ (**L1**) | 60 | 33.1 | 12 | S-phos | 60 | 39.2 |
| 4 | DPPF | 20 | 27.8 | 13 | xantphos | 20 | 38.5 |
| 5 | DPPF | 40 | 23.7 | 14 | xantphos | 40 | 59.2 |
| 6 | DPPF | 60 | 17.4 | 15 | xantphos | 60 | 50.4 |
| 7 | tripodal-triphos (**L3**) | 20 | 32.6 | 16 | DPPE | 20 | 17.3 |
| 8 | tripodal-triphos (**L3**) | 40 | 25.2 | 17 | DPPE | 40 | 17.3 |
| 9 | tripodal-triphos (**L3**) | 60 | 24.1 | 18 | DPPE | 60 | 16.1 |

Reaction conditions: 11.67 mmol FA, 6.8 mg HCOONa, 0.12 mmol% Pd(OAc)_2_ and ligand, 1 mL 1,4-dioxane and 1 mL H_2_O, 60 ^o^C; TOF (h^-1^) is obtained for the different time of the reaction.

**Table S4.** Investigation of different times of [Pd]-**L2** catalyzed dehydrogenation of formic acid at 60 ^o^C.

| Entry | [Pd] | Time (min) | TOF (h^-1^) | Entry | [Pd] | Time (min) | TOF (h^-1^) |
| --- | --- | --- | --- | --- | --- | --- | --- |
| 1 | Pd(OAc)_2_ | 20 | 88.4 | 7 | Pd(acac)_2_ | 20 | 60.0 |
| 2 | Pd(OAc)_2_ | 40 | 108.0 | 8 | Pd(acac)_2_ | 40 | 69.1 |
| 3 | Pd(OAc)_2_ | 60 | 107.1 | 9 | Pd(acac)_2_ | 60 | 86.5 |
| 4 | Pd(OAc)_2_ | 80 | 82.3 | 10 | Pd(acac)_2_ | 80 | 79.5 |
| 5 | Pd(OAc)_2_ | 100 | 70.6 | 11 | Pd(acac)_2_ | 100 | 64.2 |
| 6 | Pd(OAc)_2_ | 120 | 68.3 | 12 | Pd(acac)_2_ | 120 | 55.7 |

Reaction conditions: 11.67 mmol FA, 6.8 mg HCOONa, 0.12 mmol% [Pd] and **L2**, 1 mL 1,4-dioxane and 1 mL H_2_O, 60 ^o^C; TOF (h^-1^) is obtained for the different time of the reaction.

**Table S5.** Investigation of different [M]-**L2** catalyzed dehydrogenation of formic acid at 60 ^o^C.

| Entry | [M] | TOF (h^-1^) | Entry | [M] | TOF (h^-1^) |
| --- | --- | --- | --- | --- | --- |
| 1 | Zn(OAc)_2_ | 0 | 5 | AgOAc | 0.1 |
| 2 | Cu(OAc)_2_ | 0 | 6 | Fe(OAc)_2_ | 0.1 |
| 3 | Co(OAc)_2_ | 0 | 7 | Mn(OAc)_2_ | 0.1 |
| 4 | Ni(OAc)_2_ | 38.6 | 8 | Pd(OAc)_2_ | 88.4 |

Reaction conditions: 11.67 mmol FA, 6.8 mg HCOONa, 0.12 mmol% [M] and **L2**, 1 mL 1,4-dioxane and 1 mL H_2_O, 60 ^o^C; TOF (h^-1^) is obtained for the most active 20 min period of the reaction.

**Table S6.** Investigation of Pd(OAc)_2_-**L2** catalyzed dehydrogenation of formic acid in air at 60 ^o^C.

| Entry | Pd(OAc)_2_ | Ligand | Atmosphere | Time (min) | TOF (h^-1^) |
| --- | --- | --- | --- | --- | --- |
| 1 | Pd(OAc)_2_ | **L2** | air | 20 | 68.7 |
| 2 | Pd(OAc)_2_ | **L2** | air | 40 | 101.2 |
| 3 | Pd(OAc)_2_ | **L2** | air | 60 | 101.6 |
| 4 | Pd(OAc)_2_ | **L2** | air | 80 | 94.2 |
| 5 | Pd(OAc)_2_ | **L2** | air | 100 | 82.6 |
| 6 | Pd(OAc)_2_ | **L2** | air | 120 | 73.4 |

*^a^*Reaction conditions: 11.67 mmol FA, 6.8 mg HCOONa, 0.12 mmol% Pd(OAc)_2_ and **L2**, 1 mL 1,4-dioxane and 1 mL H_2_O, in air, 60 ^o^C; TOF (h^-1^) is obtained for the different time of the reaction.

**Table S7.** Investigation of without HCOONa catalyzed dehydrogenation of formic acid in air at 60 ^o^C.

| Entry | X mol% | Time (min) | TOF (h^-1^) |
| --- | --- | --- | --- |
| 1 | 0.12 | 20 | 62.3 |
| 2 | 0.12 | 40 | 100.6 |
| 3 | 0.12 | 60 | 90.8 |
| 4 | 0.12 | 80 | 96.1 |
| 5 | 0.12 | 100 | 76.3 |
| 6 | 0.12 | 120 | 71.7 |

*^a^*Reaction conditions: 11.67 mmol FA, 0.12 mmol% Pd(OAc)_2_ and 0.12 mmol% **L2**, 1 mL 1,4-dioxane and 1 mL H_2_O, in air, 60 ^o^C; TOF (h^-1^) is obtained for the different time of the reaction.

**Table S8.** Investigation of different additives catalyzed dehydrogenation of formic acid in air at 60 ^o^C .

| Entry | Additive | Time (min) | TOF (h^-1^) | Entry | Additive | Time (min) | TOF (h^-1^) |
| --- | --- | --- | --- | --- | --- | --- | --- |
| 1 | Na_2_S_2_O_8_ | 20 | 28.8 | 37 | AgBF_4_ | 20 | 254.2 |
| 2 | Na_2_S_2_O_8_ | 40 | 29.8 | 38 | AgBF_4_ | 40 | 189.1 |
| 3 | Na_2_S_2_O_8_ | 60 | 48.5 | 39 | AgBF_4_ | 60 | 171.9 |
| 4 | Na_2_S_2_O_8_ | 80 | 53.4 | 40 | AgBF_4_ | 80 | 151.2 |
| 5 | Na_2_S_2_O_8_ | 100 | 55.5 | 41 | AgBF_4_ | 100 | 146.8 |
| 6 | Na_2_S_2_O_8_ | 120 | 76.8 | 42 | AgBF_4_ | 120 | 128.3 |
| 7 | ZrCl_4_ | 20 | 5.4 | 43 | Ag_2_SO_4_ | 20 | 89.1 |
| 8 | ZrCl_4_ | 40 | 12.0 | 44 | Ag_2_SO_4_ | 40 | 141.4 |
| 9 | ZrCl_4_ | 60 | 9.9 | 45 | Ag_2_SO_4_ | 60 | 132.3 |
| 10 | ZrCl_4_ | 80 | 12.7 | 46 | Ag_2_SO_4_ | 80 | 129.5 |
| 11 | ZrCl_4_ | 100 | 14.7 | 47 | Ag_2_SO_4_ | 100 | 178.8 |
| 12 | ZrCl_4_ | 120 | 9.3 | 48 | Ag_2_SO_4_ | 120 | 149.7 |
| 13 | ZrF_4_ | 20 | 70.7 | 49 | AgTFA | 20 | 335.9 |
| 14 | ZrF_4_ | 40 | 62.8 | 50 | AgTFA | 40 | 240.2 |
| 15 | ZrF_4_ | 60 | 92.7 | 51 | AgTFA | 60 | 160.4 |
| 16 | ZrF_4_ | 80 | 82.8 | 52 | AgTFA | 80 | 164.5 |
| 17 | ZrF_4_ | 100 | 94.4 | 53 | AgTFA | 100 | 148.3 |
| 18 | ZrF_4_ | 120 | 109.0 | 54 | AgTFA | 120 | 131.8 |
| 19 | LiBF_4_ | 20 | 132.8 | 55 | AgOTs | 20 | 89.7 |
| 20 | LiBF_4_ | 40 | 177.6 | 56 | AgOTs | 40 | 91.2 |
| 21 | LiBF_4_ | 60 | 158.5 | 57 | AgOTs | 60 | 140.1 |
| 22 | LiBF_4_ | 80 | 131.6 | 58 | AgOTs | 80 | 196.2 |
| 23 | LiBF_4_ | 100 | 123.3 | 59 | AgOTs | 100 | 177.5 |
| 24 | LiBF_4_ | 120 | 117.6 | 60 | AgOTs | 120 | 173.9 |
| 25 | Zn(BF_4_)_2_ | 20 | 211.1 | 61 | AgOTf | 20 | 238.9 |
| 26 | Zn(BF_4_)_2_ | 40 | 203.6 | 62 | AgOTf | 40 | 215.7 |
| 27 | Zn(BF_4_)_2_ | 60 | 197.6 | 63 | AgOTf | 60 | 218.7 |
| 28 | Zn(BF_4_)_2_ | 80 | 192.6 | 64 | AgOTf | 80 | 159.3 |
| 29 | Zn(BF_4_)_2_ | 100 | 181.9 | 65 | AgOTf | 100 | 149.1 |
| 30 | Zn(BF_4_)_2_ | 120 | 166.6 | 66 | AgOTf | 120 | 145.9 |
| 31 | Zn(OAc)_2_ | 20 | 100.8 | 67 | AgNO_3_ | 20 | 533.7 |
| 32 | Zn(OAc)_2_ | 40 | 213.8 | 68 | AgNO_3_ | 40 | 489.1 |
| 33 | Zn(OAc)_2_ | 60 | 251.1 | 69 | AgNO_3_ | 60 | 453.3 |
| 34 | Zn(OAc)_2_ | 80 | 236.4 | 70 | AgNO_3_ | 80 | 372.9 |
| 35 | Zn(OAc)_2_ | 100 | 190.2 | 71 | AgNO_3_ | 100 | 313.5 |
| 36 | Zn(OAc)_2_ | 120 | 182.0 | 72 | AgNO_3_ | 120 | 288.9 |

Reaction conditions: 11.67 mmol FA, 0.12 mmol% Pd(OAc)_2_ and 0.12 mmol% **L2**, 0.24 mmol% different additive,1 mL 1,4-dioxane and 1 mL H_2_O, 60 ^o^C; TOF (h^-1^) is obtained for the different time of the reaction.

**Table S9.** Investigation of different additives contain NO_3_^-^ catalyzed dehydrogenation of formic acid in air at 60 ^o^C.

| Entry | Additive | Time (min) | TOF (h^-1^) | Entry | Additive | Time (min) | TOF (h^-1^) |
| --- | --- | --- | --- | --- | --- | --- | --- |
| 1 | Zn(NO_3_)_2_ | 20 | 90.3 | 19 | Yb(NO_3_)_3_ | 20 | 65.1 |
| 2 | Zn(NO_3_)_2_ | 40 | 80.0 | 20 | Yb(NO_3_)_3_ | 40 | 76.7 |
| 3 | Zn(NO_3_)_2_ | 60 | 71.4 | 21 | Yb(NO_3_)_3_ | 60 | 99.1 |
| 4 | Zn(NO_3_)_2_ | 80 | 60.2 | 22 | Yb(NO_3_)_3_ | 80 | 144.6 |
| 5 | Zn(NO_3_)_2_ | 100 | 53.6 | 23 | Yb(NO_3_)_3_ | 100 | 107.6 |
| 6 | Zn(NO_3_)_2_ | 120 | 47.3 | 24 | Yb(NO_3_)_3_ | 120 | 162.3 |
| 7 | Ni(NO_3_)_2_ | 20 | 72.2 | 25 | Ce(NO_3_)_3_ | 20 | 83.1 |
| 8 | Ni(NO_3_)_2_ | 40 | 69.1 | 26 | Ce(NO_3_)_3_ | 40 | 85.4 |
| 9 | Ni(NO_3_)_2_ | 60 | 47.7 | 27 | Ce(NO_3_)_3_ | 60 | 98.2 |
| 10 | Ni(NO_3_)_2_ | 80 | 33.5 | 28 | Ce(NO_3_)_3_ | 80 | 93.9 |
| 11 | Ni(NO_3_)_2_ | 100 | 36.5 | 29 | Ce(NO_3_)_3_ | 100 | 96.8 |
| 12 | Ni(NO_3_)_2_ | 120 | 27.7 | 30 | Ce(NO_3_)_3_ | 120 | 92.5 |
| 13 | Co(NO_3_)_2_ | 20 | 98.3 | 31 | Cu(NO_3_)_2_ | 20 | 74.6 |
| 14 | Co(NO_3_)_2_ | 40 | 99.4 | 32 | Cu(NO_3_)_2_ | 40 | 54.1 |
| 15 | Co(NO_3_)_2_ | 60 | 107.0 | 33 | Cu(NO_3_)_2_ | 60 | 53.7 |
| 16 | Co(NO_3_)_2_ | 80 | 110.2 | 34 | Cu(NO_3_)_2_ | 80 | 44.4 |
| 17 | Co(NO_3_)_2_ | 100 | 139.9 | 35 | Cu(NO_3_)_2_ | 100 | 44.3 |
| 18 | Co(NO_3_)_2_ | 120 | 104.1 | 36 | Cu(NO_3_)_2_ | 120 | 33.7 |

Reaction conditions: 11.67 mmol FA, 0.12 mmol% Pd(OAc)_2_ and 0.12 mmol% **L2**, 0.24 mmol% different additive, 1 mL 1,4-dioxane and 1 mL H_2_O, 60 ^o^C; TOF (h^-1^) is obtained for the different time of the reaction.

**Table S10.** Investigation of different solvents of Pd(OAc)_2_, AgNO_3_ and **L2** catalyzed dehydrogenation of formic acid in air at 60 ^o^C.

| Entry | Solvent | Time (min) | TOF (h^-1^) | Entry | Solvent | Time (min) | TOF (h^-1^) |
| --- | --- | --- | --- | --- | --- | --- | --- |
| 1 | 2 mLdioxane | 20 | 85.6 | 10 | 1-1 mLdioxane /H_2_O | 20 | 542.4 |
| 2 | 2 mLdioxane | 40 | 75.0 | 11 | 1-1 mLdioxane /H_2_O | 40 | 489.1 |
| 3 | 2 mLdioxane | 60 | 68.8 | 12 | 1-1 mLdioxane /H_2_O | 60 | 453.3 |
| 4 | 2 mL HCOOH | 20 | 38.8 | 13 | 1-1 mLTHF/H_2_O | 20 | 80.3 |
| 5 | 2 mL HCOOH | 40 | 28.0 | 14 | 1-1 mLTHF/H_2_O | 40 | 79.7 |
| 6 | 2 mL HCOOH | 60 | 27.1 | 15 | 1-1 mLTHF/H_2_O | 60 | 70.3 |
| 7 | 2 mL H_2_O | 20 | 86.2 | 16 | 1-1 mLEGDME/ H_2_O | 20 | 158.2 |
| 8 | 2 mL H_2_O | 40 | 77.2 | 17 | 1-1 mLEGDME/ H_2_O | 40 | 139.5 |
| 9 | 2 mL H_2_O | 60 | 67.4 | 18 | 1-1 mLEGDME/ H_2_O | 60 | 127.4 |

Reaction conditions: 11.67 mmol FA, 6.8 mg HCOONa, 0.12 mmol% Pd(OAc)_2_, 0.24 mmol% AgNO_3_, 0.12 mmol% **L2**, 1 mL 1,4-dioxane and 1 mL H_2_O, 60 ^o^C; TOF (h^-1^) is obtained for the different time of the reaction. THF = tetrahydrofuran. EGDME = ethylene glycol dimethyl ether.

**Table S11.** Performance comparison between Pd_1_-Ag_2_ bimetallic catalyst and commercial catalysts.

| Catalyst | Solution | Base | Air sensibility | CO | T/^o^C | TOF(h^-1^) |
| --- | --- | --- | --- | --- | --- | --- |
| 1 | H_2_O | HCOONa | Air-tolerated | Not mentioned | 120 | 670^[2]^ |
| 2 | 1,4-dioxane/H_2_O | HCOONa | Not mentioned | No CO | 69 | 286^[3]^ |
| 3 | H_2_O | NaHCO_3_ | Not mentioned | Not mentioned | 50 | 130^[4]^ |
| 4 | DMF | NEt_3_ | Not mentioned | Not mentioned | 60 | 200^[5]^ |
| 5 | THF | NEt_3_ | Not mentioned | No CO | *hv*/40 | 503^[6]^ |
| Pd_1_-Ag_2_ | 1,4-dioxane/H_2_O | No base | Air-tolerated | No CO | 60 | 542  (This work) |

**6. Video for the reaction of dehydrogenation of formic acid in different conditions**

1.
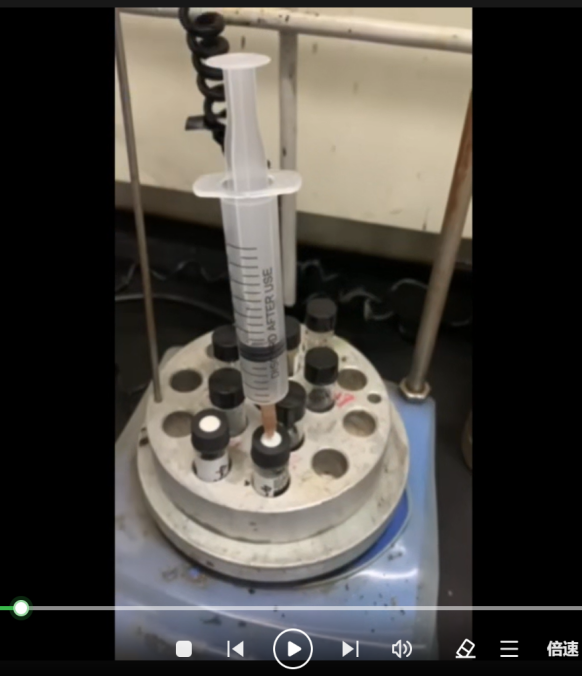
(b)
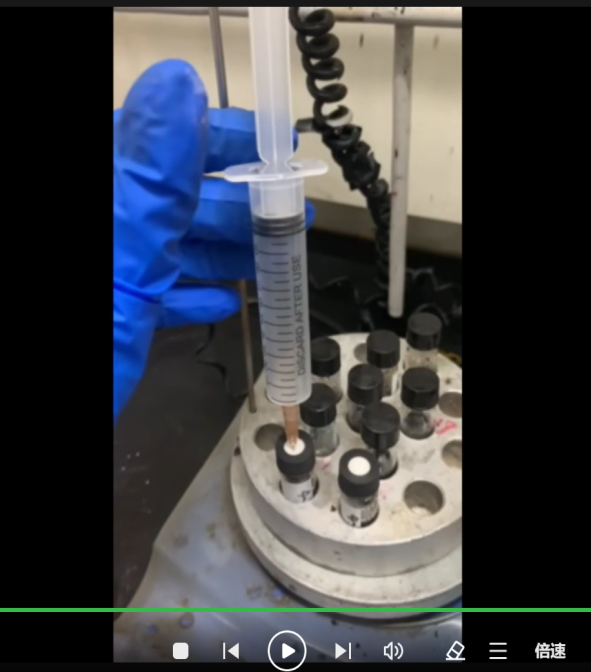


**Figure S3.** Video and image for the reaction of dehydrogenation of formic acid in different conditions. Reaction conditions: (a) reaction of just Pd(OAc)_2_ and **L2** in 0.5 mins; (b) reaction upon addition of AgNO_3_ to the mixture of Pd(OAc)_2_ and **L2** in 0.5 mins.

**7. Mechanistic experiments**

**7.1. Copies of reaction NMR**

^
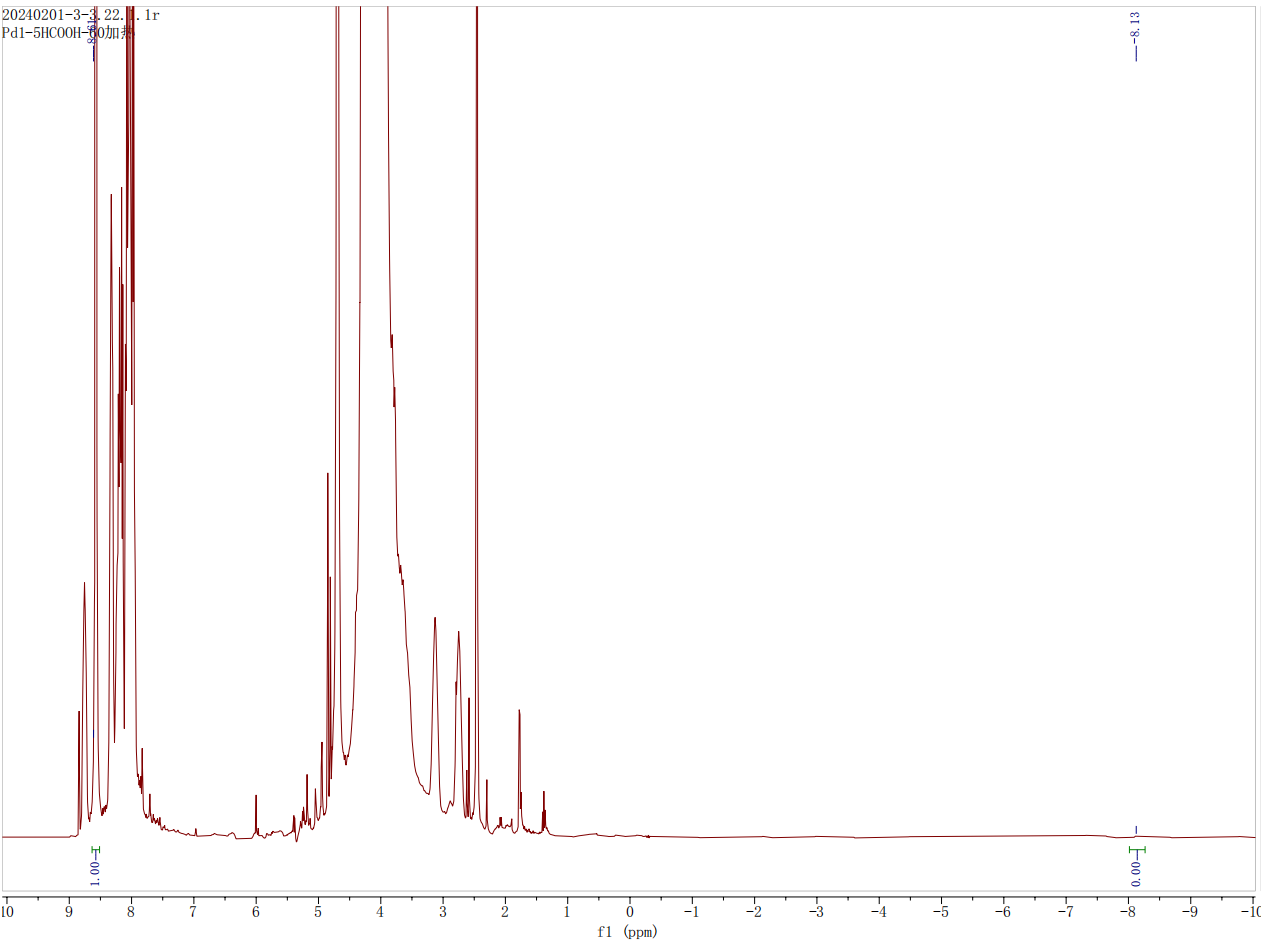
^

**Figure S4.** ^1^H NMR for Pd(OAc)_2_ +5 equiv. HCOOH

^
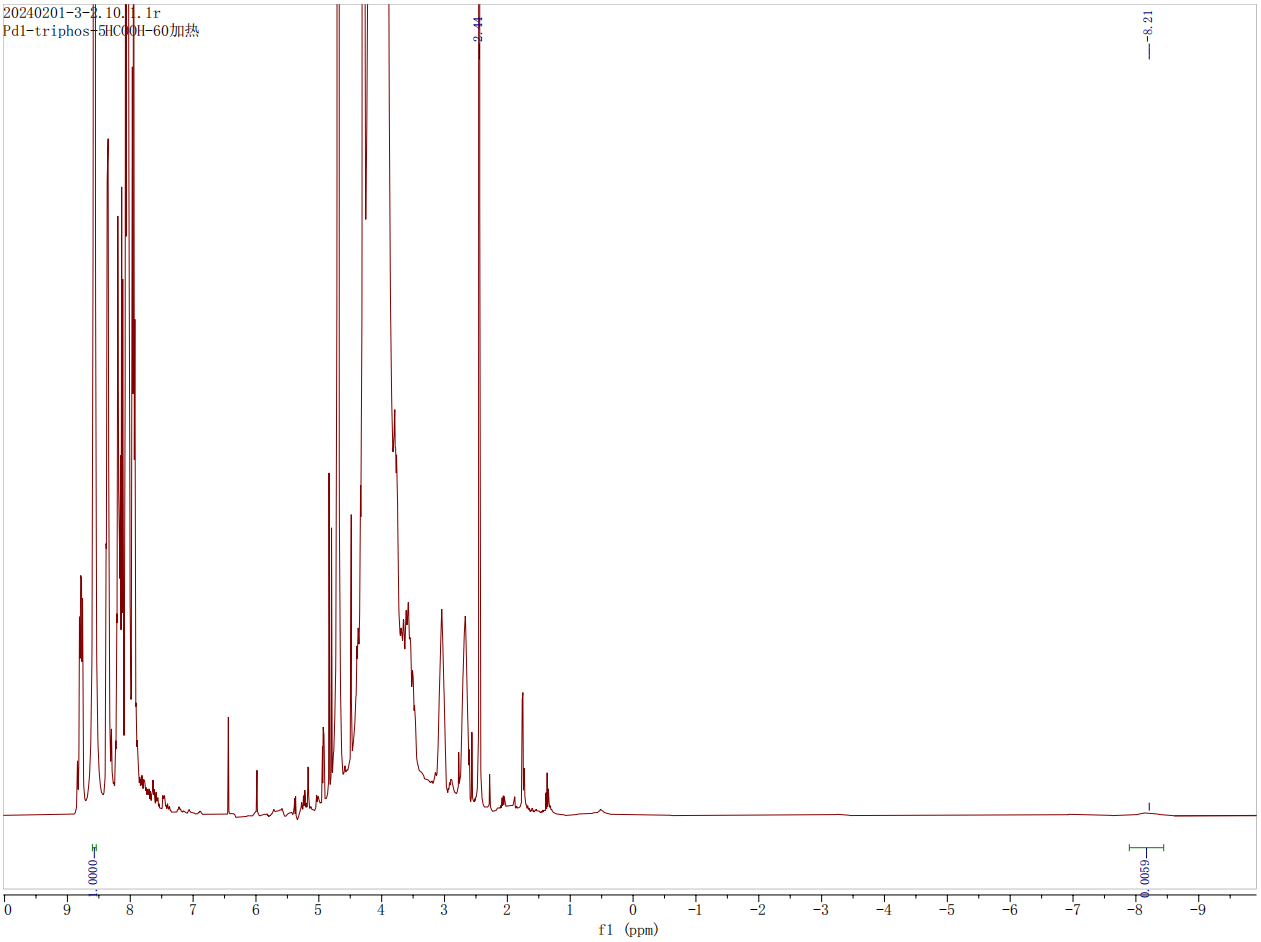
^

**Figure S5.** ^1^H NMR for Pd(OAc)_2_ + **L2** +5 equiv. HCOOH

^
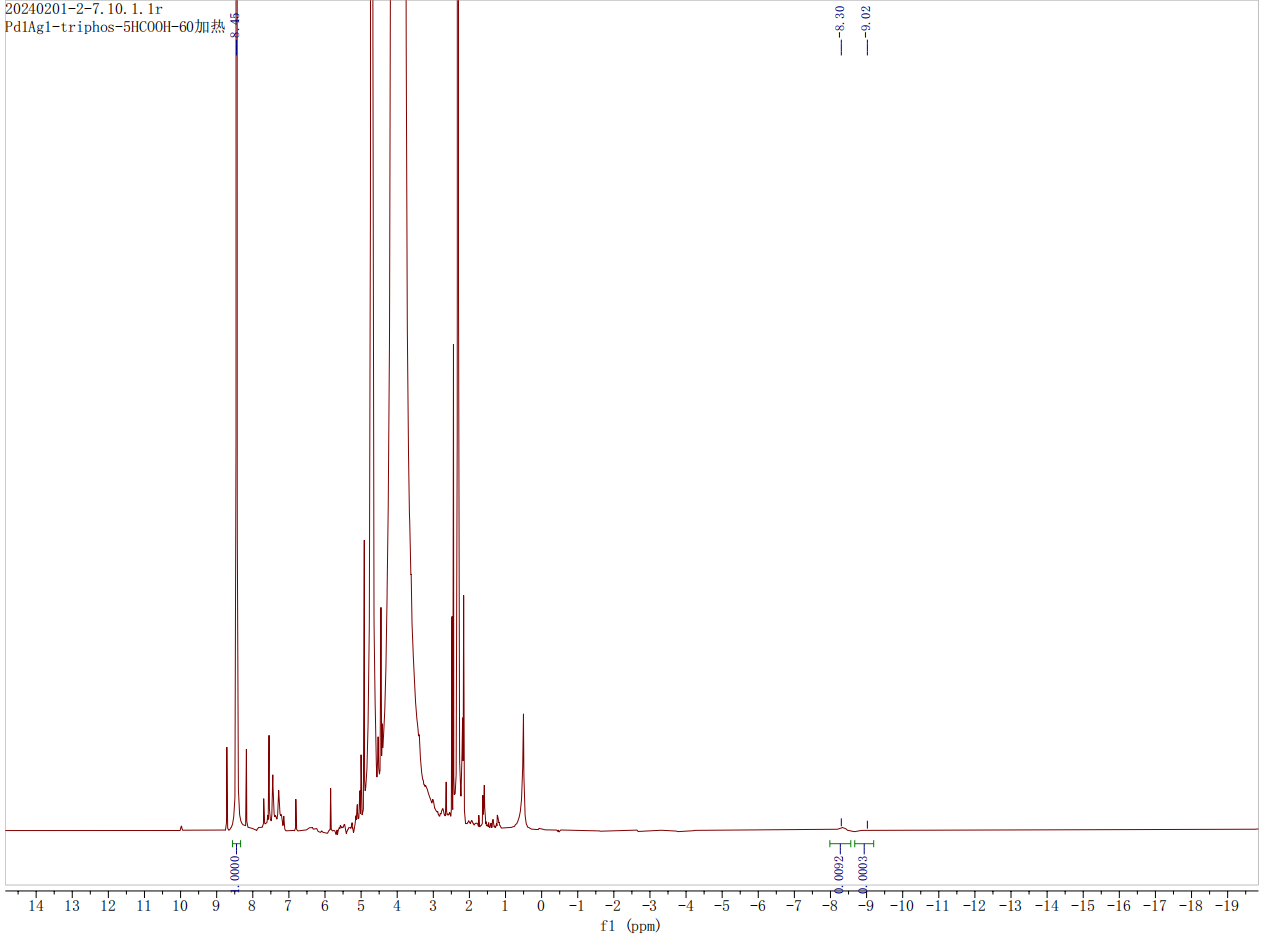
^

**Figure S6.** ^1^H NMR for Pd(OAc)_2_ + 1 equiv. AgNO_3_ + **L2** + 5 equiv. HCOOH


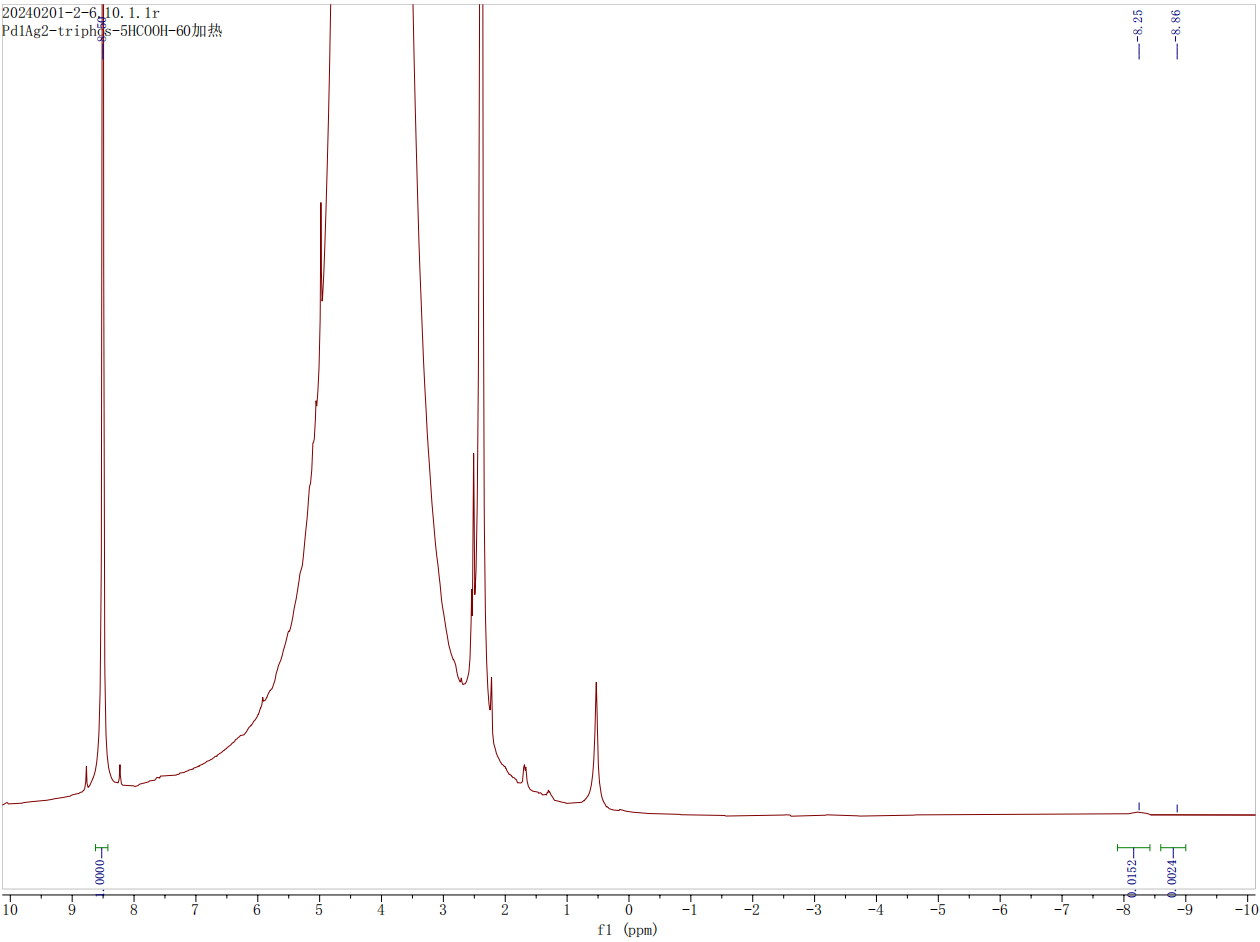


**Figure S7.** ^1^H NMR for Pd(OAc)_2_ + 2 equiv. AgNO_3_ + **L2** + 5 equiv. HCOOH


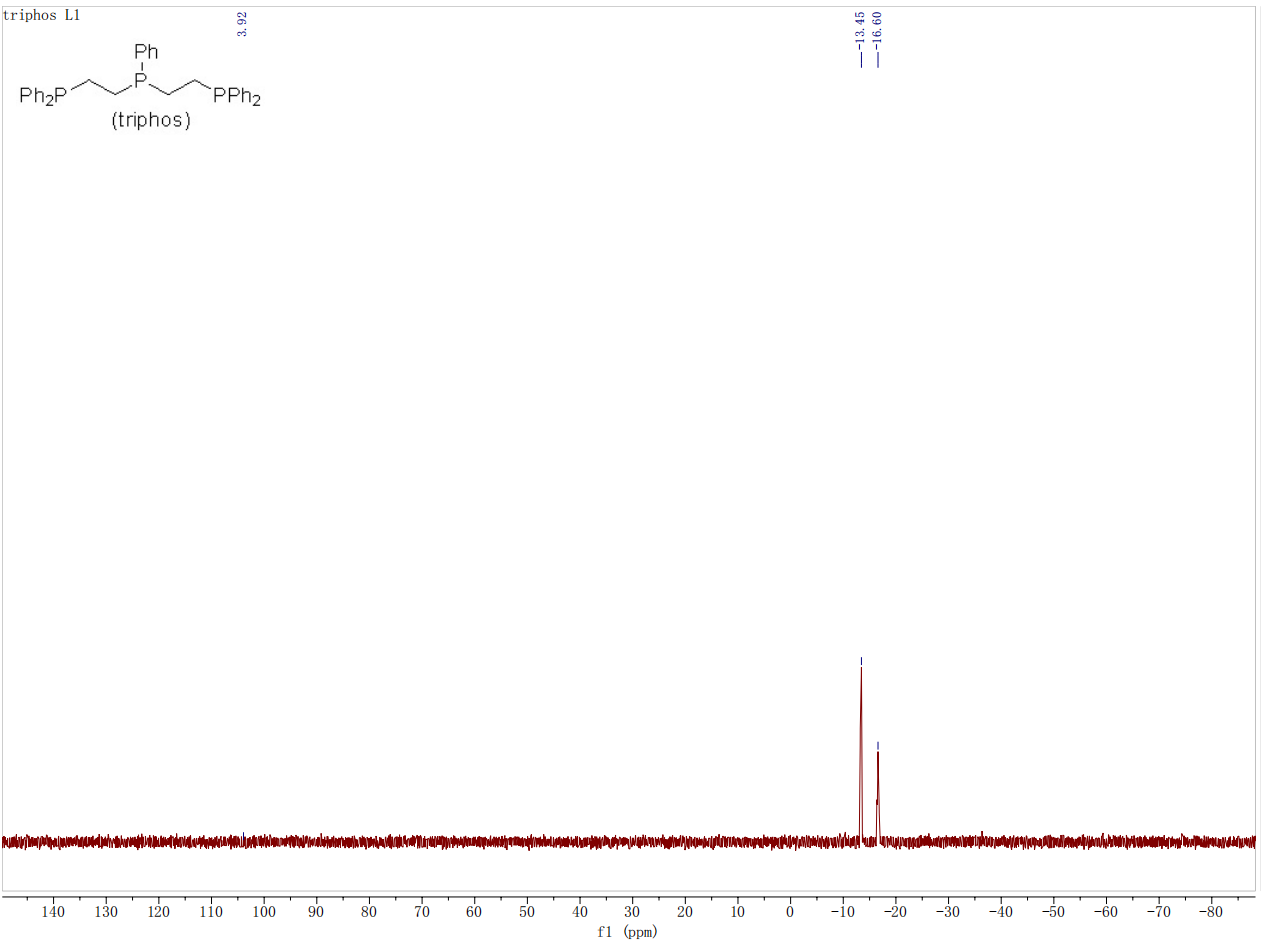


**Figure S8.** ^31^P NMR for **L2**


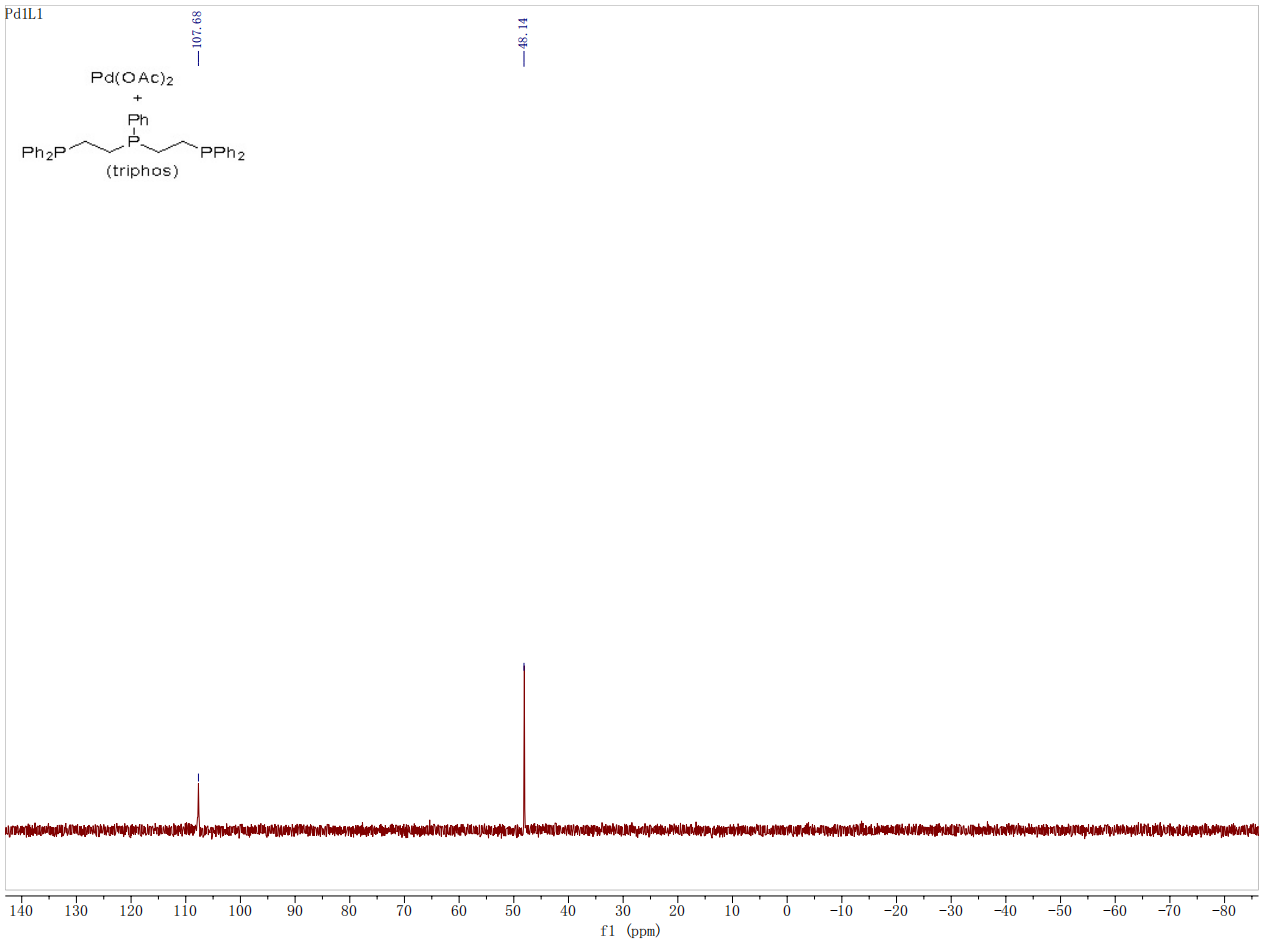


**Figure S9.** ^31^P NMR for Pd(OAc)_2_ + **L2**


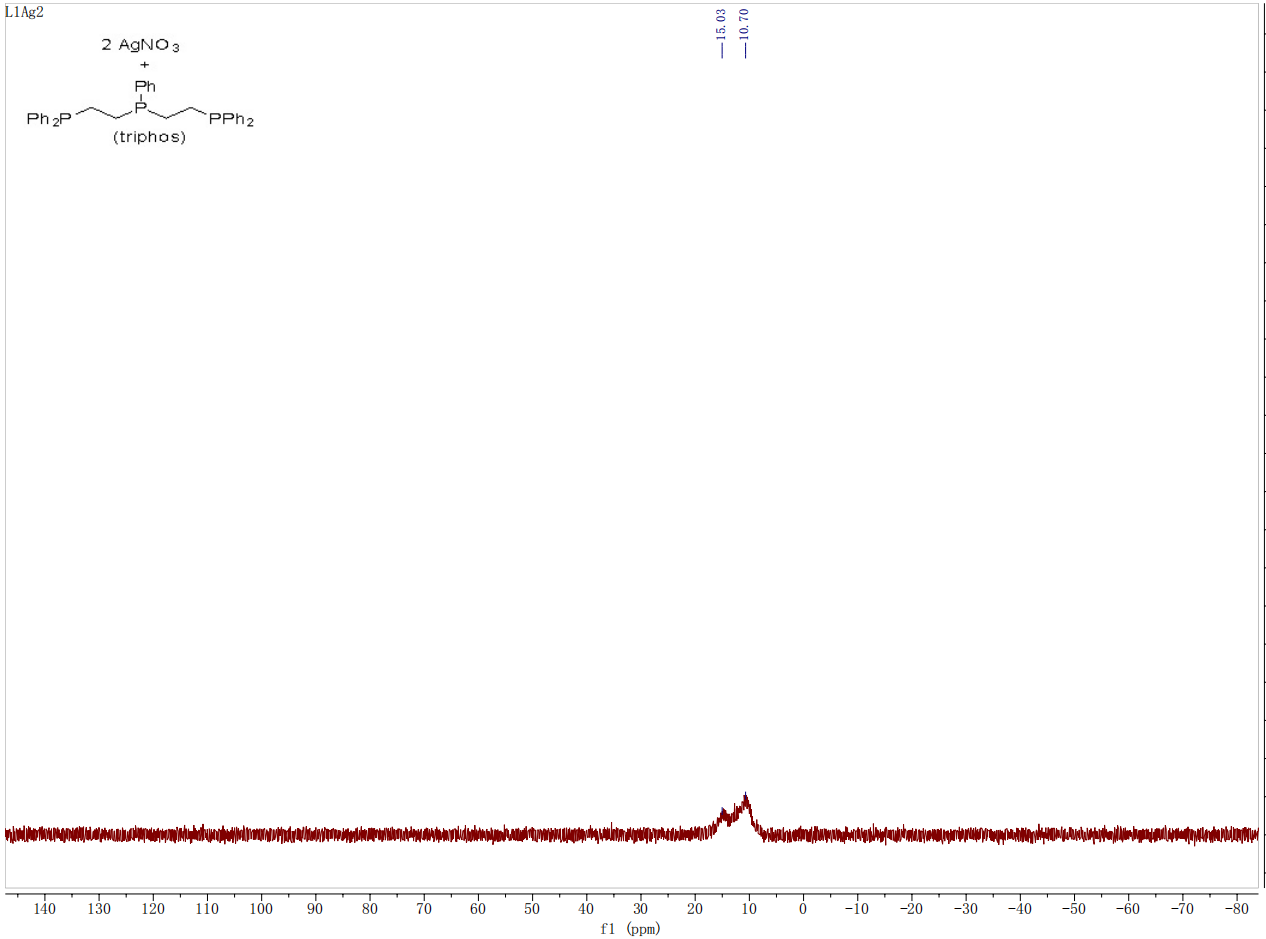


**Figure S10.** ^31^P NMR for **L2** + 2 equiv. AgNO_3_


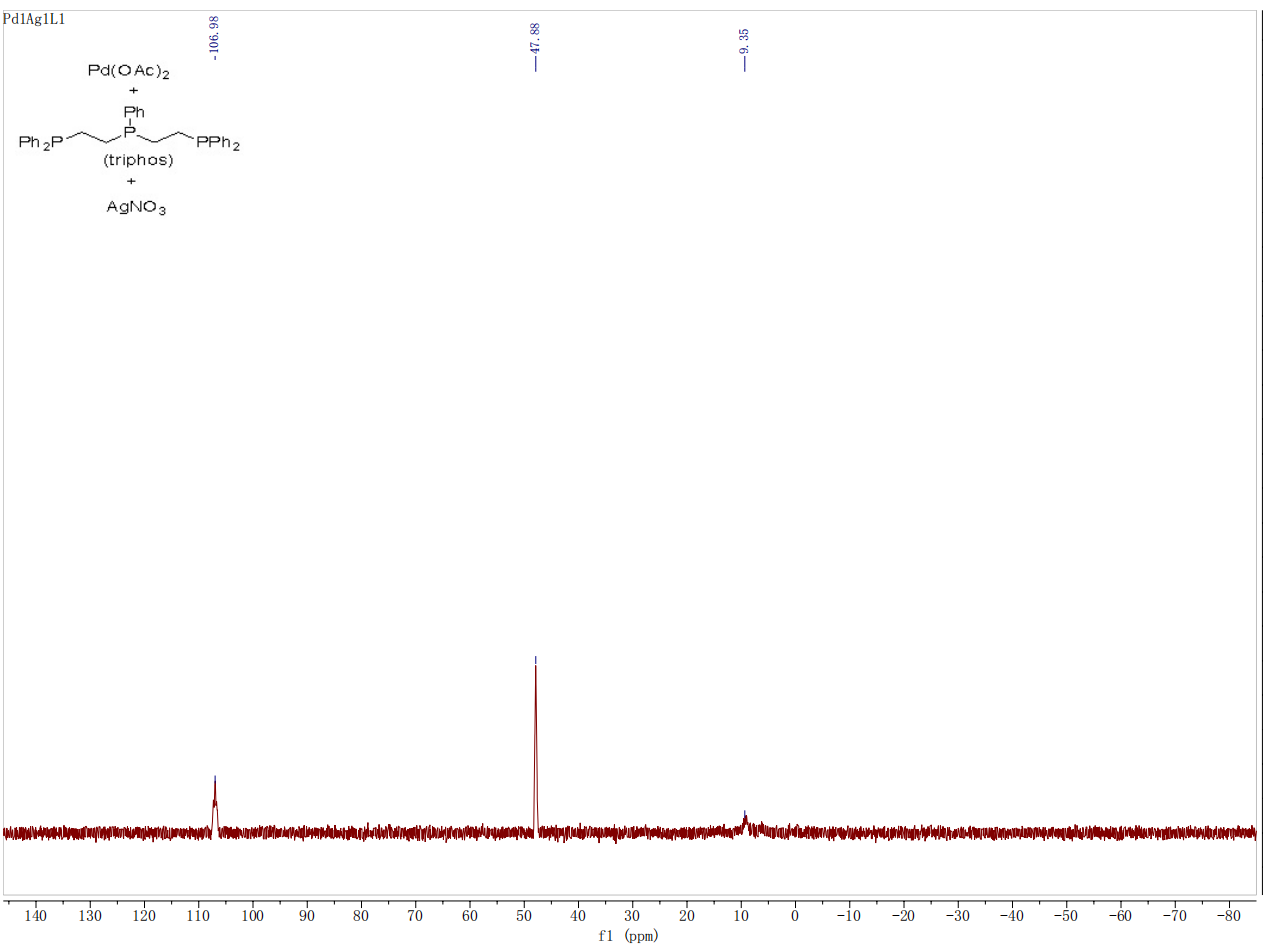


**Figure S11.** ^31^P NMR for Pd(OAc)_2_+ 1 equiv. AgNO_3_ + **L2**


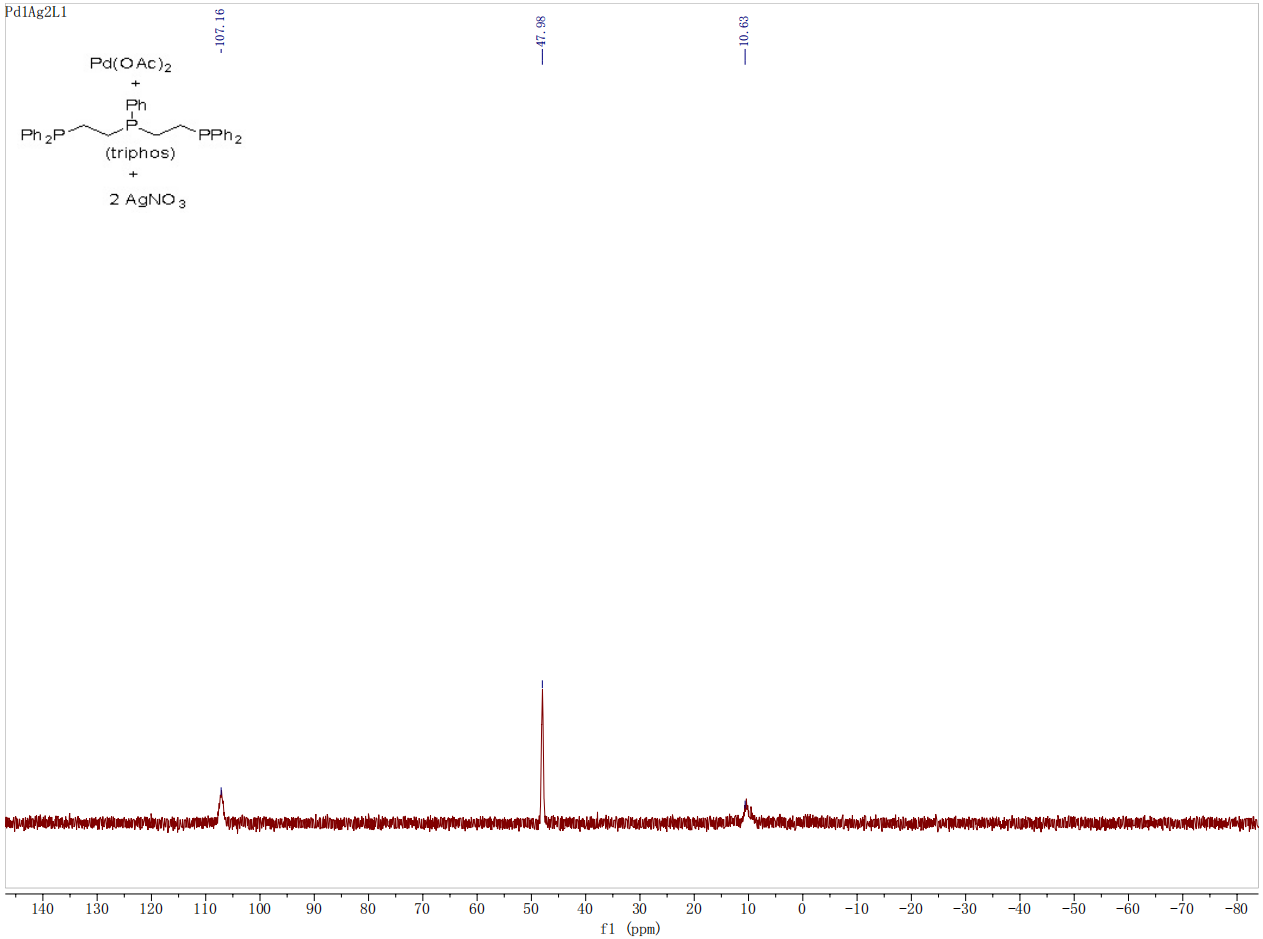


**Figure S12.** ^31^P NMR for Pd(OAc)_2_ +2 equiv. AgNO_3_ + **L2**


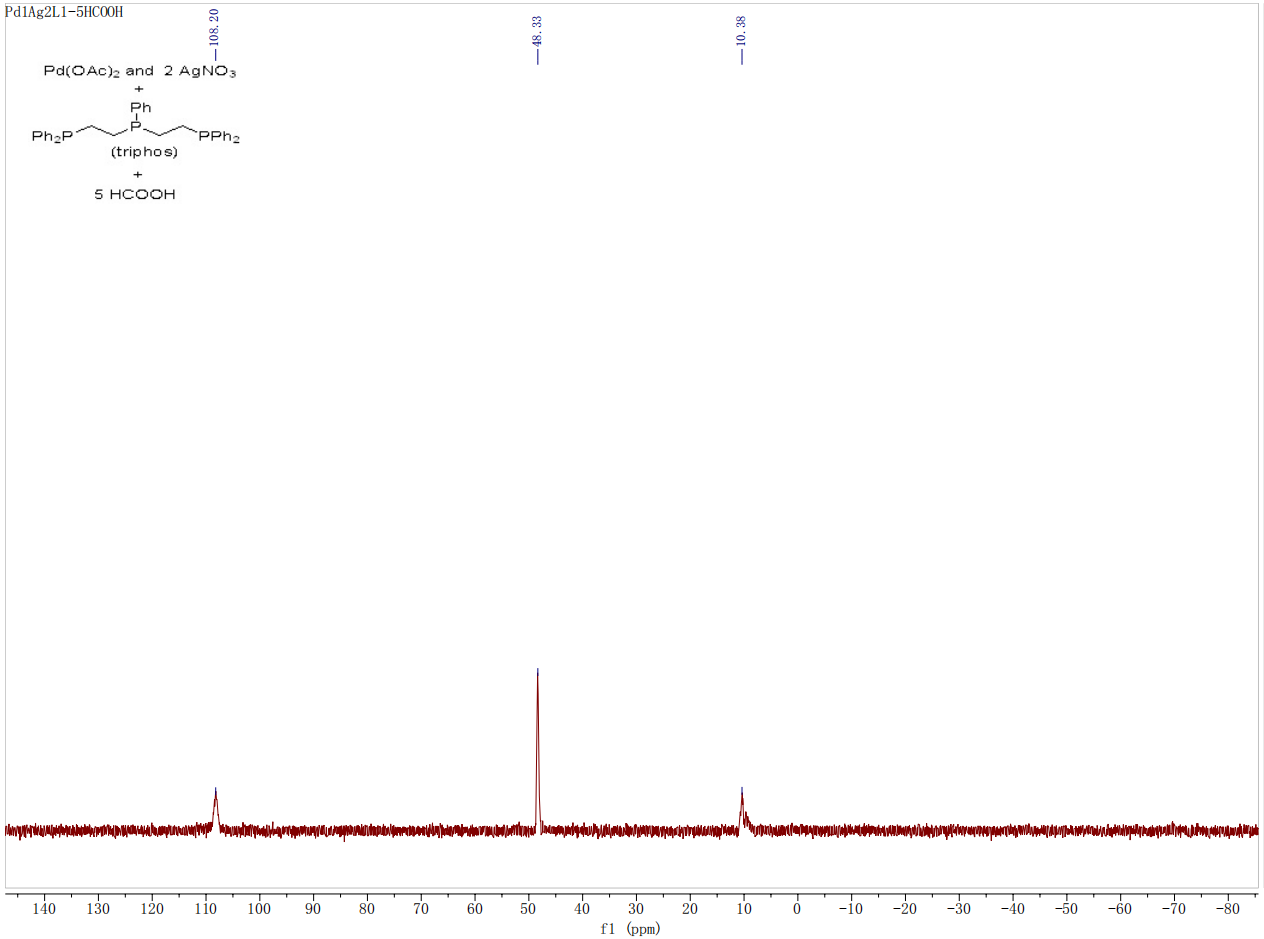


**Figure S13.** ^31^P NMR for Pd(OAc)_2_  + 2 equiv. AgNO_3_ + **L2** + 5 equiv. HCOOH


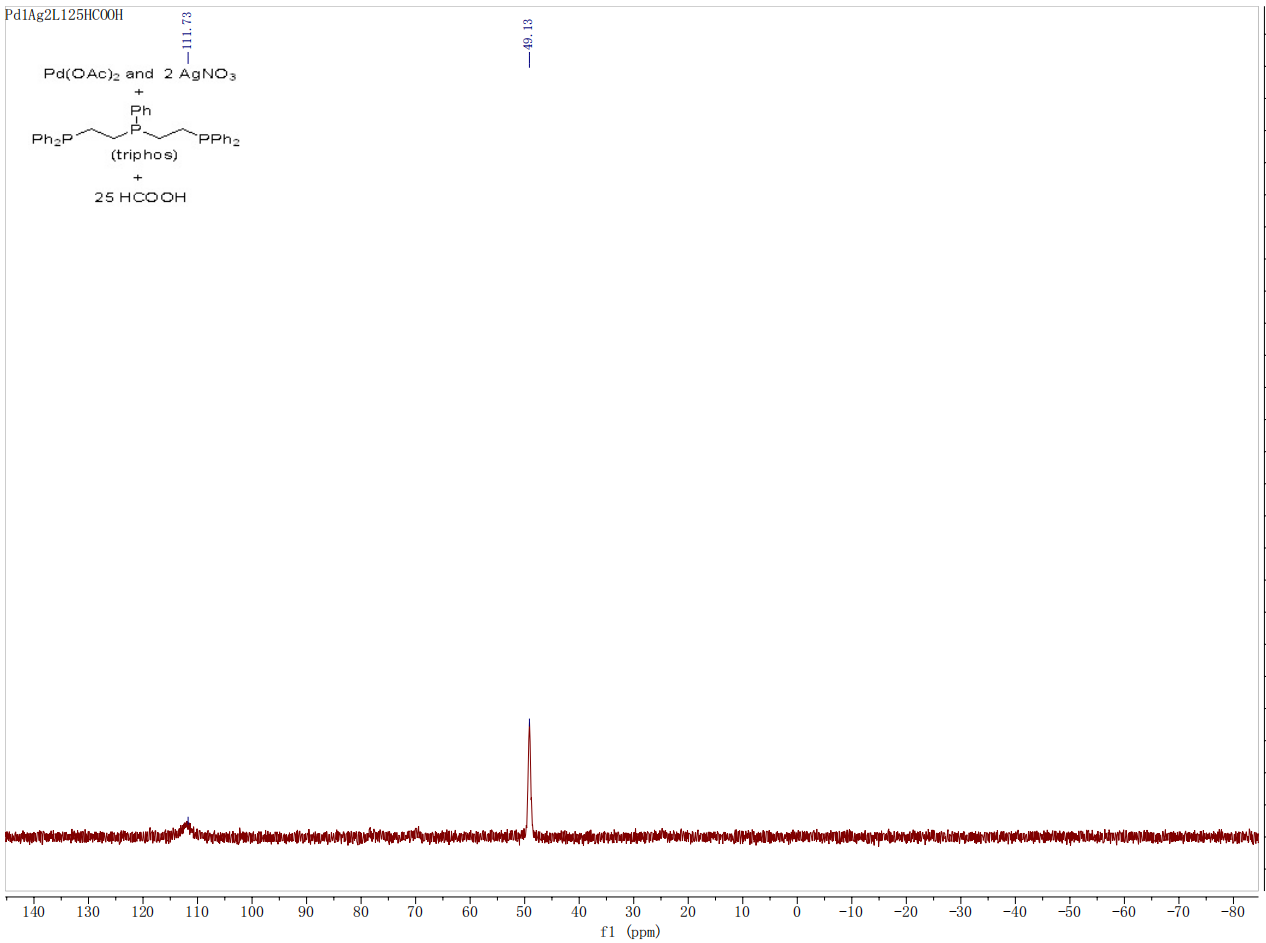


**Figure S14.** ^31^P NMR for Pd(OAc)_2_ + 2 equiv. AgNO_3_ + **L2** + 25 equiv. HCOOH

**7.2. UV-Vis spectrum analysis**


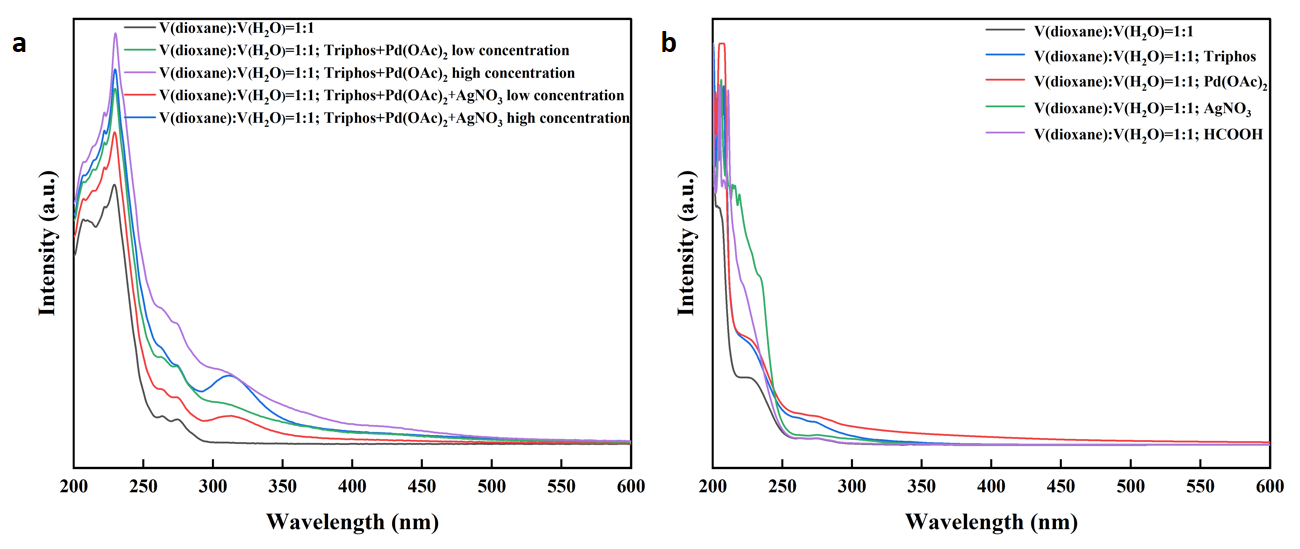


**Figure S15.** (a) UV-Vis spectrum of blank solvent (V1,4-dioxane:VH_2_O = 1:1); different concentration of Pd(OAc)_2_ and linear triphos (**L2)** in solvent (V1,4-dioxane:VH_2_O = 1:1) ; different concentration of AgNO_3_, Pd(OAc)_2_ and linear triphos (**L2)** in solvent (V1,4-dioxane:VH_2_O = 1:1); (b) UV-Vis spectrum of blank solvent; Pd(OAc)_2_; linear triphos (**L2)**; AgNO_3_; HCOOH in solvent (V1,4-dioxane:VH_2_O = 1:1).

**7.3. FT-IR spectrum analysis**


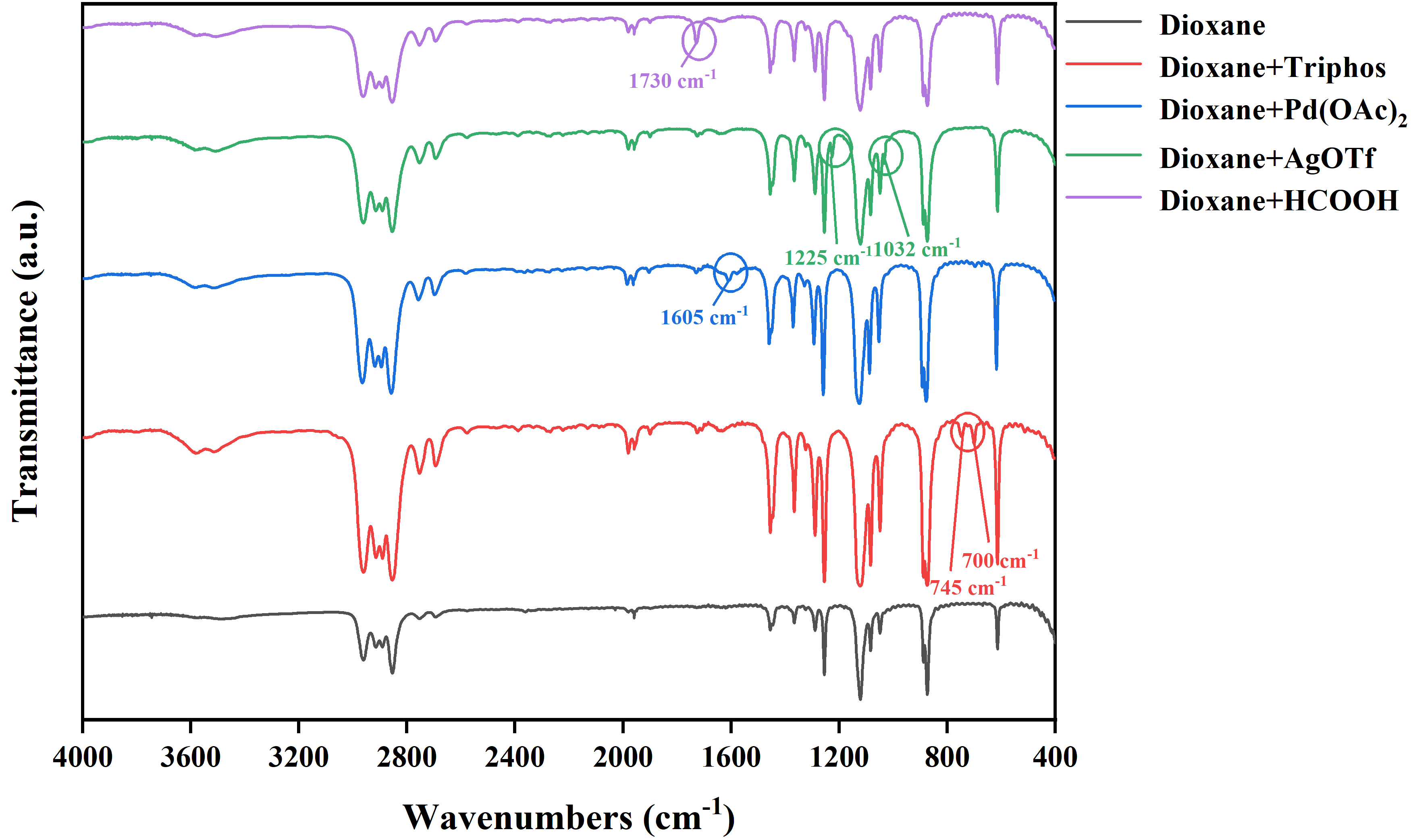


**Figure S16.** FT-IR experiment using blank solvent 1,4-dioxane, linear triphos (**L2)**, Pd(OAc)_2_, AgOTf and HCOOH in 1,4-dioxane.

**7.4. Cyclic voltammograms**


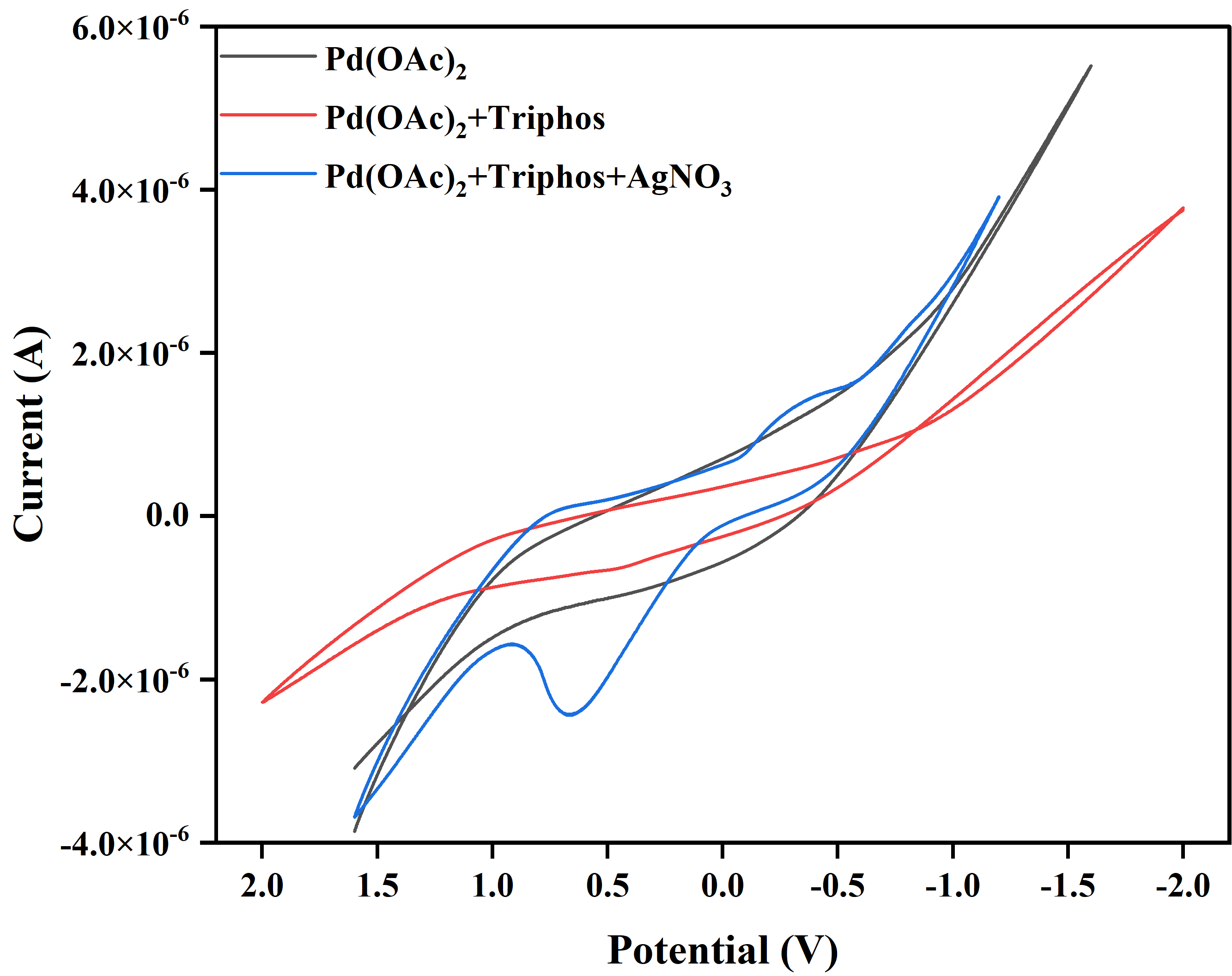


**Figure S17.** Cyclic voltammograms of Pd(OAc)_2_, Pd(OAc)_2_+linear triphos (**L2)**, Pd(OAc)_2_+AgNO_3_+linear triphos (**L2)** in 1,4-dioxane and H_2_O.

**7.5 Intermediates monitoring during catalysis**

The sample was prepared by diluting 10 μL reaction solution (Add 0.12 mol% Pd(OAc)_2_, 0.12 mol% **L2**, 0.24 mol% AgNO_3_, 1 mL H_2_O, and 1 mL 1,4-dioxane to the reaction flask, stirred at 60 °C for 1 hour. Add 440 μL formic acid, continue stirring for 0.5 hours, then stop the reaction.) in 20 mL of distilled water. The ESI-MS experiments were performed under the following conditions: ESI capillary voltage, 2 kV; sample cone voltage, 50 V; source temperature, 120 ^o^C; desolvation temperature, 120 ^o^C; cone gas flow, 100 L/h; desolvation gas flow, 800 L/h (N_2_).


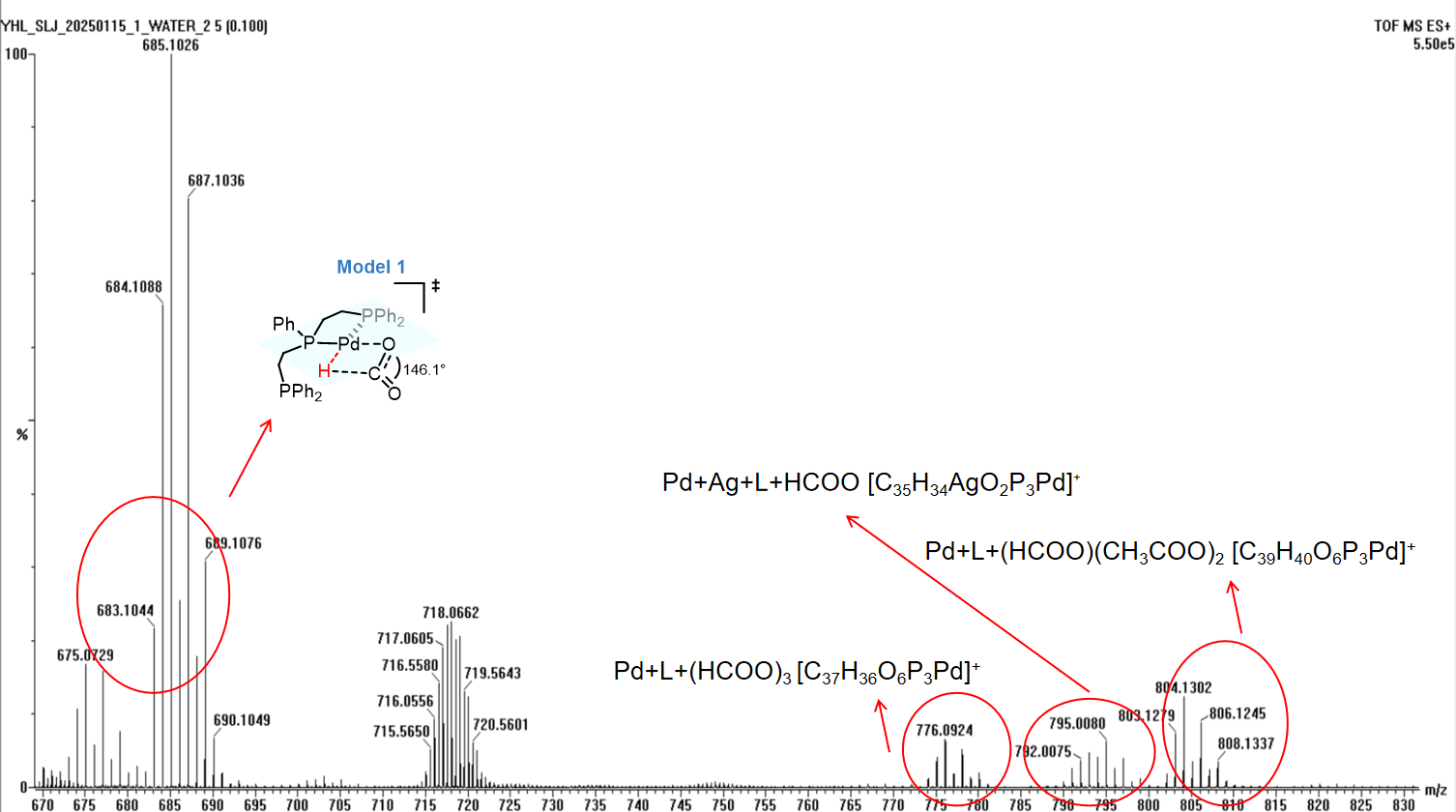


**Figure S18**. ESI-MS spectra of experimental isotopic distributions observed during FA dehydrogenation.

**
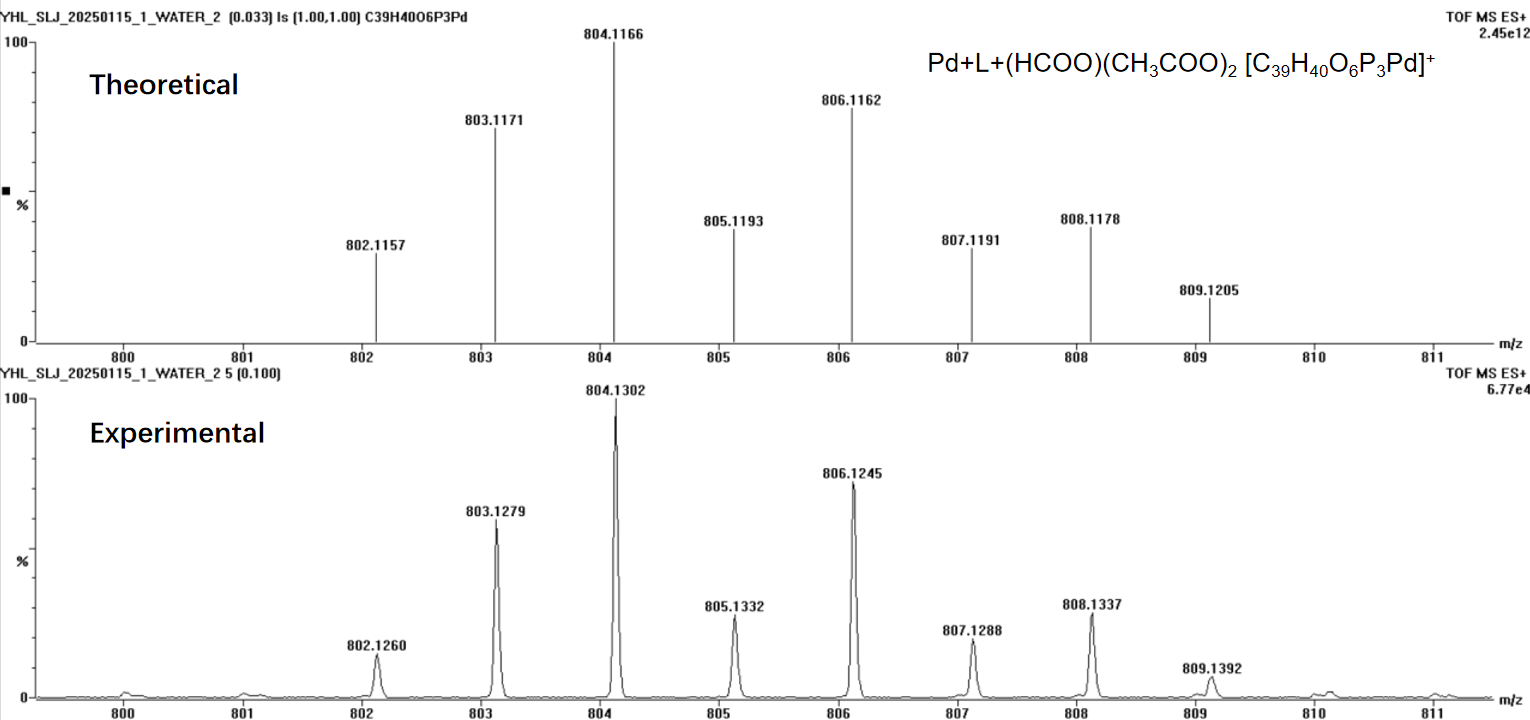
**

**Figure S19**. ESI-MS spectra of experimental and calculated isotopic distributions of Pd+**L2**+(HCOO)(CH_3_COO)_2_ [C_39_H_40_O_6_P_3_Pd]^+^ obtained from the reaction after mixing a solution of HCOOH (440 μL), 1 mL 1,4-dioxane/ 1 mL H_2_O and 0.12 mol% Pd(OAc)_2_, 0.12 mol% linear triphos (**L2**), 0.24 mol% AgNO_3_, 60 ^o^C, in air.

**
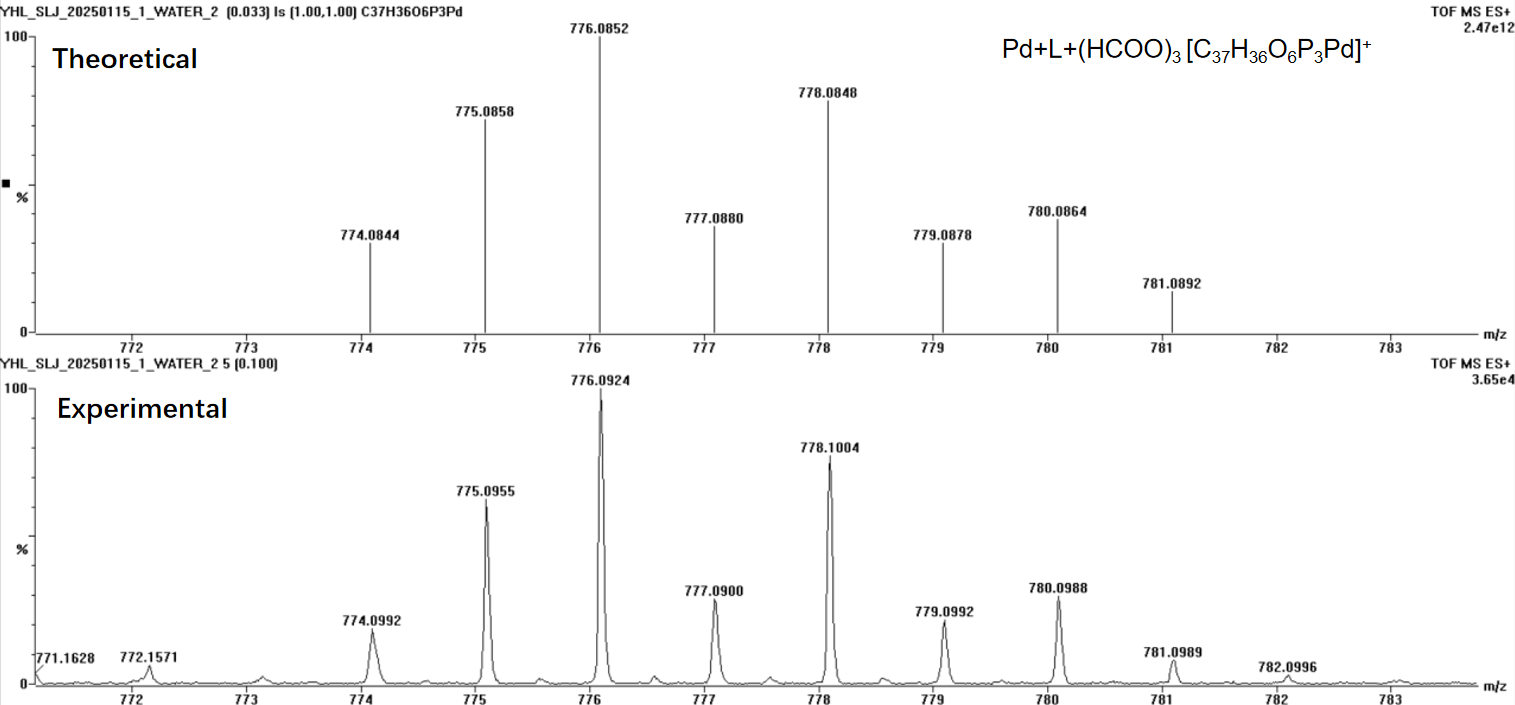
Figure S20**. ESI-MS spectra of experimental and calculated isotopic distributions of Pd+**L2**+(HCOO)_3_ [C_37_H_36_O_6_P_3_Pd]^+^ obtained from the reaction after mixing a solution of HCOOH (440 μL), 1 mL 1,4-dioxane/ 1 mL H_2_O and 0.12 mol% Pd(OAc)_2_, 0.12 mol% linear triphos (**L2**), 0.24 mol% AgNO_3_, 60 ^o^C, in air.


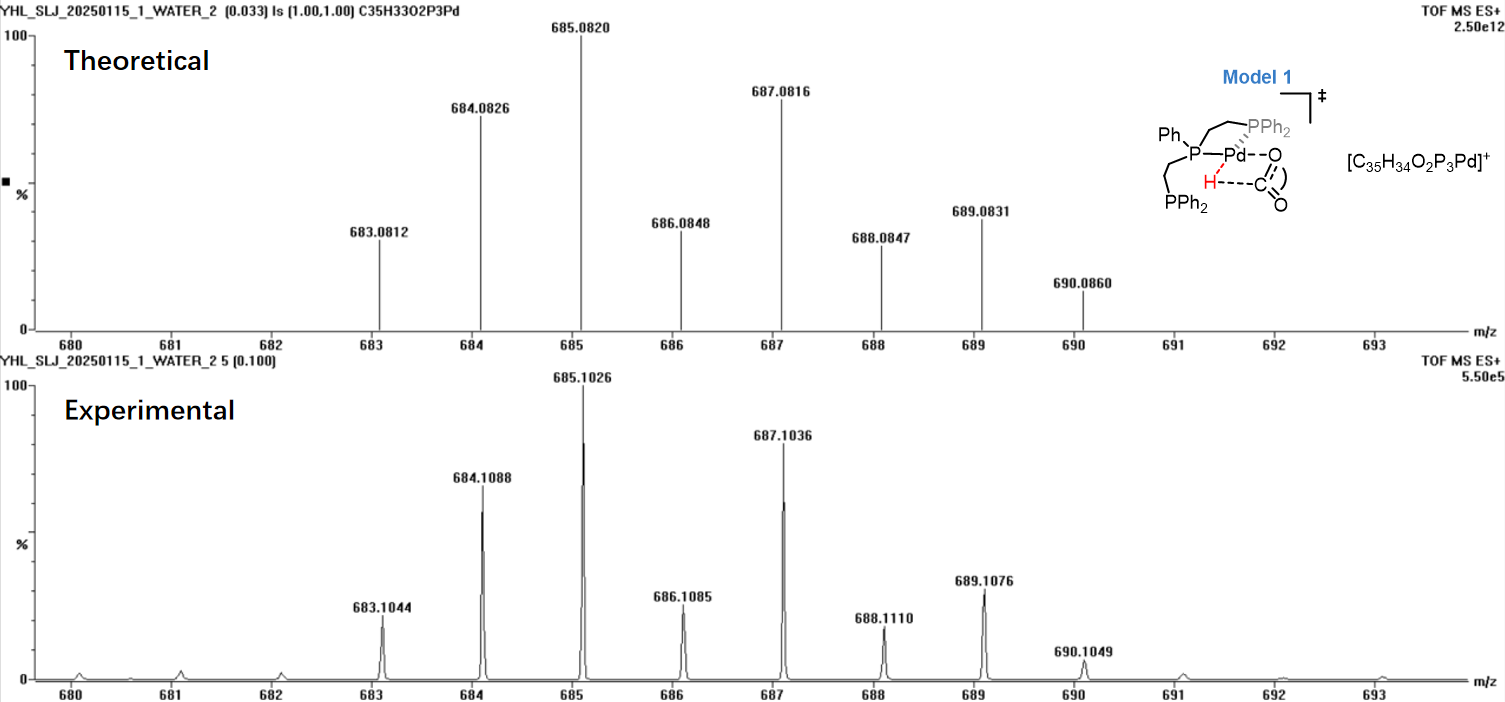


**Figure S21**. ESI-MS spectra of experimental and calculated isotopic distributions of **Model 1** obtained from the reaction after mixing a solution of HCOOH (440 μL), 1 mL 1,4-dioxane/ 1 mL H_2_O and 0.12 mol% Pd(OAc)_2_, 0.12 mol% linear triphos (**L2**), 0.24 mol% AgNO_3_, 60 ^o^C, in air.


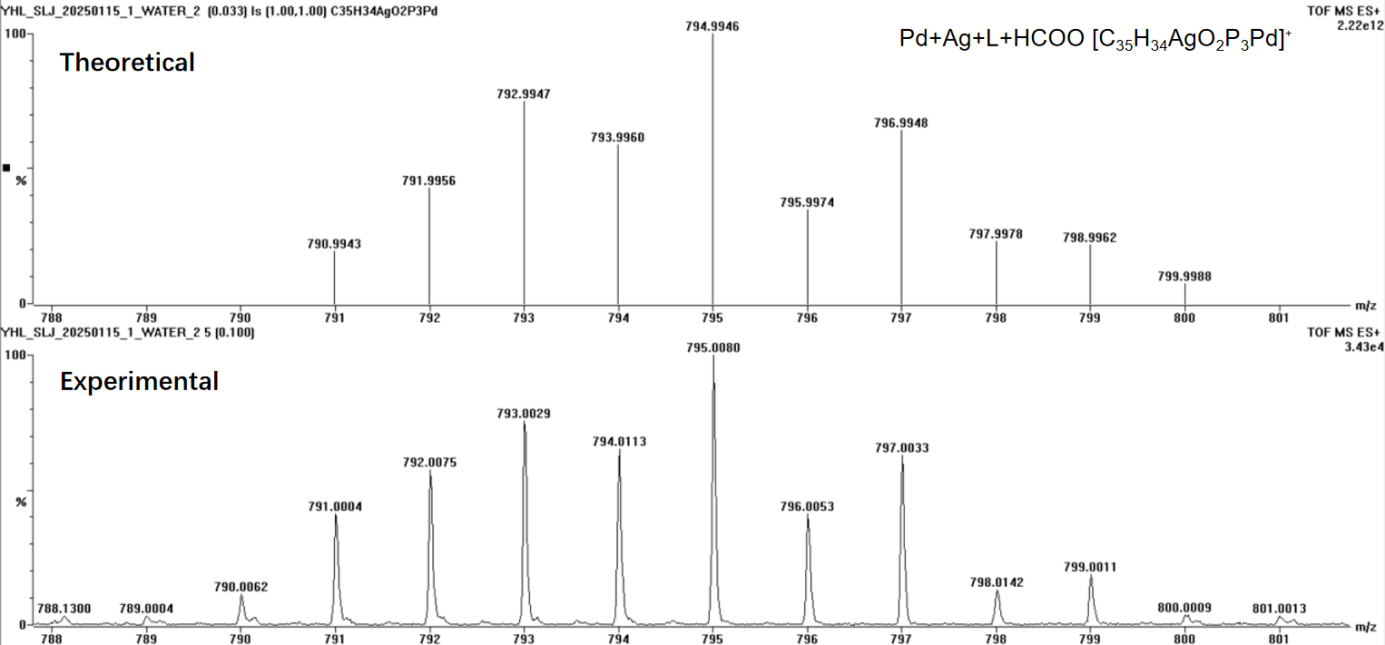


**Figure S22**. ESI-MS spectra of experimental and calculated isotopic distributions of Pd_1_-Ag_1_-linear triphos (Pd+Ag+L+HCOO, [**Model 2**-HCOO]^+^) obtained from the reaction after mixing a solution of HCOOH (440 μL), 1 mL 1,4-dioxane/1 mL H_2_O and 0.12 mol% Pd(OAc)_2_, 0.12 mol% **L2**, 0.24 mol% AgNO_3_, 60 ^o^C, in air.

**8. Computational details**

All quantum chemical calculations were performed using Gaussian 16 (Revision A.03)^[7]^. Geometries of all stationary points, including transition states and intermediates, were optimized at the B3LYP/6-31G(d) level of theory^[8-10]^. The 6-31G(d) basis set was used for light atoms (C, H, O, N, P), while the Stuttgart/Dresden effective core potential (SDD) was employed for heavy atoms (Pd, Ag)^[11]^. Solvent effects were incorporated using the SMD continuum solvation model^[12]^, with a mixed solvent system of 1,4-dioxane and water. For solvated systems, the 6-311++G(d,p) basis set was used for light atoms, while retaining the SDD pseudopotential for Pd and Ag. Thermal corrections at 333.15 K were applied to obtain Gibbs free energies. Molecular structures were visualized using CYLview^[13]^.

**9. Screening of initial catalyst structures**

Based on the proposed dehydrogenation mechanism, we performed first-principles calculations on a palladium-only catalytic system. Due to lack of experimental data regarding the precise spatial configuration of the ligand, palladium center, and formate anion in the catalytic complex, we initiated our investigation with a comprehensive conformational analysis. Two distinct structural conformations were systematically evaluated through geometry optimization at the DFT level to determine the thermodynamically most stable configuration. The optimized structure, **INT1**, which exhibited the lowest free energy, was selected for further analysis and is schematically represented in **Figure S23**. This structure features a square-planar coordination geometry around the Pd center, formed by the phosphorous atom of the linear triphos ligand (**L2**) and the formate anion, with key geometric parameters including a C-H bond length of 1.10 Å and an O-C-O bond angle of 125.9^o^. The Gibbs free energy change (ΔG) for the reaction was calculated as [G1(**INT1** + HOAc + OAc^-^)-G2(Pd(OAc)_2_ + **L2** + HCOOH)] × 627.51 = -80.9 kcal/mol, indicating a strongly exergonic process. This substantial negative ΔG value confirms the thermodynamic feasibility of the proposed reaction pathway.

**Figure S23**. Screening of **INT1**.

#

# **10. Classical four-membered toroidal transition state model without Ag^+^**

**INT1** served as the zero-reference point for the relative free energy profile. The catalytic cycle proceeds through a β-H elimination process, initiated by the dissociation of the P3 coordinating atom from the Pd center. Concurrently, the O and H atoms of the formate ligand form strong interactions with the Pd^2+^, stabilizing the square-planar transition state **TS1** in conjunction with the P_1_ and P_2_ coordinating atoms. Structural analysis of **TS1** reveals a Pd-H bond length of 1.71 Å, accompanied by significant elongation of the C-H bond to 1.47 Å and a substantial increase in the O-C-O bond angle to 146.1°. These structural perturbations indicate a pronounced transformation from the initial sp^2^ hybridization state of the formate C atom in **INT1**, characterized by substantial C-H bond cleavage and the concomitant formation of a Pd-H bond. Following the β-H elimination step, the reaction yields a Pd-H intermediate with simultaneous liberation of CO_2_. The catalytic cycle is completed through the interaction of the resulting intermediate **INT2-1** with another formic acid molecule, which facilitates H_2_ evolution and regenerates the catalytically active **INT1** species. This rate-determining step exhibits an activation energy barrier of 29.8 kcal/mol, as derived from the free energy profile.

**Figure S24.** Formic acid dehydrogenation **Model 1**.

# **Formic acid dehydrogenation Model 2 and more unfavorable species**

In the mono-silver catalytic model, the dissociation of the P_3_ ligand from the Pd center triggers a reorganization of the coordination sphere, resulting in a square-planar geometry around the Pd center formed by the oxygen atoms of two formate ligands and the P_1_ and P_2_ ligands. The Ag^+^ is positioned below the Pd coordination plane, with a Pd-Ag bond distance of 2.93 Å and an O_1_-C-O_2_ bond angle of 128.2°. The C-H bond length of the formate ligand remains at 1.10 Å. To elucidate the preferred metal site (Pd *vs*. Ag) for β-H elimination and identify the optimal formate ligand configuration, we systematically investigated three distinct transition state models. In **TS2**, the Pd-Ag bond distance decreases from 2.93 Å in **INT3** to 2.88 Å, while the O_3_-Ag_1_ distance shortens to 2.48 Å, highlighting the bridging role of the formate ligand. The Pd-H bond length is 1.71 Å, and the C-H bond elongates to 1.37 Å, accompanied by an increase in the O_1_-C-O_2_ bond angle to 143.3° (compared to 128.2° in **INT3**). These structural alterations strongly indicate that β-H elimination occurs preferentially at the Pd site. Additionally, the Ag_1_-O_2_ bond length remains at 2.24 Å, within a strong interaction range, underscoring Ag’s role in stabilizing the emerging carbonyl moiety.

In contrast, **TS2-1** represents β-H elimination at the Ag catalytic site. After solvation corrections, this pathway exhibits a free energy of 10.7 kcal/mol, significantly higher than that of the Pd-catalyzed process, suggesting that Ag alone is energetically less favorable for hydrogen extraction. Finally, **TS2-2** explores β-H elimination at the P_1_ position under Pd catalysis. Although the energy difference between **TS2** and **TS2-2** is minimal (2 kcal/mol), it is not statistically significant.

Based on these findings, we propose a formate-bridged Pd-Ag cooperative catalysis mechanism for β-H elimination, as illustrated in **TS2**, where Pd and Ag act synergistically to facilitate the reaction. The β-H elimination generates a Pd-H species and releases CO_2_. The resulting intermediate **Int3-1** then interacts with formic acid to release H_2_ and regenerate **INT3**, completing the catalytic cycle. The calculated energy barrier for this process is 17.3 kcal/mol, consistent with the proposed cooperative mechanism.

**Figure S25.** Computational insights into β-H elimination reactions in **Model 2**.

#

# **12. Ag-H agnostic interaction and other computational insights in formic acid dehydrogenation Model 3**

In **Model 3**, consistent with the previous observations, the formation of new intermediates (**INT3** and **INT4**) is accompanied by the dissociation of the P_3_ ligand. The oxygen atoms of the two formate ligands, together with the P_1_ and P_2_ ligands, form a square-planar coordination geometry around the Pd center. The O_2_-C-O_1_ bond angle is measured at 126.0°, and the C-H bond length of the formate ligand remains at 1.10 Å.

For β-H elimination at the Ag site, the transition state **TS3-1** exhibits a distinct structural configuration. Notably, the bond length between Ag_2_ and the central Pd metal elongates significantly to 3.22 Å, strongly indicating the non-participation of Ag_2_ in this β-H elimination step. Further analysis of the solvation free energy difference (8.8 kcal/mol) underscores the energetic preference for **TS3**, confirming that the Pd site serves as the primary active center for this reaction.

Extending the investigation of β-H elimination at the P_1_ and P_2_ positions from the single-Ag system to the dual-Ag system, we find that β-H elimination at the P1 position (**TS3**) is more favorable than at the P_2_ position. This preference arises because the latter relies solely on a single Ag atom to stabilize the carbonyl oxygen. By comparing the energy difference between β-H elimination at the P1 position with dual Ag atoms (**TS3**) and β-H elimination at the P_1_ position with a single Ag atom (**TS2-2**), we observe that **TS3** not only reverses the energetic disadvantage of Ag_1_-mediated β-H elimination in the single-Ag system (where **TS2-2** is 2 kcal/mol higher than **TS2**) but also achieves energy compensation through the synergistic interaction of the two Ag atoms with the carbonyl oxygen.

Further comparison reveals that in the single-Ag system, the energy difference between Ag-mediated β-H elimination (**TS2-1**) and Pd-mediated β-H elimination (**TS2**) is as high as 12.9 kcal/mol. In contrast, in the dual-Ag system, this energy difference is reduced to 8.8 kcal/mol, highlighting the aggregation tendency of Ag atoms in multimetallic systems and their role in enhancing catalytic efficiency. These findings underscore the importance of multimetallic cooperation in optimizing catalytic performance.

**Figure S26.** Computational insights into β-H elimination reactions in **Model 3**.

#

# **13. Energies of all optimized compounds and transition states were determined using the B3LYP method**

| **Complex** | **E(opt) (a.u.)** | **Thermal Correction to Enthalpy (a.u.)** | **Thermal Correction to Free Energy (a.u.)** | **G(opt) (a.u.)** |
| --- | --- | --- | --- | --- |
| **Int1** | -2656.643844 | 0.642505 | 0.527953 | -2656.115891 |
| **Int1’** | -2656.607832 | 0.642451 | 0.528346 | -2656.079486 |
| **Int2-1** | -2468.010339 | 0.621577 | 0.512643 | -2467.497696 |
| **Int3** | -2992.903361 | 0.673360 | 0.546199 | -2992.357161 |
| **Int3-1** | -2804.289576 | 0.653019 | 0.530895 | -2803.758681 |
| **Int4** | -3420.220781 | 0.694189 | 0.545743 | -3419.675038 |
| **Int4-1** | -3231.656490 | 0.675571 | 0.538615 | -3231.117876 |
| **TS1** | -2656.574498 | 0.636018 | 0.517165 | -2656.057332 |
| **TS2** | -2992.991114 | 0.668475 | 0.539908 | -2992.451206 |
| **TS2-1** | -2992.845560 | 0.669534 | 0.542047 | -2992.303514 |
| **TS2-2** | -2992.836895 | 0.667196 | 0.538968 | -2992.297927 |
| **TS3** | -3420.220323 | 0.692527 | 0.546313 | -3419.674010 |
| **TS3’** | -3496.668132 | 0.719516 | 0.567436 | -3496.100696 |
| **TS3-1** | -3420.214196 | 0.691238 | 0.545851 | -3419.668345 |
| **AgOOCH** | -336.172885 | 0.027997 | -0.006348 | -336.179234 |
| **AgNO_3_** | -427.299392 | 0.021111 | -0.015052 | -427.314444 |
| **H_2_O** | -76.408962 | 0.024944 | 0.003498 | -76.405464 |

| **Complex** | **E(sol) Hartree** | **Temperature correction (333.15K)** | **∆G(sol) (kcal/mol)** |
| --- | --- | --- | --- |
| **Int1** | -2657.227653 | 0.514009 |  |
| **Int2-1** | -2468.531884 | 0.499389 | 12.7 |
| **Int3** | -2993.54736 | 0.530719 | -19.5 |
| **Int3-1** | -2804.875554 | 0.516038 | -21.8 |
| **Int4** | -3421.001158 | 0.530343 | -25.4 |
| **Int4-1** | -3232.339206 | 0.52196 | -30.0 |
| **TS1** | -2657.167639 | 0.501439 | 29.8 |
| **TS2** | -2993.511971 | 0.522775 | -2.2 |
| **TS2-1** | -2993.512296 | 0.526411 | 10.7 |
| **TS2-2** | -2993.490534 | 0.522029 | -0.2 |
| **TS3** | -3420.974908 | 0.524614 | -12.5 |
| **TS3’** | -3497.458772 | 0.548941 | -8.6 |
| **TS3-1** | -3420.965072 | 0.528941 | -3.7 |
| **AgOOCH** | -336.262284 | -0.009695 |  |
| **AgNO_3_** | -427.426058 | -0.018619 |  |
| **H_2_O** | -76.466836 | 0.000955 |  |
| **CO_2_** | -188.648849 | -0.012013 |  |
| **H_2_** | -1.179002 | -0.003101 |  |

| **INT1, Cartesian coordinates:**  Pd -0.000218 0.562919 0.086358  P 2.312808 0.583543 0.430087  P 0.000219 -1.069891 1.673646  P -2.313355 0.582218 0.429962  C 3.199270 -0.799975 -0.351680  C 4.305376 -1.420960 0.253247  C 2.708670 -1.282344 -1.577384  C 4.907176 -2.522106 -0.357449  H 4.700077 -1.056271 1.197613  C 3.323028 -2.378460 -2.184523  H 1.845996 -0.812492 -2.044841  C 4.414412 -3.001867 -1.575121  H 5.760053 -3.001727 0.113985  H 2.939771 -2.749747 -3.130207  H 4.884594 -3.859427 -2.047882  C 3.217958 2.124247 0.107503  C 4.577836 2.120867 -0.240534  C 2.528351 3.341882 0.233087  C 5.244501 3.329511 -0.440505  H 5.111509 1.183392 -0.364050  C 3.204633 4.545192 0.037119  H 1.464809 3.344373 0.449731  C 4.561023 4.539848 -0.298197  H 6.296120 3.325335 -0.711536  H 2.669111 5.485424 0.129464  H 5.083214 5.478488 -0.459116  C -3.219942 2.122057 0.107130  C -4.580550 2.117367 -0.238063  C -2.530885 3.340309 0.229857  C -5.248447 3.325313 -0.438112  H -5.113863 1.179400 -0.359402  C -3.208398 4.542921 0.033796  H -1.466903 3.343749 0.444234  C -4.565473 4.536273 -0.298698  H -6.300620 3.320100 -0.706962  H -2.673334 5.483633 0.123885  H -5.088607 5.474377 -0.459688  C -3.198649 -0.802134 -0.351711  C -4.303841 -1.424469 0.253515  C -2.708039 -1.283696 -1.577723  C -4.904723 -2.526094 -0.357208  H -4.698491 -1.060485 1.198174  C -3.321478 -2.380323 -2.184890  H -1.846031 -0.812848 -2.045411  C -4.411959 -3.005029 -1.575210  H -5.756884 -3.006758 0.114459  H -2.938199 -2.750956 -3.130821  H -4.881441 -3.862965 -2.047983  C 0.001400 -2.777120 1.018142  C 1.213006 -3.418130 0.704823  C -1.209187 -3.419329 0.703407  C 1.209956 -4.678136 0.109004  H 2.167000 -2.943159 0.898203  C -1.204178 -4.679341 0.107601  H -2.163886 -2.945316 0.895665  C 0.003378 -5.313216 -0.190165  H 2.156299 -5.155099 -0.126514  H -2.149771 -5.157264 -0.128986  H 0.004139 -6.294151 -0.656095  C 2.409445 0.309768 2.277722  C 1.537370 -0.879191 2.734605  H 2.037674 1.245363 2.709448  H 3.444364 0.190963 2.614887  H 1.225743 -0.753084 3.776687  H 2.096117 -1.816754 2.683012  C -1.537733 -0.881003 2.733744  C -2.409855 0.308155 2.277544  H -1.227083 -0.756184 3.776274  H -2.096159 -1.818661 2.680382  H -3.444725 0.189206 2.614820  H -2.038009 1.243608 2.709522  C -0.000471 1.617394 -2.493563  O -0.000232 0.409974 -2.761454  O -0.000354 2.131094 -1.305855  H -0.000876 2.375992 -3.297004 | **INT1’, Cartesian coordinates:**  Pd -0.128761 -0.142772 -1.166348  P -0.054287 1.680416 0.200075  P 2.231590 -0.770477 0.724162  P -1.155606 -1.512854 0.356466  C 1.119780 2.911008 -0.465180  C 0.912576 4.276304 -0.205303  C 2.274348 2.499396 -1.151913  C 1.858248 5.214772 -0.617920  H 0.017258 4.607716 0.311978  C 3.214269 3.445709 -1.559817  H 2.428724 1.452896 -1.387582  C 3.009404 4.801368 -1.292711  H 1.692245 6.268734 -0.415578  H 4.100034 3.122663 -2.098882  H 3.741202 5.535601 -1.616582  C -1.655669 2.521518 0.369592  C -2.287587 2.727269 1.603550  C -2.284800 2.943374 -0.815993  C -3.534582 3.353213 1.650297  H -1.823931 2.405528 2.529877  C -3.525782 3.572799 -0.760750  H -1.799168 2.781536 -1.775355  C -4.152427 3.777384 0.472981  H -4.020600 3.508651 2.608723  H -4.003767 3.903624 -1.678049  H -5.120879 4.267247 0.514764  C -2.732945 -0.873277 0.999046  C -3.126705 -1.016199 2.338734  C -3.588039 -0.224178 0.090871  C -4.357121 -0.509416 2.762192  H -2.495096 -1.531882 3.054888  C -4.817960 0.269445 0.517457  H -3.283513 -0.098826 -0.944501  C -5.201636 0.131803 1.854395  H -4.656710 -0.624273 3.799730  H -5.467716 0.776150 -0.188767  H -6.158033 0.523943 2.187579  C -1.495780 -3.112550 -0.446167  C -0.451777 -3.769697 -1.120839  C -2.760690 -3.714867 -0.357871  C -0.670825 -5.026313 -1.681403  H 0.515241 -3.291463 -1.237671  C -2.970935 -4.970680 -0.928148  H -3.574939 -3.211972 0.153762  C -1.928664 -5.627469 -1.585970  H 0.136437 -5.528289 -2.206164  H -3.950408 -5.434361 -0.857752  H -2.098001 -6.603765 -2.030510  C 4.012130 -1.092468 1.050437  C 4.921619 -0.093132 0.655392  C 4.515275 -2.285838 1.590682  C 6.292652 -0.270102 0.828307  H 4.557995 0.830061 0.207689  C 5.891745 -2.465512 1.750446  H 3.848039 -3.088155 1.889410  C 6.782150 -1.458788 1.377083  H 6.980078 0.515467 0.527546  H 6.265371 -3.394914 2.170929  H 7.851145 -1.600533 1.505670  C 0.575158 1.346482 1.907731  C 1.952635 0.651303 1.938367  H -0.188839 0.762554 2.426824  H 0.639207 2.313647 2.420643  H 2.163724 0.312695 2.959895  H 2.722184 1.388411 1.687754  C 1.370728 -2.185403 1.601945  C -0.133146 -1.967889 1.848465  H 1.843380 -2.366762 2.574625  H 1.522941 -3.086665 0.997390  H -0.576410 -2.885561 2.252046  H -0.278611 -1.189937 2.601034  C 0.381441 -0.407390 -3.591707  O -0.072619 -1.392796 -2.930294  O 0.574663 0.713002 -3.019936  H 0.610073 -0.522038 -4.659748 |
| --- | --- |
|  |  |

| **INT2-1, Cartesian coordinates:**  Pd 0.527605 -0.275402 -0.910220  P 0.173237 1.857763 0.107854  P 2.052050 -0.659624 0.764331  P -1.291655 -1.537894 -0.079953  C 1.290280 3.150055 -0.551729  C 1.165774 4.487445 -0.138014  C 2.317161 2.795524 -1.440737  C 2.059149 5.449881 -0.605082  H 0.364394 4.777628 0.535820  C 3.208861 3.763796 -1.907067  H 2.421636 1.763461 -1.767282  C 3.081020 5.089359 -1.489244  H 1.956668 6.481889 -0.282517  H 3.998106 3.482126 -2.597946  H 3.773281 5.842789 -1.853508  C -1.460840 2.661272 0.242080  C -2.270322 2.544743 1.381485  C -1.947267 3.359786 -0.876817  C -3.535809 3.131738 1.406677  H -1.930884 1.999043 2.255205  C -3.212596 3.943377 -0.846705  H -1.328851 3.464857 -1.764400  C -4.008024 3.833068 0.296676  H -4.153089 3.034413 2.294361  H -3.573938 4.489104 -1.713462  H -4.992804 4.290386 0.320783  C -2.919421 -0.803783 0.316991  C -3.569953 -0.972388 1.550515  C -3.547315 -0.056109 -0.692074  C -4.832049 -0.414076 1.760990  H -3.107173 -1.544504 2.349240  C -4.811051 0.491815 -0.481559  H -3.038236 0.103687 -1.638969  C -5.455462 0.312723 0.744780  H -5.329832 -0.553648 2.716465  H -5.284452 1.072006 -1.267495  H -6.438325 0.744259 0.910587  C -1.643121 -3.240423 -0.657820  C -2.549894 -4.058054 0.038912  C -1.005065 -3.740525 -1.800901  C -2.795836 -5.359443 -0.393461  H -3.077309 -3.675136 0.908376  C -1.254184 -5.045803 -2.231665  H -0.317385 -3.109117 -2.355775  C -2.145780 -5.855993 -1.527912  H -3.497919 -5.984897 0.150303  H -0.755769 -5.424396 -3.119293  H -2.341458 -6.869902 -1.864638  C 3.740678 -1.325723 0.726797  C 4.304027 -1.665705 -0.512128  C 4.481935 -1.515974 1.905778  C 5.594730 -2.193975 -0.571530  H 3.728512 -1.516271 -1.421944  C 5.772090 -2.039733 1.839650  H 4.058619 -1.260043 2.873713  C 6.327985 -2.378968 0.602160  H 6.026906 -2.457052 -1.532419  H 6.343227 -2.184944 2.751912  H 7.333404 -2.786923 0.555021  C 0.777903 1.620504 1.864049  C 2.095830 0.825918 1.896055  H 0.001085 1.086135 2.418390  H 0.904936 2.600159 2.336800  H 2.338109 0.524934 2.921915  H 2.921408 1.447554 1.531431  C 1.038912 -1.978020 1.638229  C -0.499912 -1.788259 1.619594  H 1.381494 -2.068696 2.676728  H 1.305633 -2.910625 1.128128  H -0.960240 -2.661780 2.095051  H -0.780192 -0.914012 2.215423  H 1.021565 -1.685948 -1.421183 | **INT3, Cartesian coordinates:**  Pd 1.600288 0.473834 0.658948  P 1.907394 -1.305152 -0.755387  P 1.037522 1.665328 -1.189949  P -2.472938 0.007591 -0.160381  C 3.557355 -2.058692 -0.585000  C 4.545461 -1.435029 0.191275  C 3.845638 -3.256771 -1.260236  C 5.819182 -2.001185 0.275713  H 4.317347 -0.530430 0.746046  C 5.119414 -3.813852 -1.171282  H 3.075474 -3.760254 -1.839310  C 6.106885 -3.184805 -0.405697  H 6.581044 -1.520347 0.881904  H 5.340377 -4.741004 -1.691784  H 7.097491 -3.624318 -0.334808  C 0.689331 -2.653585 -0.579048  C 0.378644 -3.072346 0.727564  C 0.071995 -3.284416 -1.669429  C -0.548328 -4.091343 0.934437  H 0.865138 -2.602402 1.574817  C -0.858093 -4.303984 -1.453793  H 0.309954 -3.000677 -2.689911  C -1.173068 -4.703904 -0.155452  H -0.786396 -4.401509 1.947570  H -1.339177 -4.779972 -2.302317  H -1.907385 -5.485952 0.007047  C -3.298922 -1.587128 -0.517430  C -3.739435 -2.346827 0.578414  C -3.550889 -2.056781 -1.816189  C -4.432596 -3.540524 0.380720  H -3.540310 -2.002746 1.590503  C -4.235372 -3.257786 -2.011561  H -3.224179 -1.494350 -2.684923  C -4.681184 -3.998635 -0.915247  H -4.773865 -4.113413 1.238037  H -4.426487 -3.609259 -3.021536  H -5.220438 -4.928665 -1.070274  C -3.794682 1.274238 -0.157618  C -4.982558 1.087623 -0.884292  C -3.592849 2.467333 0.556903  C -5.954349 2.087263 -0.899578  H -5.158208 0.161492 -1.423667  C -4.570102 3.464959 0.528467  H -2.676848 2.626654 1.117137  C -5.749082 3.276907 -0.194845  H -6.874640 1.934395 -1.455995  H -4.409333 4.385823 1.081696  H -6.510582 4.051443 -0.205463  C 1.622327 3.383798 -1.195712  C 2.885501 3.641821 -0.635402  C 0.875807 4.440263 -1.739848  C 3.399204 4.937081 -0.635204  H 3.452752 2.838367 -0.173740  C 1.394772 5.735308 -1.731302  H -0.108462 4.272295 -2.163227  C 2.654204 5.984914 -1.182369  H 4.373298 5.129467 -0.195772  H 0.811314 6.549193 -2.151217  H 3.051826 6.995453 -1.174831  C 1.799287 -0.694870 -2.527915  C 1.954756 0.833280 -2.585423  H 0.833804 -0.990376 -2.944172  H 2.575679 -1.193152 -3.116316  H 1.628885 1.235747 -3.551637  H 3.004060 1.118794 -2.450367  C -0.740453 1.715633 -1.706970  C -1.530375 0.387729 -1.725194  H -0.733944 2.134356 -2.720672  H -1.220012 2.449171 -1.052991  H -2.269536 0.448348 -2.530789  H -0.905007 -0.483941 -1.930116  C 0.549556 2.886429 1.951266  O 1.587606 2.128882 1.915052  O -0.491250 2.743252 1.290725  H 0.650503 3.731102 2.654451  Ag -1.068608 -0.060667 1.743431  C 1.442694 -0.680534 3.381031  O 2.203219 -0.618208 2.363884  O 0.203935 -0.475875 3.439096  H 1.941440 -0.974798 4.316936 |
| --- | --- |
|  |  |

| **INT3-1, Cartesian coordinates:**  Pd 1.433326 0.450427 0.977656  P 2.255276 -1.139274 -0.388254  P 0.819063 1.713772 -0.969868  P -2.579885 -0.159547 -0.280069  C 4.075703 -1.252779 -0.373625  C 4.740710 -2.067079 -1.308283  C 4.823327 -0.509907 0.552356  C 6.132477 -2.132900 -1.313470  H 4.175215 -2.665811 -2.017870  C 6.218284 -0.576247 0.538855  H 4.314855 0.113094 1.282426  C 6.872193 -1.384753 -0.391767  H 6.639889 -2.767923 -2.033679  H 6.790589 0.002312 1.257816  H 7.957011 -1.436393 -0.399402  C 1.587108 -2.827023 -0.184846  C 0.216540 -3.030459 -0.412694  C 2.375376 -3.894986 0.263795  C -0.356770 -4.284647 -0.217500  H -0.415785 -2.209250 -0.732031  C 1.796828 -5.150775 0.464763  H 3.433353 -3.752638 0.458286  C 0.436362 -5.348640 0.222632  H -1.416801 -4.424186 -0.403772  H 2.414089 -5.974253 0.811619  H -0.006337 -6.327957 0.379332  C -3.196532 -1.827153 -0.704028  C -3.209704 -2.337231 -2.011668  C -3.687703 -2.619869 0.347431  C -3.707398 -3.618653 -2.260132  H -2.838004 -1.748067 -2.844044  C -4.197062 -3.892425 0.093308  H -3.670418 -2.241436 1.366911  C -4.205098 -4.394816 -1.211417  H -3.709583 -4.006547 -3.274499  H -4.578255 -4.493810 0.913182  H -4.595298 -5.388805 -1.409048  C -4.041027 0.942975 -0.264885  C -5.102945 0.740554 -1.161634  C -4.075166 2.035988 0.615144  C -6.179833 1.625379 -1.177982  H -5.096630 -0.113093 -1.834171  C -5.153467 2.922931 0.589573  H -3.268267 2.192090 1.327530  C -6.204852 2.717776 -0.305297  H -7.002380 1.460157 -1.867674  H -5.174671 3.764846 1.275173  H -7.047510 3.402719 -0.318713  C 1.322594 3.462080 -1.076613  C 1.678345 4.132721 0.104989  C 1.363069 4.144883 -2.304654  C 2.066943 5.473747 0.052157  H 1.649593 3.611377 1.057322  C 1.756329 5.481141 -2.349365  H 1.095191 3.638037 -3.228749  C 2.107403 6.146264 -1.170146  H 2.341904 5.989367 0.967697  H 1.790131 6.003525 -3.301074  H 2.413606 7.187717 -1.207658  C 1.824604 -0.670331 -2.153579  C 1.763973 0.855016 -2.343219  H 0.864911 -1.141057 -2.384017  H 2.563995 -1.114394 -2.827973  H 1.346203 1.104283 -3.325515  H 2.775232 1.276298 -2.305255  C -0.938143 1.775766 -1.608015  C -1.665374 0.427261 -1.794090  H -0.895251 2.295330 -2.572532  H -1.498298 2.435979 -0.935228  H -2.416568 0.534148 -2.584887  H -0.981445 -0.362792 -2.113226  Ag -1.337065 -0.012859 1.730470  C 0.322969 1.619482 3.520814  O 1.063247 1.873545 2.525764  O -0.593614 0.756363 3.596393  H 0.497659 2.232852 4.417999  H 1.922902 -0.554253 2.095606 | **INT4, Cartesian coordinates:**  Pd -1.826886 0.870594 -0.512666  P -2.344267 -0.109987 1.482441  P -0.723482 2.457050 0.695844  P 3.001954 0.276686 0.675926  C -4.127063 -0.342310 1.691911  C -4.911218 0.647754 2.306500  C -4.742272 -1.473992 1.128628  C -6.295536 0.495262 2.375958  H -4.454435 1.537235 2.732051  C -6.126325 -1.618156 1.204033  H -4.146556 -2.232458 0.629895  C -6.903122 -0.636714 1.826463  H -6.898193 1.258422 2.858984  H -6.597714 -2.496729 0.774058  H -7.981326 -0.752750 1.881638  C -1.518525 -1.713249 1.726048  C -0.311104 -1.979183 1.057386  C -2.024853 -2.647362 2.645654  C 0.385492 -3.162838 1.303542  H 0.087490 -1.269450 0.338840  C -1.320897 -3.827193 2.890508  H -2.963630 -2.462663 3.158775  C -0.119573 -4.084828 2.222826  H 1.304173 -3.358151 0.762682  H -1.715654 -4.547970 3.600370  H 0.420177 -5.007303 2.414493  C 3.432805 -1.159012 1.725674  C 3.945520 -2.300887 1.085706  C 3.228879 -1.188926 3.114972  C 4.249547 -3.446559 1.821177  H 4.092857 -2.300828 0.008486  C 3.524345 -2.340454 3.846242  H 2.838178 -0.321561 3.638492  C 4.033619 -3.469904 3.201252  H 4.646080 -4.321225 1.314057  H 3.359187 -2.353757 4.919754  H 4.263102 -4.364468 3.772888  C 4.351101 1.502636 0.778149  C 5.161901 1.628949 1.916989  C 4.538909 2.369728 -0.310784  C 6.143859 2.617979 1.965782  H 5.040209 0.946957 2.754053  C 5.517123 3.363841 -0.251500  H 3.930106 2.261817 -1.205895  C 6.319191 3.487541 0.885055  H 6.776980 2.706198 2.843934  H 5.661863 4.028513 -1.098174  H 7.088725 4.252946 0.925265  C -1.140253 4.067130 -0.020492  C -2.494714 4.454637 -0.043198  C -0.171909 4.883040 -0.627100  C -2.866671 5.658516 -0.634600  H -3.261543 3.810155 0.380284  C -0.554118 6.088712 -1.214179  H 0.866851 4.579613 -0.667025  C -1.895046 6.478205 -1.217376  H -3.911620 5.952705 -0.649516  H 0.197606 6.718007 -1.680382  H -2.186055 7.415043 -1.682963  C -1.718687 0.980027 2.875722  C -1.456000 2.418435 2.410192  H -0.797861 0.505086 3.224652  H -2.430278 0.946558 3.706827  H -0.795291 2.952466 3.102359  H -2.391069 2.984704 2.355006  C 1.107652 2.360969 0.860162  C 1.609321 1.104479 1.605654  H 1.448507 3.273588 1.360604  H 1.470756 2.398283 -0.171119  H 1.929894 1.370904 2.617141  H 0.841781 0.333073 1.700301  C -0.048643 1.326512 -2.348911  O 0.652877 2.330761 -2.264978  O 0.209335 0.108331 -2.520765  H -1.221203 1.540426 -2.231271  Ag 2.292482 -0.417069 -1.490898  N 1.742728 -2.859498 -2.917002  O 1.347672 -3.686817 -3.717220  O 1.104426 -2.677636 -1.774709  O 2.755907 -2.134779 -3.094193  Ag -1.040492 -2.444930 -1.921831  C -3.775546 -1.131940 -1.991969  O -3.389802 0.016402 -1.616464  O -3.168597 -2.227326 -1.918092  H -4.788731 -1.153093 -2.423900 |
| --- | --- |
|  |  |

| **INT4-1, Cartesian coordinates:**  Pd 2.403281 -0.787400 0.047295  P 2.435875 1.476323 0.774687  P 0.910866 -1.148349 1.701563  P -3.016878 0.679995 0.275741  C 3.996063 2.391051 1.003427  C 5.186239 1.828520 0.516765  C 4.024454 3.640361 1.647026  C 6.393271 2.512756 0.681985  H 5.164543 0.875252 -0.000889  C 5.232480 4.315120 1.807164  H 3.109313 4.092338 2.020965  C 6.418318 3.750195 1.325958  H 7.313230 2.075058 0.305649  H 5.250558 5.280098 2.305333  H 7.358988 4.277970 1.453434  C 1.359412 2.450364 -0.356454  C 1.123271 1.946721 -1.648059  C 0.704998 3.625834 0.047065  C 0.231172 2.593227 -2.506949  H 1.631642 1.046993 -1.981871  C -0.183861 4.269598 -0.813471  H 0.875199 4.041910 1.036376  C -0.429540 3.749963 -2.087203  H 0.057315 2.191502 -3.501266  H -0.698193 5.166616 -0.485019  H -1.131671 4.248583 -2.748430  C -3.278524 2.484940 0.228169  C -3.839242 3.057346 -0.923710  C -2.918800 3.318254 1.301854  C -4.042765 4.436212 -0.999927  H -4.116010 2.426415 -1.764368  C -3.127366 4.694755 1.224755  H -2.484585 2.900209 2.205404  C -3.688782 5.255471 0.073778  H -4.479637 4.867466 -1.895684  H -2.853170 5.329526 2.062434  H -3.851428 6.327766 0.016233  C -4.139762 -0.046631 1.524274  C -4.815490 0.723749 2.482466  C -4.300333 -1.444066 1.523660  C -5.625887 0.101250 3.433647  H -4.727885 1.805171 2.480719  C -5.102394 -2.060824 2.483426  H -3.809582 -2.051921 0.766593  C -5.765959 -1.288130 3.439964  H -6.153688 0.705355 4.165791  H -5.221746 -3.140223 2.472742  H -6.401315 -1.766884 4.179194  C 0.961284 -2.729072 2.606989  C 1.840441 -3.752152 2.223009  C 0.127092 -2.914965 3.724665  C 1.879821 -4.947037 2.945177  H 2.487295 -3.616275 1.362517  C 0.171529 -4.109139 4.441033  H -0.557235 -2.132214 4.043516  C 1.047436 -5.126929 4.050706  H 2.561929 -5.735233 2.640931  H -0.475452 -4.246062 5.302495  H 1.079489 -6.057559 4.609525  C 1.547157 1.548357 2.437071  C 1.263873 0.143045 3.006327  H 0.618615 2.107054 2.302791  H 2.168352 2.110569 3.140201  H 0.456727 0.169922 3.748067  H 2.154221 -0.239503 3.518137  C -0.877752 -0.954007 1.212850  C -1.313580 0.501992 1.011619  H -1.492614 -1.435523 1.980464  H -0.991061 -1.534686 0.290234  H -1.299166 1.036948 1.966067  H -0.651694 1.027084 0.317326  H 2.152055 -2.331787 -0.434110  Ag -2.986999 -0.446051 -1.800428  N -1.555758 -2.157593 -3.508797  O -1.002162 -2.772832 -4.395382  O -1.029558 -2.035932 -2.325041  O -2.686887 -1.581492 -3.671928  Ag 1.188531 -2.188191 -2.170985  C 3.942312 -0.927777 -2.582900  O 3.906516 -0.481037 -1.385640  O 3.060268 -1.544049 -3.212874  H 4.887117 -0.715940 -3.108957 | **TS1, Cartesian coordinates:**  Pd 0.492480 0.350010 0.787577  P 0.938211 -1.688577 -0.225916  P 1.371766 1.262068 -1.082398  P -1.924867 0.925681 -0.350537  C 2.578150 -2.393715 0.148574  C 2.815082 -3.769196 -0.012837  C 3.624906 -1.547772 0.550174  C 4.090544 -4.285046 0.212666  H 2.006319 -4.433871 -0.303134  C 4.899835 -2.071128 0.768725  H 3.439673 -0.488733 0.708269  C 5.132892 -3.437765 0.600155  H 4.269682 -5.349230 0.091181  H 5.705485 -1.413951 1.082491  H 6.123438 -3.845228 0.779600  C -0.295962 -2.974920 0.102906  C -0.881741 -3.742765 -0.914450  C -0.664394 -3.186503 1.443541  C -1.821367 -4.721978 -0.590528  H -0.613101 -3.587758 -1.954887  C -1.600832 -4.169855 1.757788  H -0.227670 -2.581855 2.233944  C -2.177511 -4.938360 0.742347  H -2.274554 -5.314023 -1.379821  H -1.882198 -4.333072 2.793824  H -2.907020 -5.703998 0.989936  C -3.147843 -0.353493 -0.815677  C -3.761552 -0.440503 -2.076671  C -3.467710 -1.306969 0.165705  C -4.673487 -1.462660 -2.347964  H -3.544845 0.291809 -2.849080  C -4.389832 -2.317183 -0.103587  H -2.985485 -1.263411 1.138489  C -4.990737 -2.399517 -1.361881  H -5.143501 -1.519830 -3.325775  H -4.626224 -3.047007 0.664272  H -5.704438 -3.190449 -1.574049  C -2.899700 2.364642 0.240685  C -4.240361 2.573101 -0.121620  C -2.259772 3.300616 1.069969  C -4.920795 3.703314 0.331054  H -4.755445 1.849342 -0.746684  C -2.941625 4.435658 1.512997  H -1.232269 3.138995 1.384031  C -4.272713 4.637436 1.144507  H -5.959550 3.853847 0.050885  H -2.437012 5.150817 2.156232  H -4.807771 5.514329 1.497269  C 2.837501 2.323181 -0.952295  C 3.341402 2.685467 0.305948  C 3.468783 2.787872 -2.119779  C 4.464764 3.509308 0.394455  H 2.857347 2.325239 1.209227  C 4.592401 3.606750 -2.022872  H 3.089987 2.515231 -3.101756  C 5.089753 3.967817 -0.766804  H 4.850046 3.789936 1.370045  H 5.078649 3.963886 -2.925732  H 5.964913 4.606771 -0.695557  C 0.957936 -1.353397 -2.055304  C 1.788667 -0.084794 -2.311899  H -0.082853 -1.222728 -2.368615  H 1.375207 -2.206730 -2.600007  H 1.650096 0.283656 -3.334466  H 2.854526 -0.297997 -2.176865  C 0.045182 2.295296 -1.869689  C -1.293487 1.551601 -2.012642  H 0.415302 2.641831 -2.842948  H -0.069069 3.180517 -1.232433  H -2.030706 2.221867 -2.466980  H -1.181912 0.691270 -2.681759  C 0.226545 1.114207 3.102868  O -0.054643 -0.071909 2.870267  H 0.510704 1.732784 1.799850  O 0.345388 1.955312 3.933962 |
| --- | --- |
|  |  |

| **TS2, Cartesian coordinates:**  Pd 1.407448 0.414296 0.969951  P 1.746349 -1.265578 -0.509168  P 1.035081 1.771104 -0.852602  P -2.595819 0.348617 -0.515295  C 3.510556 -1.534993 -0.828447  C 3.928872 -2.618758 -1.617564  C 4.455349 -0.629952 -0.323266  C 5.282006 -2.786970 -1.900613  H 3.202879 -3.334478 -1.992878  C 5.809214 -0.804209 -0.611831  H 4.130914 0.197179 0.302699  C 6.221029 -1.879875 -1.399654  H 5.606049 -3.627671 -2.506140  H 6.539411 -0.106319 -0.214415  H 7.275217 -2.017777 -1.619339  C 1.008304 -2.861000 -0.081808  C -0.305557 -3.178762 -0.459264  C 1.718305 -3.721945 0.770319  C -0.896325 -4.352187 0.006070  H -0.879550 -2.525344 -1.105626  C 1.116596 -4.887717 1.237533  H 2.728667 -3.474914 1.079547  C -0.190307 -5.203435 0.857902  H -1.909094 -4.594198 -0.298727  H 1.666430 -5.544628 1.903590  H -0.656706 -6.111811 1.226348  C -3.220863 -1.007403 -1.563508  C -3.309303 -0.930848 -2.961334  C -3.650668 -2.176310 -0.913283  C -3.806432 -2.010222 -3.694919  H -3.008632 -0.030403 -3.487016  C -4.162031 -3.244520 -1.646974  H -3.577419 -2.247278 0.169029  C -4.232994 -3.166152 -3.040506  H -3.868779 -1.941886 -4.776697  H -4.502104 -4.138566 -1.132878  H -4.623197 -4.001930 -3.612881  C -4.061777 1.321812 -0.029238  C -5.228522 1.313986 -0.808612  C -3.994099 2.114303 1.128038  C -6.318231 2.097676 -0.433755  H -5.291637 0.689988 -1.694796  C -5.089066 2.900967 1.489315  H -3.098045 2.121106 1.741606  C -6.249026 2.892286 0.713483  H -7.223167 2.083637 -1.033678  H -5.036016 3.511871 2.385376  H -7.101803 3.498000 1.004665  C 2.114720 3.223817 -0.965861  C 2.890425 3.606120 0.138548  C 2.191151 3.952963 -2.164654  C 3.737395 4.711367 0.037351  H 2.815148 3.057081 1.070867  C 3.042043 5.051352 -2.256987  H 1.593774 3.665855 -3.026366  C 3.815654 5.430162 -1.155712  H 4.334103 5.009136 0.893943  H 3.102182 5.611590 -3.184917  H 4.477098 6.287818 -1.230292  C 1.034161 -0.722606 -2.135228  C 1.400873 0.748679 -2.368330  H -0.049557 -0.858856 -2.091133  H 1.419328 -1.362223 -2.935920  H 0.895390 1.152493 -3.251841  H 2.478691 0.844937 -2.536038  C -0.689452 2.438999 -1.065035  C -1.716191 1.488908 -1.704775  H -0.579373 3.327385 -1.697261  H -1.014902 2.783591 -0.077871  H -2.494021 2.088252 -2.189339  H -1.254964 0.886126 -2.490600  C 0.944167 -1.581736 3.097902  O 1.805344 -2.124072 3.721834  Ag -1.341812 -0.315749 1.391324  C 0.116665 2.332845 2.797258  O 1.267497 1.949082 2.384352  O -0.994697 1.907102 2.435585  H 0.163967 3.138285 3.549181  H 1.572186 -0.729541 2.230292  O -0.277388 -1.519098 2.948243 | **TS2-1, Cartesian coordinates:**  Pd 1.250471 0.374794 0.856595  P 1.838930 -1.347965 -0.487001  P 0.997356 1.584968 -1.061235  P -2.684260 0.249752 -0.478277  C 3.637616 -1.534583 -0.655711  C 4.163956 -2.570031 -1.448364  C 4.499905 -0.623251 -0.025231  C 5.542695 -2.680892 -1.615471  H 3.503182 -3.292930 -1.920302  C 5.880394 -0.742964 -0.198227  H 4.098438 0.164803 0.606423  C 6.399781 -1.766589 -0.992894  H 5.949562 -3.481563 -2.225831  H 6.545718 -0.040858 0.294959  H 7.473927 -1.858861 -1.123708  C 1.131872 -2.930578 0.044058  C -0.189711 -3.276222 -0.291409  C 1.860998 -3.744026 0.926453  C -0.766168 -4.427270 0.242204  H -0.784432 -2.660181 -0.958556  C 1.274478 -4.892045 1.458792  H 2.877537 -3.478223 1.199090  C -0.037484 -5.233910 1.120470  H -1.784526 -4.687655 -0.030440  H 1.842853 -5.518750 2.139298  H -0.490478 -6.127212 1.539885  C -3.490891 -1.120808 -1.385488  C -3.756597 -2.315710 -0.693984  C -3.859441 -1.020641 -2.738155  C -4.375488 -3.386072 -1.340191  H -3.481197 -2.409846 0.353770  C -4.470272 -2.096984 -3.383965  H -3.684679 -0.105000 -3.294658  C -4.728067 -3.280143 -2.687731  H -4.583219 -4.300060 -0.791133  H -4.750427 -2.007718 -4.429435  H -5.205879 -4.114435 -3.192628  C -3.975958 1.438283 0.024683  C -5.278819 1.384314 -0.494472  C -3.622545 2.442388 0.943781  C -6.220687 2.334682 -0.098726  H -5.562835 0.603439 -1.193486  C -4.570477 3.394706 1.320616  H -2.617019 2.505476 1.355203  C -5.867721 3.340023 0.805175  H -7.230919 2.286694 -0.494748  H -4.291491 4.173063 2.024577  H -6.605094 4.076500 1.111054  C 2.090583 3.031574 -1.111531  C 1.623777 4.282954 -0.676004  C 3.440637 2.876897 -1.474710  C 2.491850 5.372975 -0.651963  H 0.613011 4.401649 -0.301947  C 4.299401 3.973205 -1.445049  H 3.838010 1.907817 -1.762662  C 3.824374 5.223558 -1.040644  H 2.126999 6.336434 -0.309935  H 5.339152 3.849788 -1.732923  H 4.496727 6.075894 -1.014150  C 1.189746 -0.980983 -2.189693  C 1.438387 0.497455 -2.515511  H 0.120728 -1.210251 -2.188560  H 1.672889 -1.641680 -2.917176  H 0.874020 0.819929 -3.397255  H 2.496296 0.659038 -2.736989  C -0.720122 2.207688 -1.381167  C -1.759023 1.161843 -1.829463  H -0.604974 2.960886 -2.170199  H -1.032770 2.734836 -0.473626  H -2.529313 1.685082 -2.406328  H -1.328250 0.412635 -2.500972  C 0.416439 2.552804 1.944231  O -0.496541 3.323237 1.626152  O 1.635249 2.659007 2.154919  H 0.021972 1.408857 2.019260  Ag -1.431523 -0.549211 1.369131  C 0.800231 -1.377024 3.254521  O 1.680985 -0.781064 2.560757  O -0.436734 -1.458467 3.046152  H 1.186455 -1.885951 4.150061 |
| --- | --- |
|  |  |

| **TS2-2, Cartesian coordinates:**  Pd 1.589750 0.417846 0.572905  P 1.798592 -1.373682 -0.851382  P 1.016741 1.623382 -1.240289  P -2.513285 0.082216 -0.135956  C 3.438174 -2.150667 -0.692584  C 4.523944 -1.392653 -0.224238  C 3.628526 -3.491384 -1.064917  C 5.791835 -1.968769 -0.144476  H 4.376892 -0.364687 0.097364  C 4.899261 -4.059137 -0.982564  H 2.789146 -4.090740 -1.405911  C 5.979767 -3.299543 -0.524806  H 6.627554 -1.382708 0.225825  H 5.044333 -5.096687 -1.268347  H 6.966189 -3.748453 -0.455782  C 0.556666 -2.685170 -0.620529  C 0.305631 -3.102393 0.699881  C -0.146764 -3.278551 -1.679771  C -0.648353 -4.086180 0.950482  H 0.847568 -2.654060 1.525592  C -1.103154 -4.261889 -1.418912  H 0.045099 -2.994445 -2.709754  C -1.358634 -4.661312 -0.106857  H -0.843005 -4.393782 1.973473  H -1.652497 -4.708407 -2.241324  H -2.113915 -5.414716 0.091392  C -3.418550 -1.467412 -0.507922  C -3.716419 -1.905517 -1.808087  C -3.859092 -2.232949 0.584380  C -4.442660 -3.081006 -2.008654  H -3.393206 -1.337991 -2.674885  C -4.593585 -3.401069 0.382016  H -3.626601 -1.914687 1.597756  C -4.885346 -3.828391 -0.915420  H -4.669035 -3.407122 -3.019916  H -4.933554 -3.978037 1.237158  H -5.456601 -4.738520 -1.074045  C -3.773766 1.408763 -0.085428  C -4.987602 1.298324 -0.783753  C -3.492909 2.574972 0.646516  C -5.906576 2.346485 -0.753862  H -5.223595 0.393085 -1.335745  C -4.416651 3.622613 0.662956  H -2.555224 2.674964 1.184714  C -5.621825 3.509604 -0.032376  H -6.847658 2.252790 -1.288229  H -4.194562 4.522545 1.229273  H -6.342382 4.322092 -0.007915  C 1.692696 3.305185 -1.248884  C 3.036723 3.467257 -0.870936  C 0.930624 4.426648 -1.608478  C 3.615180 4.734331 -0.871653  H 3.621326 2.609504 -0.547977  C 1.516931 5.692408 -1.606256  H -0.114909 4.330882 -1.878530  C 2.855511 5.848013 -1.240322  H 4.652036 4.853792 -0.572543  H 0.923276 6.557804 -1.884849  H 3.304591 6.836701 -1.234584  C 1.663739 -0.756527 -2.616468  C 1.866713 0.765275 -2.663725  H 0.678482 -1.020086 -3.007005  H 2.408413 -1.274952 -3.227805  H 1.523123 1.190723 -3.613935  H 2.928363 1.015368 -2.561259  C -0.766313 1.743598 -1.712041  C -1.603720 0.446337 -1.726596  H -0.756098 2.175338 -2.720913  H -1.206325 2.484343 -1.040447  H -2.360990 0.549515 -2.510605  H -1.018154 -0.444544 -1.964113  C 1.784906 -0.486651 3.433237  O 1.767368 -0.257530 4.578623  O 2.277924 -0.759539 2.361657  H -0.007485 -0.498090 3.060571  Ag -1.089771 -0.145607 1.797690  C 0.678782 2.864794 1.887948  O 1.669482 2.038263 1.855340  O -0.313616 2.848047 1.149915  H 0.788000 3.638592 2.666977 | **TS3, Cartesian coordinates:**  Pd 1.839508 0.999965 0.411184  P 2.237274 -0.010695 -1.633314  P 0.500642 2.457969 -0.693621  P -3.104479 -0.028268 -0.653549  C 3.984721 -0.247914 -2.053506  C 4.703509 0.750719 -2.729883  C 4.653062 -1.388771 -1.576893  C 6.072459 0.597504 -2.948020  H 4.207411 1.649755 -3.086428  C 6.021624 -1.533408 -1.798909  H 4.111578 -2.154269 -1.029187  C 6.731635 -0.543862 -2.484481  H 6.623116 1.368521 -3.478507  H 6.533010 -2.418840 -1.433378  H 7.797749 -0.660221 -2.654670  C 1.411545 -1.633691 -1.768537  C 0.404144 -1.979693 -0.852489  C 1.722190 -2.514604 -2.817919  C -0.284308 -3.186438 -0.977962  H 0.148989 -1.307567 -0.038415  C 1.023282 -3.715714 -2.946241  H 2.513752 -2.272553 -3.521410  C 0.021286 -4.051912 -2.030007  H -1.050329 -3.438508 -0.252776  H 1.266775 -4.392981 -3.759546  H -0.519009 -4.987615 -2.135088  C -3.341165 -1.528997 -1.672593  C -3.864982 -2.671876 -1.045140  C -2.981210 -1.598985 -3.028111  C -4.027500 -3.858833 -1.759167  H -4.137195 -2.637022 0.007049  C -3.134906 -2.790980 -3.737165  H -2.574055 -0.733403 -3.541341  C -3.657166 -3.921469 -3.104837  H -4.435781 -4.734192 -1.262719  H -2.847649 -2.835373 -4.783662  H -3.776832 -4.847631 -3.659400  C -4.526473 1.083869 -0.922713  C -5.202720 1.134128 -2.152130  C -4.912547 1.941708 0.119485  C -6.247226 2.039496 -2.335349  H -4.927842 0.457165 -2.956395  C -5.953662 2.851584 -0.072642  H -4.409031 1.891511 1.082367  C -6.620512 2.900264 -1.298635  H -6.774906 2.068962 -3.284211  H -6.252055 3.508962 0.738722  H -7.438239 3.600102 -1.443810  C 0.745980 4.137949 -0.050816  C 2.058018 4.646499 -0.015501  C -0.308144 4.900210 0.475805  C 2.304432 5.910092 0.514691  H 2.890298 4.048083 -0.379387  C -0.051929 6.164245 1.009130  H -1.323615 4.519668 0.486988  C 1.248746 6.670766 1.026639  H 3.318936 6.295991 0.539841  H -0.870888 6.750072 1.415264  H 1.442543 7.653182 1.446532  C 1.448309 1.056354 -2.966432  C 1.123632 2.469925 -2.454908  H 0.539467 0.530374 -3.270624  H 2.103925 1.093327 -3.841918  H 0.395889 2.975631 -3.100013  H 2.027270 3.088934 -2.441516  C -1.313479 2.152524 -0.740368  C -1.686397 0.852226 -1.477144  H -1.808014 3.013041 -1.202393  H -1.601790 2.121774 0.314404  H -1.926434 1.065677 -2.523335  H -0.874200 0.122764 -1.467019  C 0.273326 1.036455 2.692223  O 0.815185 0.324459 3.504668  O -0.833311 1.402294 2.305950  H 1.221230 1.655004 1.945821  Ag -2.504846 -0.543032 1.590301  N -1.176030 -2.202909 3.462081  O -0.608792 -2.768775 4.373159  O -0.653285 -2.167833 2.256504  O -2.291937 -1.617785 3.583388  Ag 1.514512 -2.174655 2.179133  C 4.132510 -0.831371 1.662654  O 3.604652 0.265854 1.319836  O 3.596489 -1.963431 1.781414  H 5.212360 -0.776401 1.872886 |
| --- | --- |
|  |  |

| **TS3’, Cartesian coordinates:**  Pd 2.047255 0.745647 0.469301  P 2.502009 -0.317218 -1.562413  P 0.867238 2.271213 -0.704834  P -3.058143 0.102623 -0.862677  C 4.271288 -0.489802 -1.928933  C 4.977377 0.540671 -2.569761  C 4.962757 -1.616859 -1.455027  C 6.356334 0.435192 -2.750536  H 4.462298 1.426827 -2.931413  C 6.340736 -1.716005 -1.640467  H 4.425248 -2.416284 -0.953592  C 7.038595 -0.692075 -2.286462  H 6.896358 1.231327 -3.254355  H 6.869518 -2.593360 -1.280161  H 8.112214 -0.772289 -2.428019  C 1.741054 -1.962051 -1.752181  C 0.771055 -2.393783 -0.833529  C 2.061454 -2.774446 -2.853325  C 0.117637 -3.612726 -1.012385  H 0.532306 -1.783728 0.033798  C 1.407884 -3.994032 -3.028919  H 2.826468 -2.463807 -3.559699  C 0.435553 -4.409694 -2.113769  H -0.631997 -3.926145 -0.294386  H 1.661185 -4.621607 -3.878440  H -0.072072 -5.358812 -2.256804  C -3.117418 -1.579840 -1.565481  C -3.529891 -2.648788 -0.755085  C -2.706359 -1.832971 -2.888286  C -3.540805 -3.949333 -1.265252  H -3.819491 -2.484415 0.277685  C -2.722253 -3.131969 -3.390826  H -2.381845 -1.020386 -3.532409  C -3.143192 -4.191219 -2.580177  H -3.854551 -4.768644 -0.625787  H -2.404839 -3.318738 -4.412464  H -3.155705 -5.203153 -2.974914  C -4.196556 1.191811 -1.791612  C -4.854975 0.792689 -2.963795  C -4.403613 2.490025 -1.291123  C -5.691360 1.688196 -3.633595  H -4.732366 -0.214449 -3.347750  C -5.230357 3.383332 -1.970920  H -3.934557 2.796972 -0.358517  C -5.874747 2.982916 -3.144722  H -6.204912 1.368719 -4.535651  H -5.386758 4.382589 -1.575098  H -6.529489 3.673822 -3.667445  C 1.172213 3.978509 -0.165506  C 2.511209 4.383220 -0.016767  C 0.138282 4.873548 0.144675  C 2.808219 5.673337 0.415239  H 3.320427 3.683652 -0.215450  C 0.443501 6.163614 0.581960  H -0.901904 4.577793 0.061257  C 1.773660 6.565083 0.714733  H 3.843772 5.978798 0.530210  H -0.360608 6.852397 0.822868  H 2.005557 7.568528 1.058857  C 1.743566 0.732558 -2.922164  C 1.492677 2.178585 -2.463856  H 0.806669 0.235299 -3.186256  H 2.383337 0.705025 -3.810023  H 0.792918 2.698445 -3.128887  H 2.425873 2.752773 -2.468349  C -0.959037 2.032410 -0.763405  C -1.350510 0.671342 -1.352181  H -1.417074 2.848261 -1.333082  H -1.277475 2.124104 0.280726  H -1.284411 0.686128 -2.444462  H -0.689396 -0.121698 -0.989896  C 0.527185 1.502353 2.967048  O 1.039350 0.890711 3.857253  O -0.446510 2.089749 2.546125  H 1.551980 1.565741 1.877337  Ag -3.206487 0.279702 1.498502  N -2.023110 -2.441905 2.475265  O -1.519376 -3.425141 1.930374  O -1.243672 -1.555198 3.052036  O -3.246081 -2.206601 2.490677  Ag 0.868136 -1.890153 2.734420  C 3.794415 -1.364496 2.091616  O 3.624294 -0.301839 1.428400  O 2.944899 -2.194586 2.501256  H 4.846163 -1.593637 2.327402  O -2.729353 0.755358 3.607025  H -2.273743 -0.091159 3.818533  H -2.006958 1.415212 3.544970 | **TS3-1, Cartesian coordinates:**  Pd 0.754093 0.066835 0.364517  P 0.867990 -1.862022 -0.880446  P 0.453939 1.125707 -1.591927  P -3.221801 0.603201 -0.100214  C 2.058666 -3.101187 -0.305442  C 3.175382 -2.714018 0.451958  C 1.891244 -4.449194 -0.672007  C 4.125711 -3.662664 0.824292  H 3.305612 -1.682837 0.752654  C 2.846860 -5.390367 -0.294337  H 1.022290 -4.762198 -1.243289  C 3.963341 -4.998346 0.450481  H 4.991982 -3.351612 1.399421  H 2.719015 -6.430042 -0.580342  H 4.705693 -5.735975 0.740294  C -0.758655 -2.689702 -0.906673  C -1.602046 -2.654729 -2.028759  C -1.209480 -3.315255 0.270715  C -2.881640 -3.209327 -1.966037  H -1.268047 -2.214041 -2.962490  C -2.487869 -3.869733 0.326310  H -0.565806 -3.376985 1.141868  C -3.330452 -3.806290 -0.786568  H -3.528565 -3.172515 -2.836808  H -2.823902 -4.344031 1.243384  H -4.333630 -4.216879 -0.735839  C -4.687066 -0.419631 -0.501678  C -5.271781 -1.142130 0.552501  C -5.229320 -0.539876 -1.790737  C -6.375593 -1.962610 0.323827  H -4.858689 -1.070151 1.555888  C -6.327322 -1.371950 -2.019689  H -4.805464 0.003519 -2.628713  C -6.901779 -2.084227 -0.964960  H -6.819073 -2.511211 1.149612  H -6.736188 -1.458057 -3.022359  H -7.757251 -2.728444 -1.145770  C -3.828804 2.269875 0.348728  C -5.043492 2.775155 -0.139754  C -3.018056 3.072802 1.170010  C -5.439145 4.072495 0.186084  H -5.685044 2.153935 -0.758843  C -3.417352 4.373760 1.483702  H -2.079935 2.682664 1.558144  C -4.626499 4.872968 0.994299  H -6.384195 4.457122 -0.186202  H -2.791073 4.991265 2.121412  H -4.940594 5.881035 1.248635  C 1.599234 2.520573 -1.777928  C 2.779116 2.398418 -2.530650  C 1.334871 3.717941 -1.086095  C 3.675571 3.464759 -2.594010  H 3.027304 1.471434 -3.031483  C 2.236486 4.776805 -1.156182  H 0.443950 3.819661 -0.476629  C 3.408802 4.651404 -1.907548  H 4.587579 3.361071 -3.173773  H 2.027285 5.696989 -0.619277  H 4.113567 5.476139 -1.954881  C 1.286419 -1.385794 -2.625194  C 0.631170 -0.053717 -3.028151  H 1.015577 -2.195853 -3.310048  H 2.373712 -1.281570 -2.613706  H -0.381018 -0.206027 -3.414841  H 1.201864 0.440107 -3.820030  C -1.229503 1.877412 -1.762514  C -2.438784 0.921689 -1.766701  H -1.190693 2.429732 -2.710000  H -1.329937 2.628061 -0.973043  H -3.213597 1.380266 -2.389486  H -2.209572 -0.053231 -2.206250  C 0.828305 -1.714559 2.944130  O 0.669744 -2.699192 3.555384  O 1.414234 -0.860579 2.332849  H -0.945312 -1.100791 2.867146  Ag -1.890873 -0.411049 1.645922  N 4.783225 -0.449165 -0.934677  O 5.510984 -1.223188 -1.532464  O 3.627543 -0.133570 -1.350776  O 5.173458 0.068828 0.190992  Ag 3.641553 1.345552 1.000486  C 1.061026 2.598085 2.075258  O 0.271912 1.879856 1.377368  O 2.308808 2.573439 2.119791  H 0.558564 3.346503 2.708985 |
| --- | --- |
|  |  |
| **AgOOCH, Cartesian coordinates:**  Ag 0.740372 0.000117 -0.000001  C -1.866363 -0.000589 -0.000221  O -1.288459 -1.124404 0.000061  O -1.290095 1.124276 0.000061  H -2.970898 -0.000921 0.000402 | **AgNO_3_, Cartesian coordinates:**  N -1.630188 -0.000047 0.000008  O -0.952198 -1.101339 0.000366  O -2.844294 0.000913 -0.000381  O -0.950936 1.100312 0.000366  Ag 1.050867 0.000026 -0.000061 |
|  |  |
| **H_2_O, Cartesian coordinates:**  O 0.000000 0.000000 0.119711  H 0.000000 0.761599 -0.478844  H -0.000000 -0.761599 -0.478844 | **CO_2_, Cartesian coordinates:**  C -0.000000 0.000000 -0.000021  O -0.000000 -0.000000 1.169328  O 0.000000 0.000000 -1.169313 |
|  |  |

**References**

1. M. Gutiérrez-Blanco, C. Stein, C. Alfonso, E. Guillamón, V. Safont, I. Sorribes, H. Junge, M. Beller, R. Llusar, Selective Dehydrogenation of Formic Acid Catalyzed by Air-Stable Cuboidal PN Molybdenum Sulfide Clusters. *ChemCatChem* **2023**, *15*, e202300740.
2. C. Fellay, P. Dyson, G. Laurenczy, A Viable Hydrogen-Storage System Based On Selective Formic Acid Decomposition with a Ruthenium Catalyst. *Angew. Chem., Int. Ed.* **2008**, *47*, 3966-3968.
3. J. Kothandaraman, M. Czaun, A. Goeppert, R. Haiges, J.-P. Jones, R. May, G. Prakash, G. Olah, Amine-Free Reversible Hydrogen Storage in Formate Salts Catalyzed by Ruthenium Pincer Complex without pH Control or Solvent Change. *ChemSusChem* **2015**, *8*, 1442-1451.
4. N. Onishi, R. Kanega, E. Fujita, Y. Himeda, Carbon Dioxide Hydrogenation and Formic Acid Dehydrogenation Catalyzed by Iridium Complexes Bearing Pyridyl-pyrazole Ligands: Effect of an Electron-donating Substituent on the Pyrazole Ring on the Catalytic Activity and Durability. *Adv. Synth. Catal.* **2019**, *361*, 289-296.
5. A. Boddien, B. Loges, F. Gärtner, C. Torborg, K. Fumino, H. Junge, R. Ludwig, M. Beller, Iron-Catalyzed Hydrogen Production from Formic Acid. *J. Am. Chem. Soc.* **2010**, *132*, 8924-8934.
6. T. Zell, B. Butschke, Y. Ben-David, D. Milstein, Efficient Hydrogen Liberation from Formic Acid Catalyzed by a WellDefined Iron Pincer Complex under Mild Conditions. *Chem.-Eur. J.* **2013**, *19*, 8068-8072.
7. Gaussian 16, Revision A.03, Gaussian, Inc.: Wallingford, CT, **2016**.

8. P. Stephens, F. Devlin, C. Chabalowski, M. Frisch, Ab Initio Calculation of Vibrational Absorption and Circular Dichroism Spectra Using Density Functional Force Fields. *J. Phys. Chem.* **1994**, *98*, 11623.

9. R. Ditchfield, W. Hehre, J. Pople, Self-Consistent Molecular-Orbital Methods. IX. An Extended Gaussian-Type Basis for Molecular-Orbital Studies of Organic Molecules. *J. Chem. Phys.* **1971**, *54*, 724.

10. W. Hehre, R. Ditchfield, J. Pople, Self-Consistent Molecular Orbital Methods. XII. Further Extensions of Gaussian-Type Basis Sets for Use in Molecular Orbital Studies of Organic Molecules. *J. Chem. Phys.* **1972**, *56*, 2257.

11. U. Wedig, M. Dolg, H. Stoll, H. Preuss, in Quantum Chemistry: The Challenge of Transition Metals and Coordination Chemistry Ed. A. Veillard, Reidel, and Dordrecht, **1986**, 79.

12. A. Marenich, C. Cramer, D. Truhlar, Universal Solvation Model Based on Solute Electron Density and on a Continuum Model of the Solvent Defined by the Bulk Dielectric Constant and Atomic Surface Tensions. *J. Phys. Chem. B* **2009**, *113*, 6378.

13. C. Legault, CYLview, version 1.0b, Université de Sherbrooke: Sherbrooke, QC, Canada, **2009**. http://www.cylview.org.
